# Supplementary material for: Lipopeptide-mediated Cas9 RNP delivery: A promising broad therapeutic strategy for safely removing deep-intronic variants in ABCA4
Source: Mol Ther Nucleic Acids. 2024 Sep 26;35(4):102345. doi: 10.1016/j.omtn.2024.102345 (PMC11531624; doi:10.1016/j.omtn.2024.102345)
Supplement: Document S1. Figures S1–S19 and Tables S1–S3 and S6–S9 [file mmc1.pdf]

OMTN, Volume *35*

Supplemental information

Lipopeptide-mediated Cas9 RNP delivery: A promising broad therapeutic strategy for safely removing deep-intronic variants in *ABCA4*

Irene Vázquez-Domínguez, Mert Öktem, Florian A. Winkelaar, Thai Hoang Nguyen, Anita D.M. Hoogendoorn, Eleonora Roschi, Galuh D.N. Astuti, Raoul Timmermans, Nuria Suárez-Herrera, Ilaria Bruno, Albert Ruiz-Llombart, Joseph Brealey, Olivier G. de Jong, Rob W.J. Collin, Enrico Mastrobattista, and Alejandro Garanto

Supplemental Material

Methods

In vitro cytotoxicity assay

HEK293T stoplight,[1] HeLa, and eGFP HEPA 1-6 cells were cultured in a Greiner Bio-One 96 well-plate at a density of 3 x 10^4 cells per well. After a 24-hour incubation period, cells were transfected using a 100 µl solution of RPNC (RNP/C18:1-LAH5). These were prepared presence of 20 nM RNP and 1 µM to 20 µM lipopeptide and following at a ratio of RNP:lipopeptide ranging from 1:50 to 1:1000. As a reference, positive control wells were treated with a 100 µL of medium enriched with 5% Triton X-100. This experimental procedure was conducted in triplicate for each condition.

Twenty-four hours after transfection, cell viability was assessed by treating the cells with a 20 µl solution of CellTiter 96® Aqueous one solution cell proliferation assay (MTS) reagent (Promega Corporation, USA), following the manufacturer's recommended protocol. Fluorescence intensity was quantified with an absorbance set of 490 nm using an IMark™ Microplate Reader (Bio-Rad, Hercules, California, USA). Data was normalized by excluding reference positive control signals from negative control and treated samples.

Transfection of pX458 constructs in HEK293T

A total of 400,000 cells of HEK293T were transfected with the pX458 vector (Addgene, #48138) in which the designed gRNAs were introduced after the U6 promoter. The transfection was done using FuGENE®-HD (Promega, Madison, WI, USA) following a 3:1 FuGENE®-HD Transfection Reagent: DNA ratio. The FuGENE®-HD/DNA mixture was prepared in Opti-MEM reduced serum medium (Gibco, Waltham, MA, USA). Samples were harvested 48 h after transfection to proceed with DNA analysis.

Protein isolation and Western blot analysis in PPCs

After 12 days of RPNC delivery, PPCs were harvested (between day 38-39 of differentiation) for protein analysis. PPCs cells were washed in 1x PBS, mechanically detached in 200 µl of RIPA (50 mM Tris pH 7.5, 1 mM EDTA, 150 mM NaCl, 0.5% Na-Deoxycholate, 1% NP-40, 0.75% SDS) supplemented with cOmplete™ Mini Protease Inhibitor Cocktail (Roche,Manheim, Germany), and sonicated for 15 s/sample. Protein concentration was determined by using the Pierce™ BCA Protein Assay Kit (Thermo Fisher Scientific, Waltham, MA, USA) according to manufacturer's indications. A total of 55 µg of protein per condition were loaded on 4-15% Mini-PROTEAN® TGX Stain-Free pre-cast gels (Bio-Rad) and electrophoresed for 45 min at 200 V in Tris-Glycine-SDS buffer (Bio-Rad, Hercules, CA, USA). Transfer to Trans-Blot Turbo Mini 0.2 µm nitrocellulose membranes (Bio-Rad) was conducted using the Trans-Blot Turbo Transfer System (Bio-Rad). After blocking in 5% blotto non-fatty milk diluted in 1x PBS during 1 h at RT, membranes were incubated with the primary antibody overnight at 4°C. The next day, blots were washed 3 times for 5 minutes in 1x PBS supplemented with 0.2% of Tween. Then, blots were incubated with the corresponding IRDye secondary antibody for 1 h at RT in the dark. Prior reveling the membrane, membranes were washed with 1x PBS – 0.2% Tween as previously indicated. Membranes were developed in the Odyssey Imaging System (Li-COR Biosciences, Lincoln, NE, USA). Intensity of the ABCA4 protein bands was semi-quantified using Fiji software 1.53t, with normalization to β-tubulin bands and further normalization to the non-treated (NT) condition. Employed antibodies and their dilution conditions can be found on **Table S9**.

Analysis of Whole Genome sequencing (WGS) and long-read sequencing

To confirm both on-target and off-target effects, we analyzed two DNA samples each from control and patient PPCs. These samples included the non-treated (NT) condition and the

treated with RPNC including gRNAs 30-1 and 30-2. None of the samples underwent treatment with CHX.

Long-read (LR) sequencing for on-target validation:

To assess the on-target effects after RPNC delivery with the two gRNAs, targeted PacBio long-read sequencing of the amplicon was performed. We amplified intron 30 from PPC DNA as previously described in the main manuscript. The resulting amplicons of 2936 bp in the non-treated samples and 2936 bp together with an amplicon 323 bp in the treated samples were assessed on an agarose gel and measured in Qubit. A total amount of 350 - 500 ng was used for long-read sequencing. The PCR product was then purified using AMPure PB beads (Pacific Biosciences, Menlo Park, CA, USA) with a bead ratio of 1.5x. The library was prepared according to the protocol *"Procedure and Checklist-Preparing SMRTbell Libraries using PacBio Barcoded Adapters for Multiplex SMRT Sequencing"* (Pacific Biosciences, part number 100-538-700-02) as published previously.[2] Briefly, after measuring the purified sample with Qubit, an equimolar amount of amplicons were used to perform a one-step end and adapter ligation using barcoded hairpin adapters), followed by equimolar pooling. The pool was purified with AMPure PB Beads, followed by DNA damage repair, exonuclease digestion, and two additional rounds of AMPure PB Beads purification.

Polymerase bound SMRT-bell complexes and SMRT sequencing were then performed. To generate the complexes, the Sample Setup option in SMRTLink (Pacific Biosciences) was used. Briefly, sequencing primers were conditioned and annealed to the SMRTbell library, followed by dilution and binding of the sequencing polymerase. The polymerase-bound complex was purified using AMPure PB beads and the concentration measured using Qubit. An internal control sample was diluted and added to the polymerase-bound complex. Sequencing was then performed using the Run Design option in SMRTLink. Libraries were loaded at a plate concentration of 4.5 pM. All runs were sequenced using a movie time of 20 h

per SMRTcell and included pre-extension. Sequencing was performed on a Sequel I system (Pacific Biosciences) using ICS version 8.0. Finally, targeted long-read sequencing was performed on a Sequel II machine (Pacific Biosciences). Subreads were demultiplexed using lima V.2.5.0. The subreads were combined into a consensus sequence using CCS V.6.3.0, these reads were filtered for RQ 0.99 to obtain HiFi reads. The HiFi reads were then mapped along the GRCh38 reference genome using pbmm2 V1.8.0 with the "--preset ISOSEQ" parameter.

Whole genome-sequencing (WGS) for off-target analysis:

A minimum of 1 µg of DNA of the aforementioned samples were sent to BGI Genomics Poland. The library type used was for amplicon ≤ 800 bp using the sequencing platform DNBseq^{TM}. Prediction of the possible off-targets for gRNA 30.1 and gRNA 30.2 was done using the web tool: Off-Spotter (https://cm.jefferson.edu/Off-Spotter/), indicating the none position is using as sit. From the output list all targets with 3 mismatches or less were analyzed as potential off-targets (**Table S4**). Then, the predicted SVs, SNVs, and off-target areas were manually inspected using Integrative Genomics Viewer 2.4 software [3], by aligning the reads with the Human Reference Genome (GRCh38.p14).

Repair profile analysis

To assess the mutational profile after genome editing of intron 30 of *ABCA4*, we first extracted correctly mapped reads from our PacBio data. We then filtered these reads by size, removing all those > 1000 nt. This resulted in 19136 and 10399 reads left in the data from the isogenic control and patient PPCs, respectively. Subsequently, we filtered out all those reads that were identified fewer than five times, leaving 9444 and 4554 reads explaining 34 and 17 different editing events in the isogenic control and patient edited PPC cells, respectively. In total, 44 different repair events were identified, of which seven events were shared between both lines.

The top 3 events explained ~82% of all reads and they were shared by both lines at similar ratios (**Table S5**).

Supplemental Figures

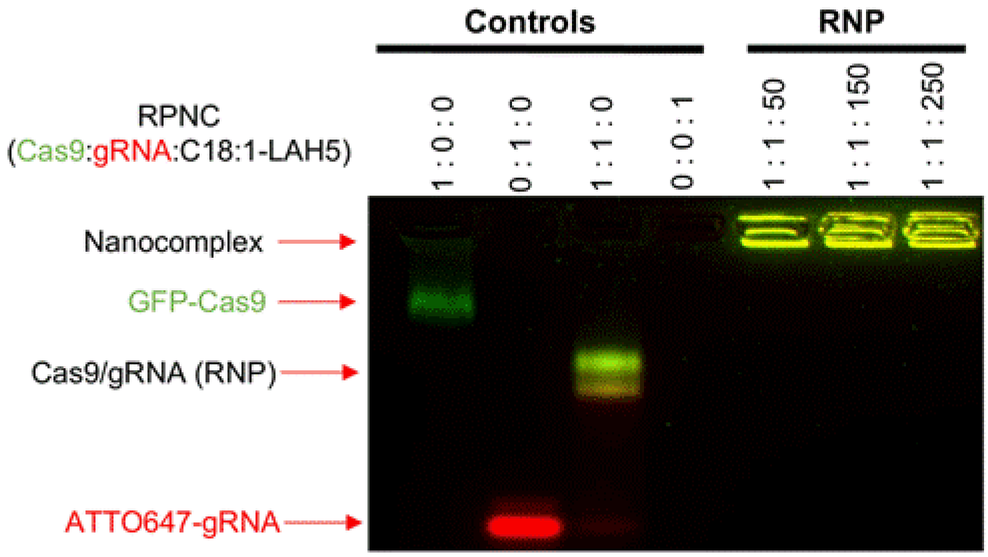

Figure S1. Electrophoretic mobility shift assay showing the effect of increasing C18:1-LAH5 lipopeptide concentration on RPNC formation. The efficient capacity of C18:1-LAH5 lipopeptide to form complexes with RNP was tested in escalating lipopeptide concentrations through an agarose-based electrophoretic mobility shift assay (EMSA), employing equivalent quantities of ATTO647-gRNA and GFP-Cas9.

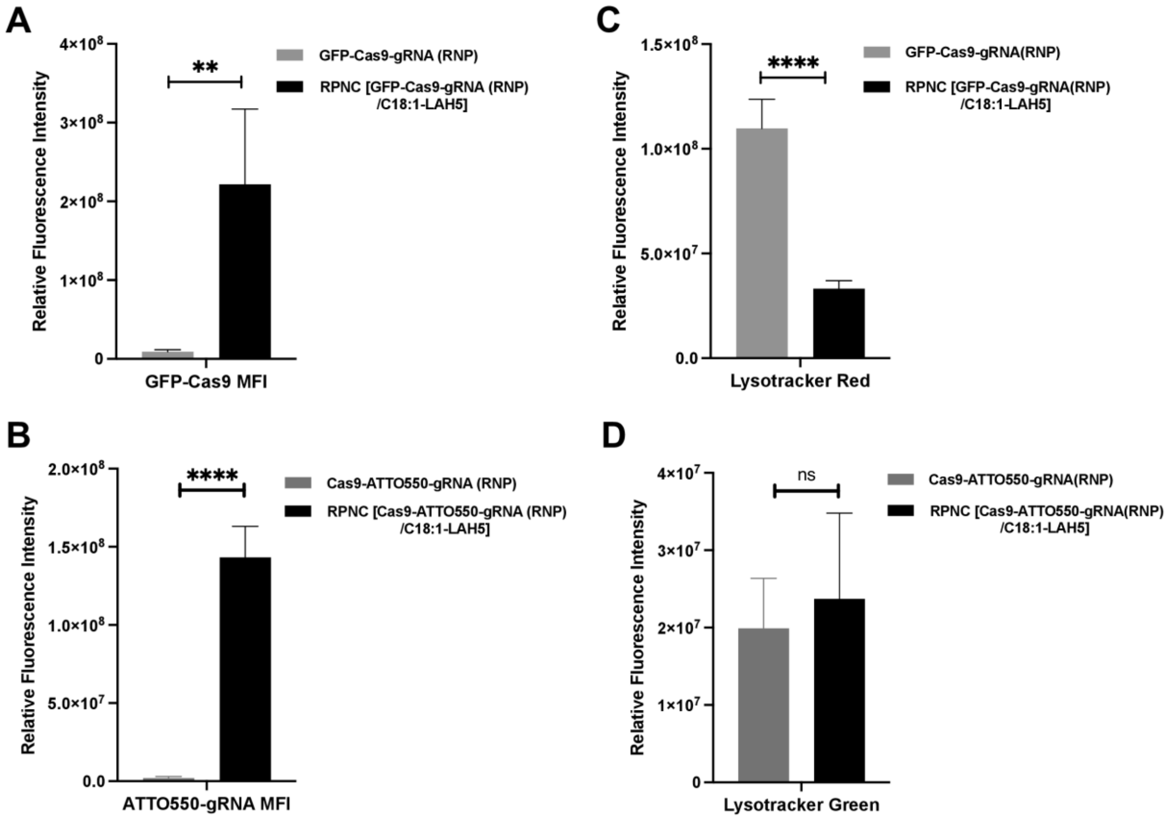

Figure S2. Relative cellular uptake of fluorescently labeled RNPs in the absence and presence of C18:1-LAH5. HeLa cells were treated with RPNCs, **A)** including GFP-Cas9/gRNA/C18:1-LAH5, **B)** non-labeled Cas9/ATTO 550-gRNA/C18:1-LAH5, with a molar ratio of 1:150. In addition, the cells were treated with **C)** Lysotracker red, and **D)** Lysotracker green. Control experiments were conducted using RNP preparations without the inclusion of C18:1-LAH5. The fluorescence of each material was imaged through confocal microscopy. The relative fluorescence intensity (RMFI) was quantified by measuring the total fluorescence intensity of the whole image using ImageJ, and RMFI was calculated based the following formula: *RMFI = fluorescence intensity of measured material value – background fluorescence intensity value.* The difference in RMFI of each material in non-lipopeptide treated and lipopeptide treated sample were compared using a two tailed unpaired t-test. (non-significant (ns), * $p < 0.05$, ** $p < 0.01$, *** $p < 0.001$, **** $p < 0.0001$).

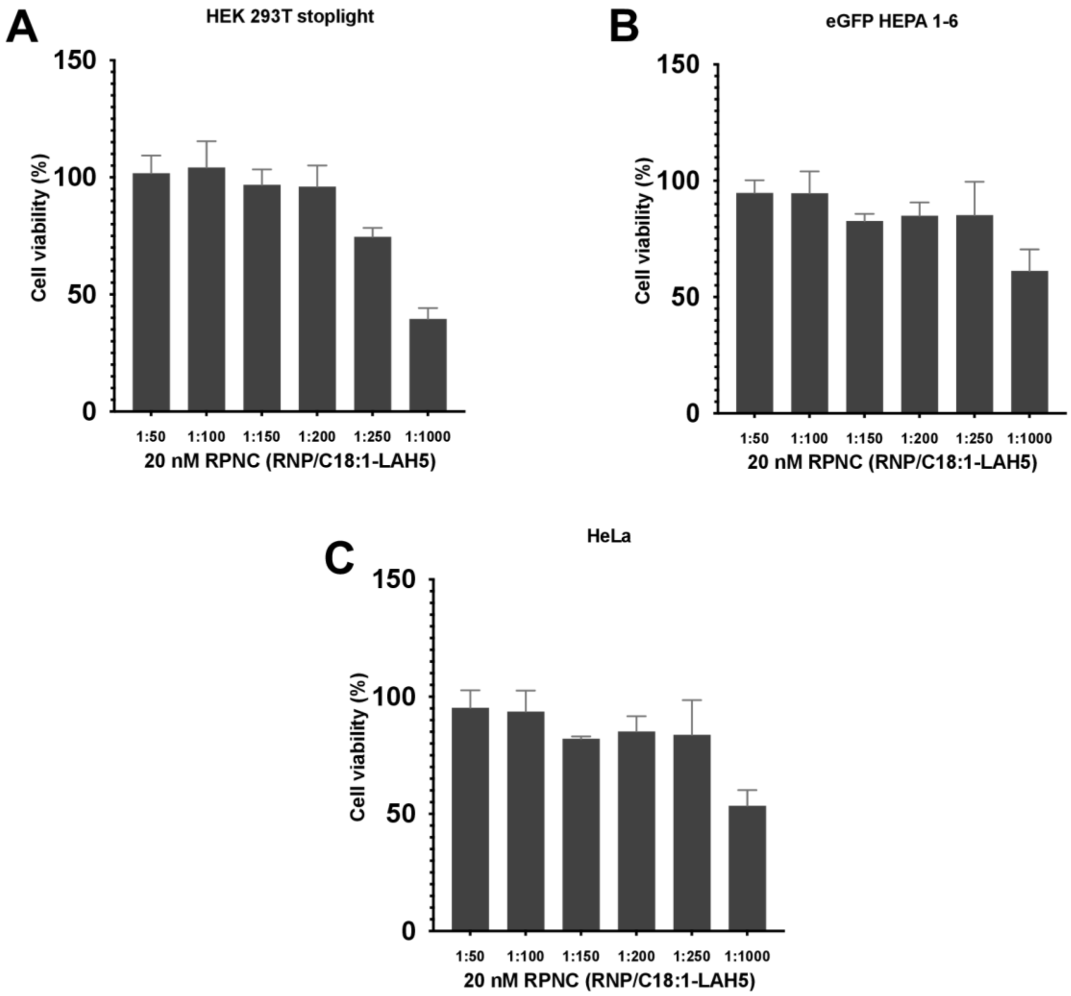

Figure S3. The cytotoxicity assessment of the RPNCs in HEK293T stoplight, eGFP HEPA 1-6, and HeLa cells. A) HEK293T, **B)** eGFP HEPA 1-6 and **C)** HeLa cells were treated with RNP/C18:1-LAH5 peptide (RPNC) at ratios ranging from 1:50 to 1:500, at a fixed concentration of 20 nM. After 24 h incubation, cell viability was tested using MTS assay and the fluorescence intensity was measure with an absorbance set of 490 nm. The normalization process involved excluding the reference positive control signal from both negative control and treated samples. Data shown as the mean ± SD (n=2).

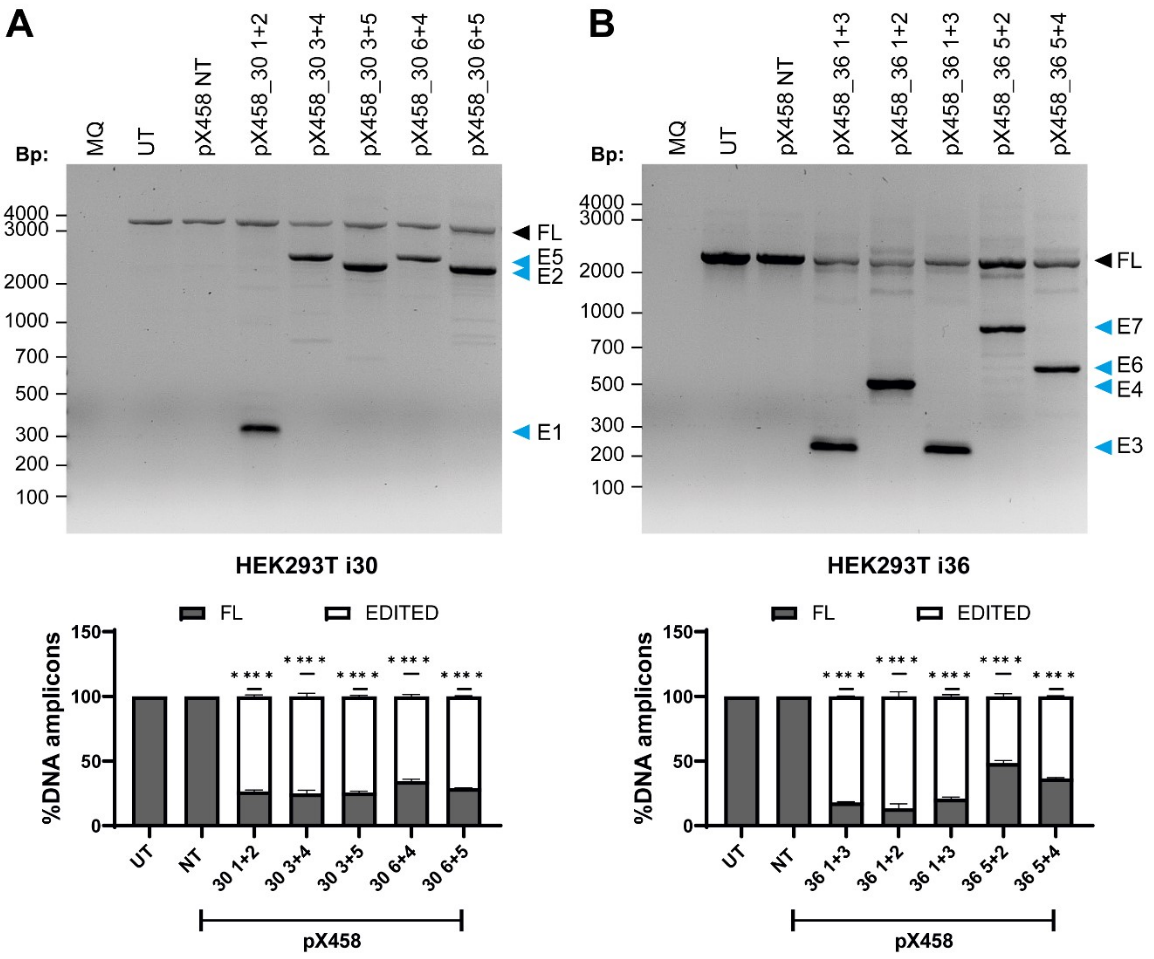

Figure S4. Screening of the efficiency of multiple pairs of gRNAs at genomic DNA level on intron 30 (A) or intron 36 (B) of *ABCA4* gene. On top, a representative electrophoresis gel of the analysis of the gRNAs targeting the intron 30 or intron 36 of *ABCA4.* Each column represents different combinations of transfected pX458 plasmids containing the indicated sgRNAs. These were compared to the empty pX548 vector (pX548 NT) or the untransfected condition (UT) allowing the identification of the expected edited bands, which are highlighted with a blue arrow. a graph chart representing the overall result of the conducted replicates (n=2) indicating the percentage of the full-length amplicon (FL), without editing fragment (EDITED) and the percentage of editing per each condition. Each bar is represented by the mean ± SD (n=2). Statistical significance with respect to the pX458 NT indicated as **** $p < 0.0001$ by one-way ANOVA test followed by Bonferroni correction. Of note, condition pX458_36 1+3 is presented double as same gRNA was named differently, and therefore same condition was performed twice.

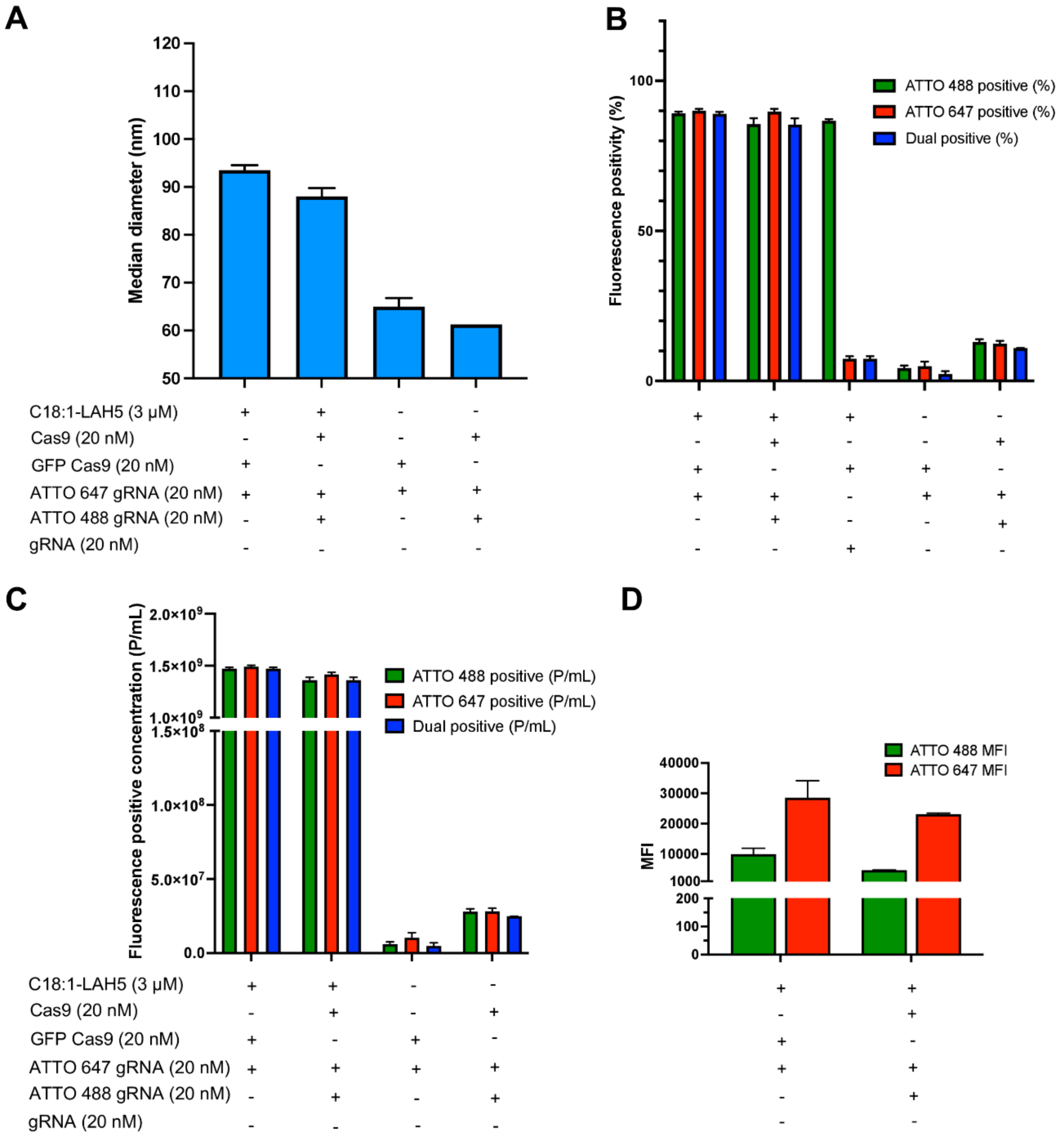

Figure S5. Peptide mediated nanocomplexation (RPNC formation) determined by Nano-Flow Cytometry. A) Median diameter of the RPNCs determined as determined by NanoFCM. The median diameters were consistently higher in the samples (GFP Cas9/ATTO 647 gRNA) (RNP) and samples (Cas9/ ATTO488-gRNA1/ATTO647-gRNA2) (RNP) when they were formulated with the C18:1-LAH5 peptide, as compared to samples that did not contain this peptide. Data shown: mean ± SD. Of note, the experiment was conducted in duplicate for samples containing C18:1-LAH5 in two independent trials, while the control samples without C18:1-LAH5 were conducted in duplicate in a single independent trial. **B)** Cas9/RNP encapsulation efficiency was defined as percentage of fluorescently labeled components positivity (GFP Cas9, ATTO 488 gRNA1, and ATTO647-gRNA2). The presence of the C18:1-LAH5 lipopeptide in the (GFP Cas9/ATTO647-gRNA) and (GFP Cas9/gRNA), as well as in the (Cas9/ ATTO488-gRNA1/ATTO647-gRNA2), resulted in higher levels of fluorescence positivity compared to samples that did not complex with the peptide. Data shown: mean ± SD (n=2). **C)** Cas9/RNP loading efficiency was defined as Fluorescence positive particle concentration (Particles/mL) of fluorescently labeled components (GFP Cas9, ATTO 488 gRNA1, and ATTO 647 gRNA2). The complexation with the C18:1-LAH5 peptide enhanced the concentration of fluorescence-positive particles (P/mL) in both the GFP Cas9/ATTO647-gRNA (RNP) and the Cas9/ATTO488-gRNA1/ATTO647-gRNA2 (RNP) RPNCs. **D)** MFI of RPNCs for gene editing and PIR. Data shown as the mean ± SD. The experiment was conducted in duplicate for samples containing C18:1-LAH5 in two independent trials, while the control samples without C18:1-LAH5 were conducted in duplicate in a single independent trial.

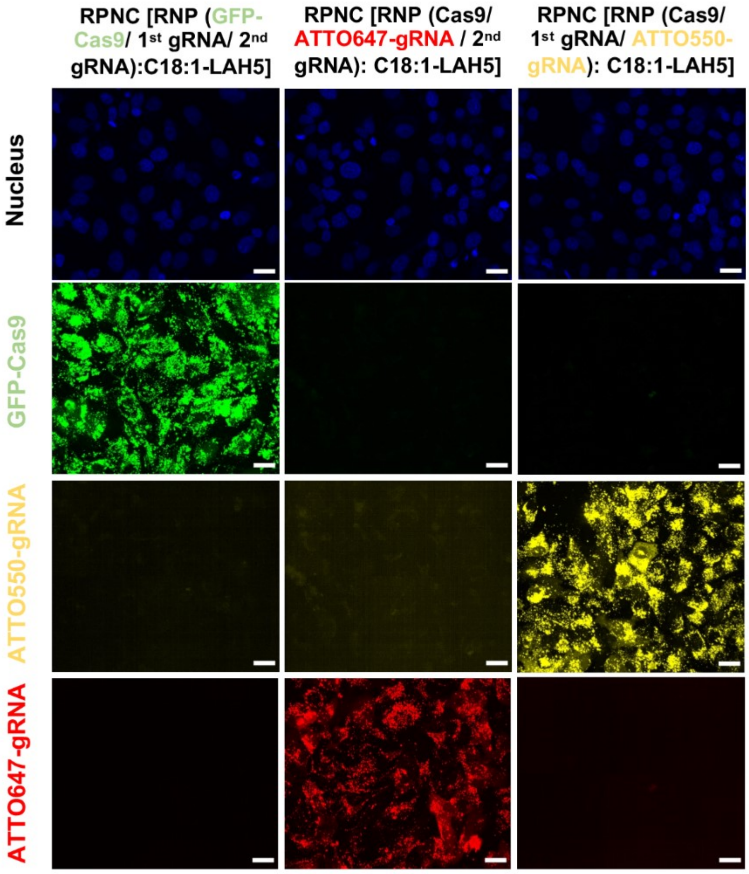

Figure S6. Verification of the simultaneous intracellular uptake of three different fluorescently labeled compounds. To validate the cellular uptake of three distinct fluorescently labeled molecules, HeLa cells were treated with the RPNC including only a single labeled component. Each individual component was combined in different combinations with GFP-Cas9/gRNA1/gRNA2, non-labeled Cas9/ATTO647-gRNA1/gRNA2, and non-labeled Cas9/gRNA1/ATTO550-gRNA2 to form ribonucleoprotein (RNP) complexes. Subsequently, these RNP complexes were combined with the C18:1-LAH5 lipopeptide to form RPNC, after which cells were treated with them. These control images were recorded 24 h after treatment. Scale bars represent 30 µm.

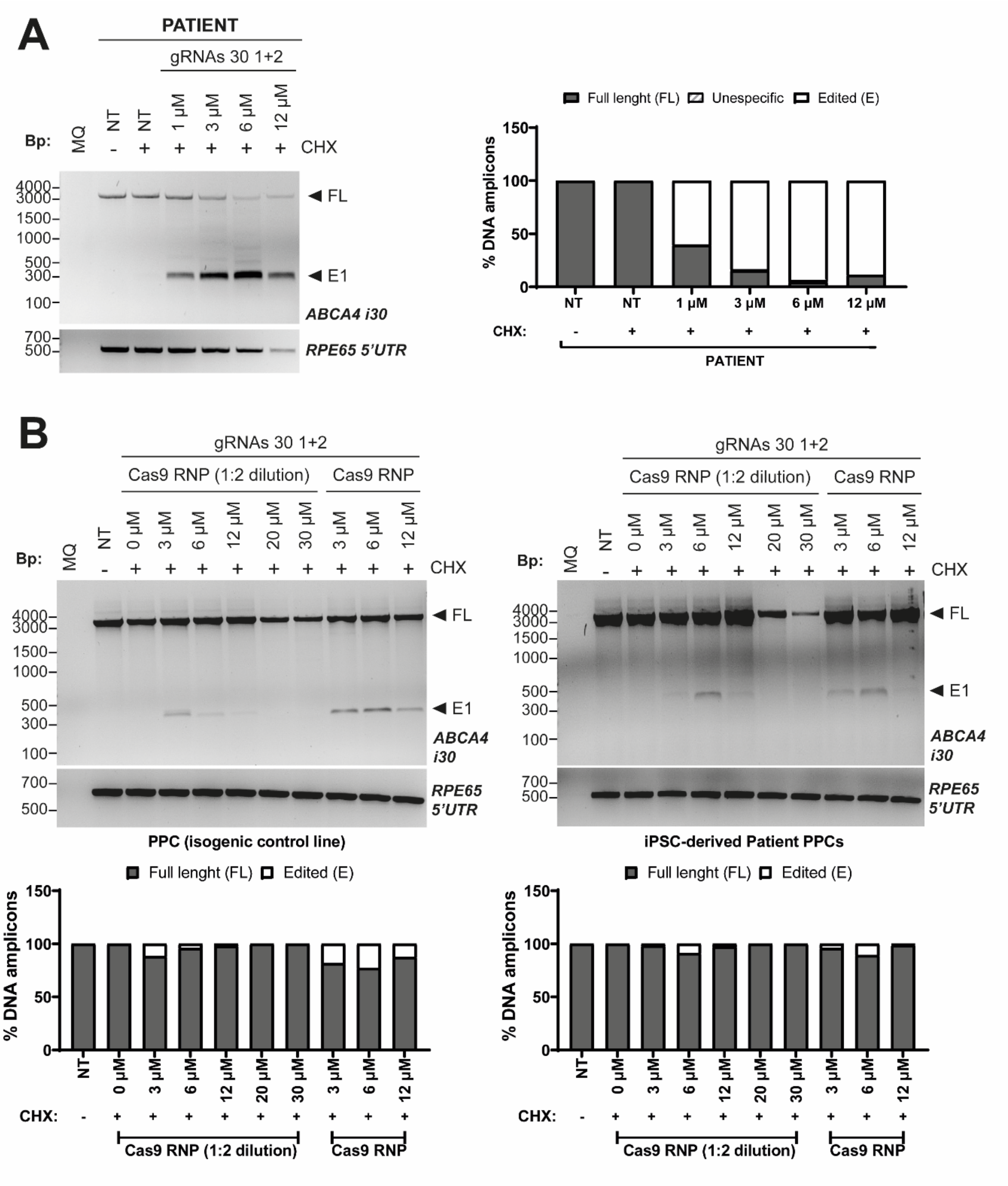

Figure S7. Dose-curve analysis of the RPNCs in fibroblast (A) and photoreceptor precursor cells (B) at genomic DNA level. A) On the left, representative electrophoresis gel of the amplification of intron 30 of *ABCA4* by PCR in both control and patient fibroblast cell lines. Edited band 1 (E1) indicates the expected edited band after treating with RPNC including gRNAs 30-1 and 30-2 after different lipopeptide dose treatment (from 1 – 12 µM). The 5´UTR region of *RPE65* was amplified as loading control. On the right, a graph chart representing the overall result (n=1) indicating the percentage of the full-length amplicon (FL), without editing and the percentage of editing (edited band, E) per each condition. Each bar is represented by median. In addition, some unspecific amplifications that do not correspond to the editing but neither associated with a specific PCR product were also represented. B) On top, representative electrophoresis gels of the amplification of the intron 30 in the isogenic control- (left) or patient-derived PPCs (right) line by PCR. In each, the full-length (FL) amplicon and the expected edited band (E1) are indicated on the right edge of the gel. Each column represents the treatment with the gRNA 30 1+2 together with different dilution of Cas9 RNP and different lipopeptide concentrations (0 – 30 µM). The 5´UTR region of *RPE65* was amplified as loading control. Below, a graph chart representing the overall result indicating the percentage of the full-length transcript (FL) and of the edited band (E) per each condition.

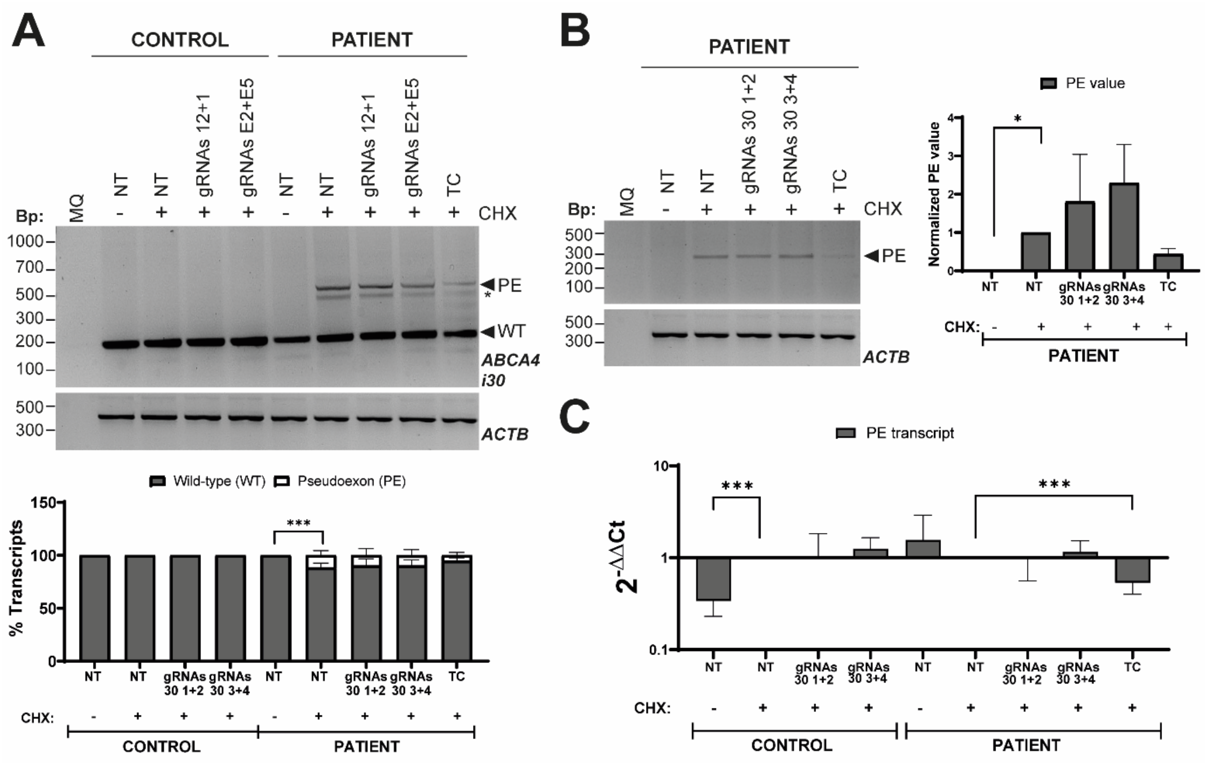

Figure S8. Analysis of the effect of the genome editing of intron 30 of *ABCA4* of fibroblasts at the RNA level of fibroblast. A) RT-PCR from exon 30 to exon 31 of *ABCA4* (n=4). On top, representative electrophoresis gel of the RT-PCR, below the graph chart showing the normalized value of the pseudoexon in comparison with the NT+ condition of control or patient fibroblast. Each bar represents the mean + SD. * means the presence of heteroduplex between the PE transcript and the WT transcript. B) RT-PCR which specific amplifies the pseudoexon (PE) transcript (n=4). On the left a representative electrophoresis gel of the analysis. On the right, a graph chart indicates the normalized value of pseudoexon transcript in comparison with NT+ of each condition of the two conducted replicated. Each bar represents the mean + SD. A and B) *ACTB* was amplified as a loading control. MQ indicates the negative control of the PCR; NT means non-treated cells. The trans-differentiation control (TC) consists of fibroblasts that were seeded and cultured together with the trans-differentiated fibroblasts. However, the TC fibroblasts were cultured without trans-differentiation medium, meaning that they did not undergo trans-differentiation. Therefore, they serve as a negative control for the trans-differentiation process. Statistical significance with respect to the untreated condition (NT +) is indicated as * $p < 0.05$ between NT- and NT+ by one-way ANOVA test followed by Bonferroni correction. This indicates that the CHX treatment worked. However, no statistical difference was observed with respect to the remaining remained conditions. C) qPCR of the pseudoexon (PE) in the control and patient line (n=4). Each condition was normalized against *GUSB* and then compared with the NT+ condition of each cell line. A to C) CHX indicates if cell were treated with (+) or without (-) cycloheximide 24 hours prior to harvesting. Statistical significance with respect to the untreated condition (NT +) is indicated as *** $p < 0.001$ between by T-test.

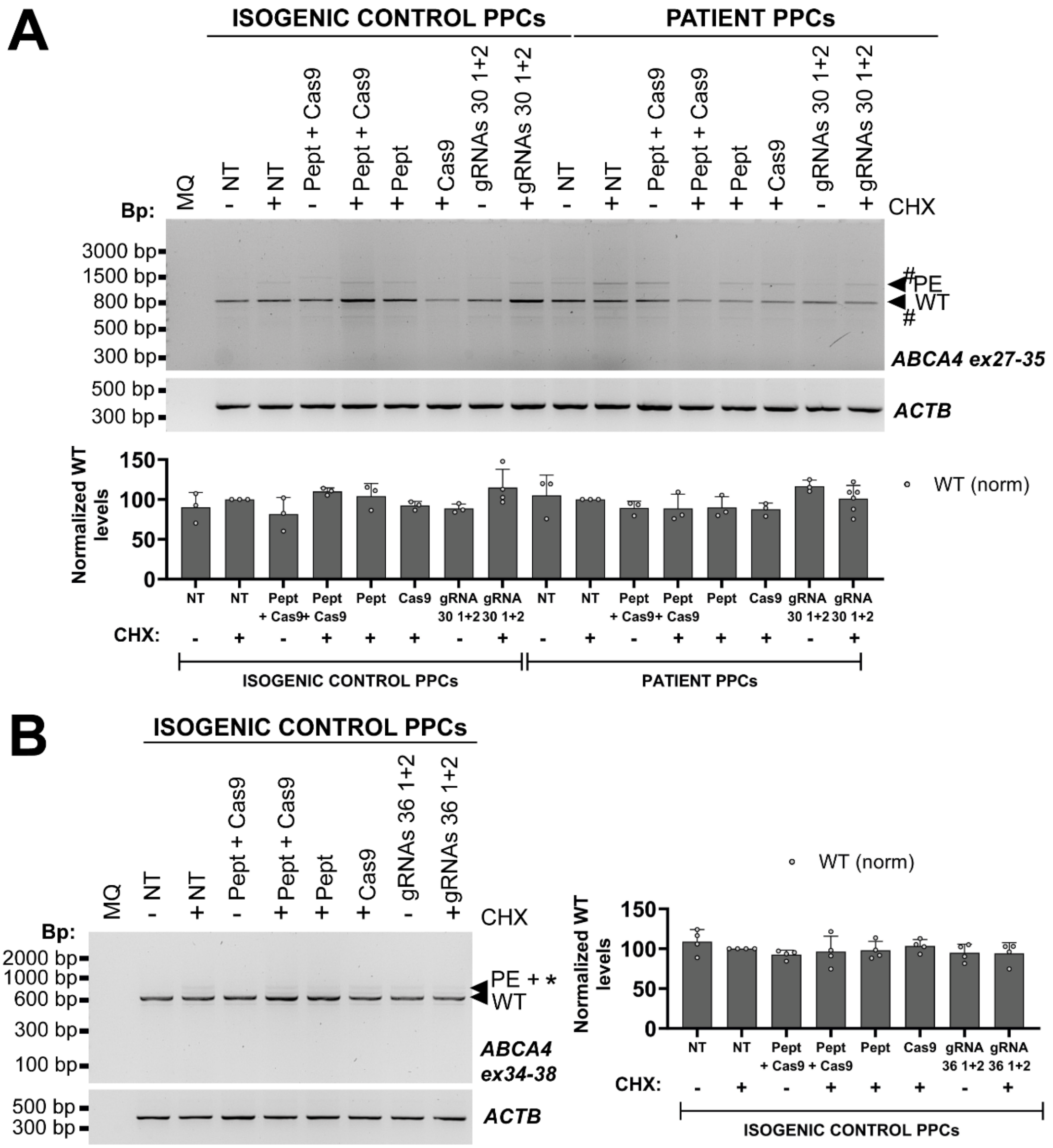

Figure S9. Analysis of the effect of the partial intron removal in the *ABCA4* gene after delivery of RPNC with gRNA30 1+2 or gRNA36 1+3 in photoreceptor precursor cells (PPCs) at RNA level. A) RT-PCR from exon 27 to exon 35 of *ABCA4* (n=3 with exception of samples for gRNAs 1+2 CHX+ whose n=4-6). Upper panel, a representative electrophoresis gel of the RT-PCR is shown. Two main transcripts are visible: wild-type transcript (WT) and the pseudo exon (PE) transcript. # denotes PCR artifacts that we could not assign to any specific transcript, but present in all samples independently of the treatment. Below, the graph chart displays the normalized expression of the amplified region against *ACTB,* which was used as loading control. NT CHX + samples (for each cell line) was set as reference at 100%. Each bar represents the mean + SD, where each light gray dot represents the normalized value obtained in each replicate. B) RT-PCR from exon 34 to exon 38 of *ABCA4* (n=4). On the left, a representative electrophoresis gel of the RT-PCR. Of note, the PE at intron 36 can naturally appear when using control PPC RNA as described previously (Khan et al., 2020[4]), this PE is more apparent after CHX treatment. Asterisk (*) indicates an heteroduplex between the WT and the PE transcripts. On the right, the graph chart showing the normalized expression of the WT transcript against *ACTB*, which was used as loading control. NT CHX + condition was set as reference at 100%. A and B) MQ indicates the negative control of the PCR; NT means non-treated cells. CHX indicates if cell were treated with (+) or without (-) cycloheximide 24 h prior to harvesting. MQ indicates the negative control of the PCR; NT means non-treated cells. CHX indicates if cell were treated with (+) or without (-) cycloheximide 24 h prior to harvesting. No statistical significance was found relative to the corresponding NT condition.

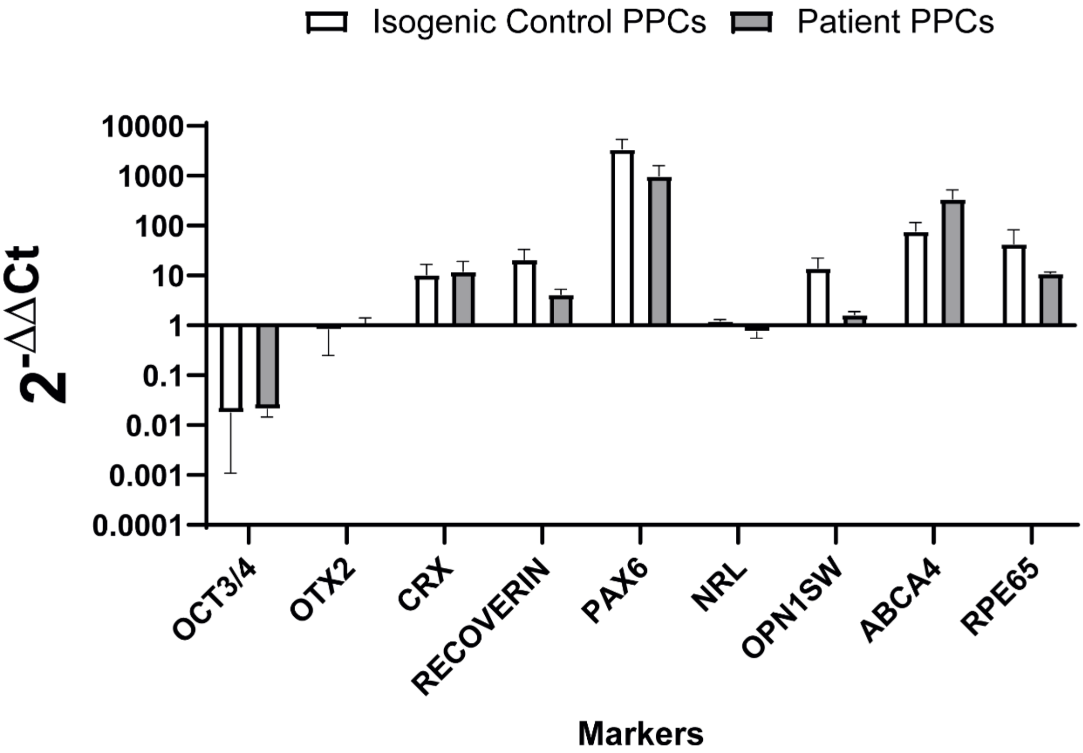

Figure S10. Gene expression profile analysis by qPCR of the photoreceptor progenitor cells (PPCs) differentiation markers in the isogenic control and patient-derived PPCs. Gene expression profile of isogenic control-derived PPC (with) and patient-derived PPCs (in grey) after 30 days of differentiation. The $2^{-\Delta\Delta CT}$ represents the relative expression against *GUSB* and then normalized to the relative value of the iPSC (Day 0 of the differentiation) (n=3). Differentiation into PPCs is observed by the reduced expression of the pluripotent marker *OCT3/4* and an increased expression of photoreceptor markers. Finally, isogenic control and patient-derived PPCs presented an increased expression of *ABCA4*.

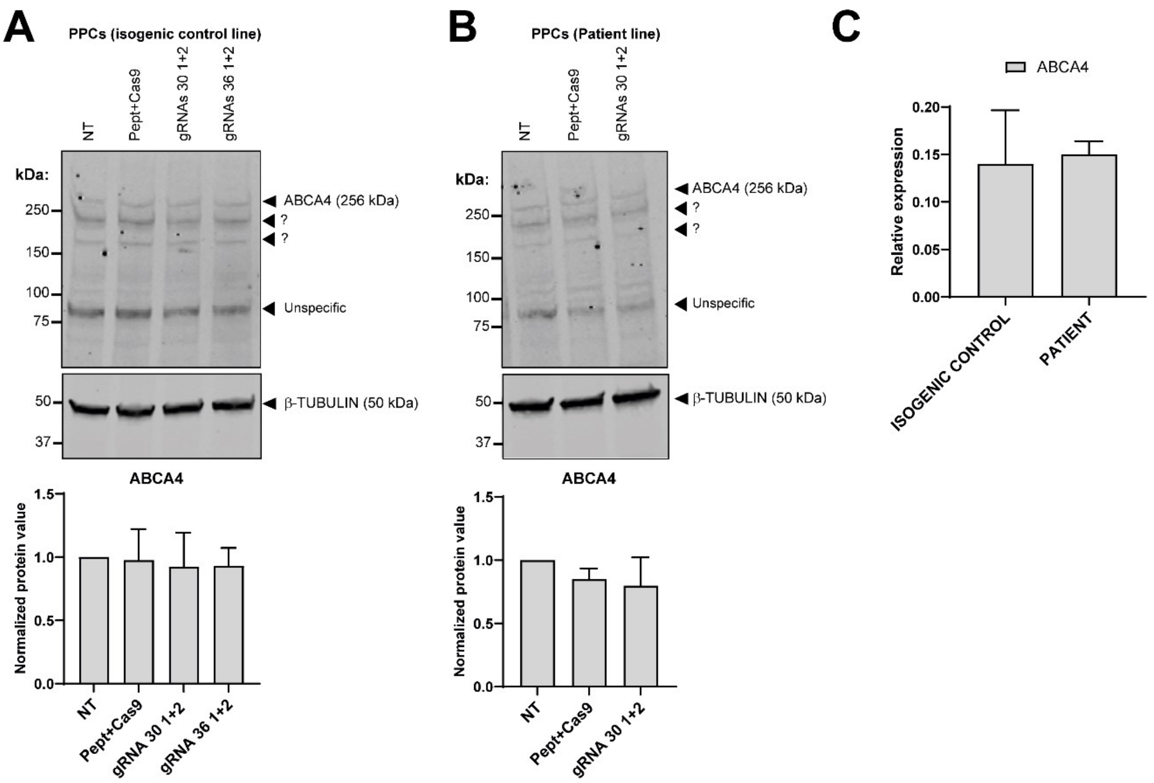

Figure S11. Western blot analysis on PPCs after 12 days of treatment with the RPNC with gRNAs 30-1 and 30-2. On day ~21 of differentiation, RPNCs were delivered to the isogenic control (A) and to patient PPCs (B). A and B) On top a representative western blot for ABCA4 and β-TUBULIN. The upper panel depicts the immunodetection of the ABCA4 protein (256 kDa). In this panel a known unspecific band at 80 kDa was also detected together with some unidentified (?) bands between 150 and 200 kDa. The detection of these bands is very variable, but they have been detected before in other PPC models or in fibroblasts (Suárez-Herrera et al, 2024[5]), so here they seem to be related to the heterogeneity of the PPC model. The lower panel shows β-TUBULIN (50 KDa), which was used as loading control and to normalize ABCA4 levels in each condition. Data (n=2) are represented below as the mean ± SD of the semi-quantification normalized against non-treated PPCs (NT). Pept+Cas9 refers to the cells treated with the RPNC complex but without gRNAs to evaluate possible effects on the RPNC at the protein levels. C) Graph bar representing the mean ± SD values of the relative expression of ABCA4 of the NT conditions in the isogenic control and the patient-derived PPCs. Data (n=2) are normalized only against β-TUBULIN values.

A

				gRNA12			
**Off-Target #**	**Chrom**	**Strand**	**Start**	**End**	**Given query**	**Actual genomic hit**	**# of mismatches**
**1**	10	-	129808349	129808371	TGCTGCCCAGAAGTAGTTGA	gGCTGCCCAGAAGgAcTTGA-GGG	3
**2**	1	-	232832138	232832160	TGCTGCCCAGAAGTAGTTGA	aGCTGCCCAGAAGaAGTgGA-TGG	3
**3**	14	-	58940693	58940715	TGCTGCCCAGAAGTAGTTGA	TcCTGaCCAGAAGTAGTTGc-TGG	3
**4**	11	+	23891308	23891330	TGCTGCCCAGAAGTAGTTGA	TcCTGCtCAGAAGTAGTTcA-GGG	3
**5**	20	-	2658063	2658085	TGCTGCCCAGAAGTAGTTGA	TGtgGCCCAGAAGgAGTTGA-TGG	3
**6**	8	-	105805786	105805808	TGCTGCCCAGAAGTAGTTGA	TGaTGCCCAGAAGTgtTTGA-AGG	3
**7**	20	-	50677454	50677476	TGCTGCCCAGAAGTAGTTGA	TGCTctCCAGAAGTAGTTGg-GGG	3
**8**	4	+	75385660	75385682	TGCTGCCCAGAAGTAGTTGA	TGCTcCCCAGAAGcAGaTGA-TGG	3
**9**	17	+	71552069	71552091	TGCTGCCCAGAAGTAGTTGA	TGCTGgCCAGAAGcAGTgGA-TGG	3

B

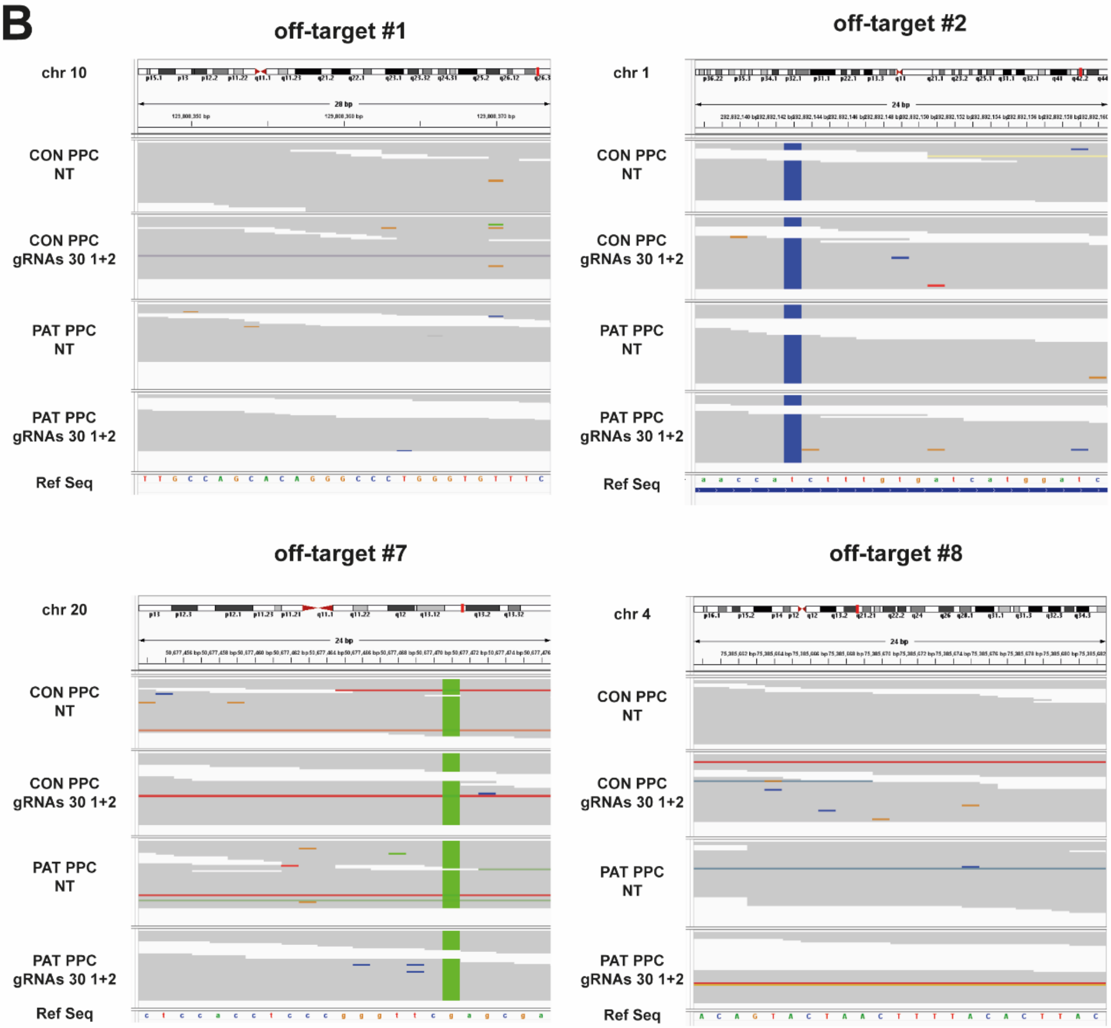

Figure S12. Summary of the analysis of the predicted off-targets for the gRNA 30-1 sequence in control and patient-derived PPCs. A) Table listing the position of the predicted off-targets for gRNA 30-1 indicating the chromosome (chrom), the strand (+ or -), the start and end position, the gRNA sequence (Given query) and the off-target sequence (Actual genomic hit). The mismatches are highlighted in red. The number of mismatches is indicated in the last column. **B)** Representative Integrative Genomics Viewer (IGV) browser screenshots of four off-target sites which showed more discrepancies between non-treated samples (NT) and edited samples. In none of the potential off-target, an effect caused by the gRNA transfection was detected. In all cases, grey color indicated no differences while squares with different color indicated a nucleotide change (C, blue; G, black; A, green and T, red). Some rows are a combination of gray and colored sections rows, indicating a partial overlap of the read with the reference sequence (Ref Seq). CON PPC means isogenic control-derived PPCs. PAT PPC means patient-derived PPCs. In the case off-target #2 and off-target #7 a SNP is emphasized in all samples in blue (T>C) and green (G>A), respectively.

A

gRNA 30-2 (Representative selection)							
Off-Target #	Chrom	Strand	Start	End	Given query	Actual genomic hit	# of mismatches
**1**	7	+	140319829	140319851	CTAAAACTACCAAAAAAATT	CTAAAAaTACCAAAAAAATT-AGG	1
**2**	16	+	17619500	17619522	CTAAAACTACCAAAAAAATT	CTAAAAaTACCAAAAAAATT-AGG	1
**3**	20	+	47511398	47511420	CTAAAACTACCAAAAAAATT	CTAAAAaTACCAAAAAAATT-AGG	1
**4**	3	+	49861718	49861740	CTAAAACTACCAAAAAAATT	CTAAAACTACaAAAAAAATT-AGG	1
**5**	9	+	36196110	36196132	CTAAAACTACCAAAAAAATT	CTAAAACTACaAAAAAAATT-AGG	1
**6**	17	+	36326901	36326923	CTAAAACTACCAAAAAAATT	tTAAAAaTACCAAAAAAATT-AGG	2
**7**	4	-	140694223	140694245	CTAAAACTACCAAAAAAATT	tTAAAACTACCAAAAAAATc-TGG	2
**8**	11	+	8925266	8925288	CTAAAACTACCAAAAAAATT	CaAAAAgTACCAAAAAAATT-AGG	2
**9**	1	+	65100512	65100534	CTAAAACTACCAAAAAAATT	CTAAAAaTACaAAAAAAATT-AGG	2
**10**	8	+	30563853	30563875	CTAAAACTACCAAAAAAATT	CTAAAAaTACaAAAAAAATT-AGG	2
**11**	10	+	14084798	14084820	CTAAAACTACCAAAAAAATT	CTAAAAaTACaAAAAAAATT-AGG	2
**12**	11	+	20818945	20818967	CTAAAACTACCAAAAAAATT	CTAAAAaTACaAAAAAAATT-AGG	2
**13**	14	+	31521767	31521789	CTAAAACTACCAAAAAAATT	CTAAAAaTACaAAAAAAATT-AGG	2
**14**	17	+	49165992	49166014	CTAAAACTACCAAAAAAATT	CTAAAAaTACaAAAAAAATT-AGG	2
**15**	20	+	59611448	59611470	CTAAAACTACCAAAAAAATT	CTAAAAaTACaAAAAAAATT-AGG	2
**16**	1	-	92585337	92585359	CTAAAACTACCAAAAAAATT	CTAAAAaTACaAAAAAAATT-CGG	2
**17**	1	-	113206286	113206308	CTAAAACTACCAAAAAAATT	CTAAAAaTACaAAAAAAATT-AGG	2
**18**	1	-	204466246	204466268	CTAAAACTACCAAAAAAATT	CTAAAAaTACaAAAAAAATT-TGG	2
**19**	8	-	88867494	88867516	CTAAAACTACCAAAAAAATT	CTAAAAaTACaAAAAAAATT-AGG	2
**20**	11	-	32482726	32482748	CTAAAACTACCAAAAAAATT	CTAAAAaTACaAAAAAAATT-AGG	2
**21**	11	-	110069699	110069721	CTAAAACTACCAAAAAAATT	CTAAAAaTACaAAAAAAATT-AGG	2
**22**	14	-	41444071	41444093	CTAAAACTACCAAAAAAATT	CTAAAAaTACaAAAAAAATT-AGG	2
**23**	17	-	31313729	31313751	CTAAAACTACCAAAAAAATT	CTAAAAaTACaAAAAAAATT-AGG	2
**24**	20	-	33253305	33253327	CTAAAACTACCAAAAAAATT	CTAAAAaTACaAAAAAAATT-AGG	2

B

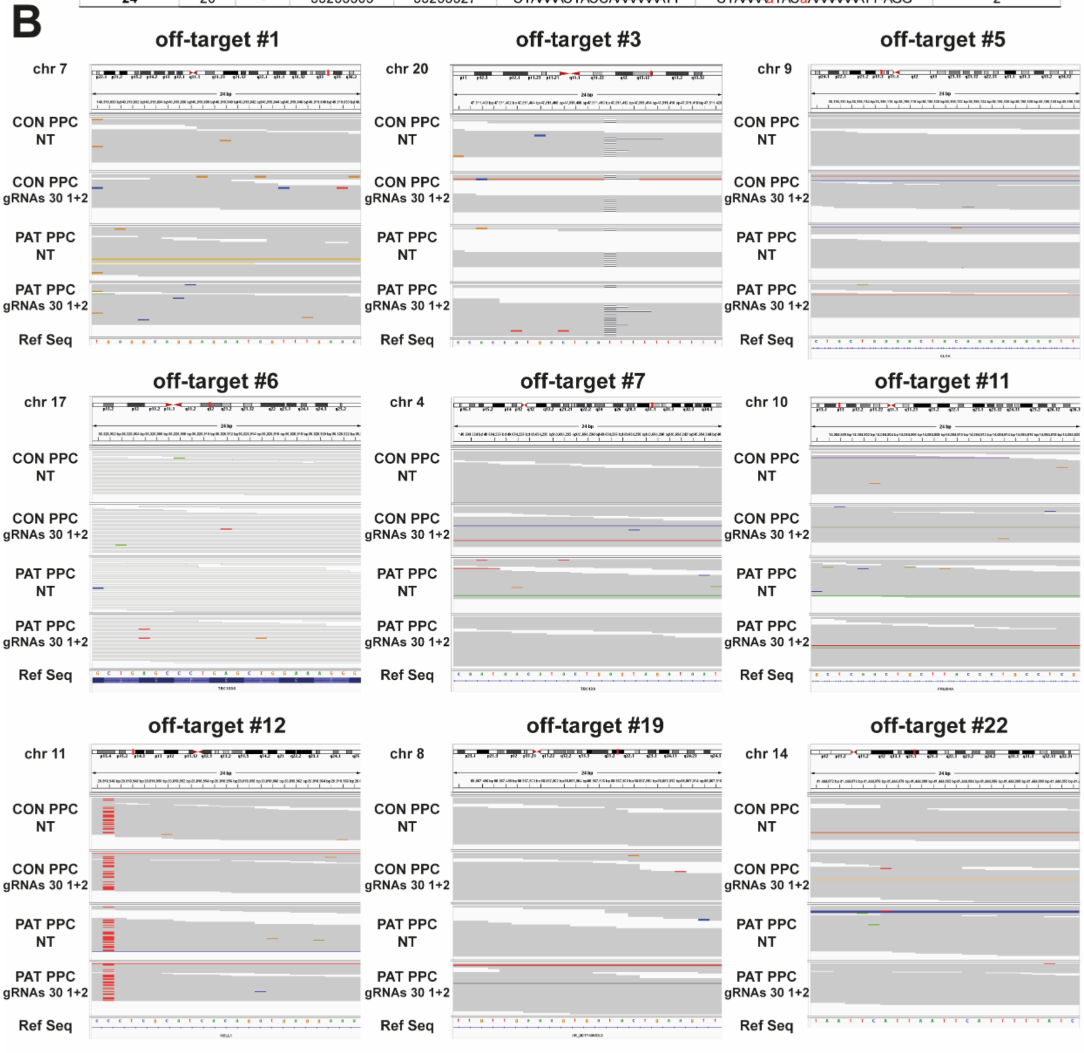

Figure S13. Summary of the analysis of the predicted off-targets for the gRNA 30-2 sequence in control and patient-derived PPCs A) Table listing the position of the predicted off-targets for gRNA 30-2 indicating the chromosome (chrom), the strand (+ or -), the start and end position, the gRNA sequence (Given query) and the off-target sequence (Actual genomic hit). The mismatches are highlighted in red. The number of mismatches is indicated in the last column. **B)** Representative Integrative Genomics Viewer (IGV) browser screenshots of four off-target sites which showed more discrepancies between non-treated samples (NT) and edited samples. In none of the potential off-target, an effect caused by the delivery of the RPNC including gRNAs 30-1 and 30-2was detected. In all cases, grey color indicated no differences while squares with different color indicated a nucleotide change (C, blue; G, black; A, green and T, red). A dark black line indicates a nucleotide missing. Some rows are

a combination of gray and colored sections rows, indicating a partial overlap of the read with the reference sequence (Ref Seq). CON PPC means isogenic control-derived PPCs. PAT PPC means patient-derived PPCs. In the case off-target #12, a SNP heterozygosity is emphasized in all samples in red (C>T).

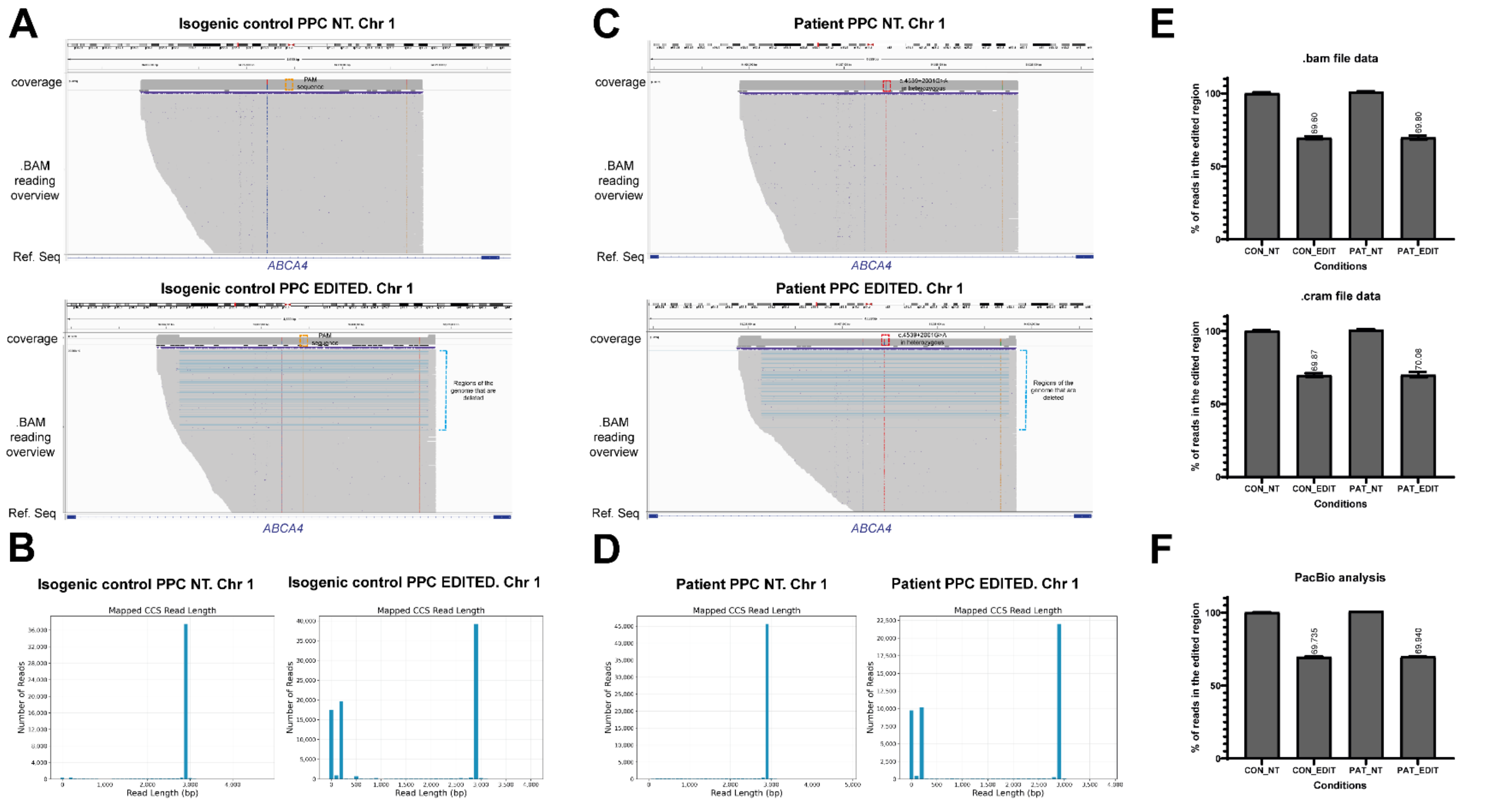

Figure S14. Overview of PacBio data on intron 30 of *ABCA4* (genome editing target) using control and patient-derived PPCs transfected with RPNCs with gRNAs 30-1 and 30-2 A) Overview of the analysis in the isogenic control PPCs using the BAM files. On top the scheme of the non-treated cells (NT) and below for the edited control PPCs. An orange square highlights the PAM sequence resulted after generating the isogenic control are shown. The blue lines represent deleted regions as a consequence of genome editing. **B)** Overview of the number of reads/fragment size of the NT (left) and EDITED (right) isogenic control PPCs **C)** Visual representation of the annotated amplicons in the patient-derived PPCs using the view of the BAM files in IGV. On top the scheme of the non-treated cells (NT) and below for the edited patient-derived PPCs are shown. A red square highlights the c.4539+2001G>A variant in heterozygosis responsible for the splicing defect. The blue lines represent deleted regions as a consequence of genome editing. **D)** Overview of the number of reads/fragment size of the NT (left) and EDITED (right) patient PPCs **E)** Graph bars of the percentage of reads in the predicted edited region by employing the ".BAM" (left) or ".CRAM" (right) files. In all cases the edited samples present a 30% reduction of the reads, corresponding to deleted reads after Cas9-RNP editing. **F**) Graph bar recapitulating the average editing taking into account the results of the ".BAM" and ".CRAM" analysis. The final editing efficiency is around 30% in both isogenic control and patient-derived PPC.

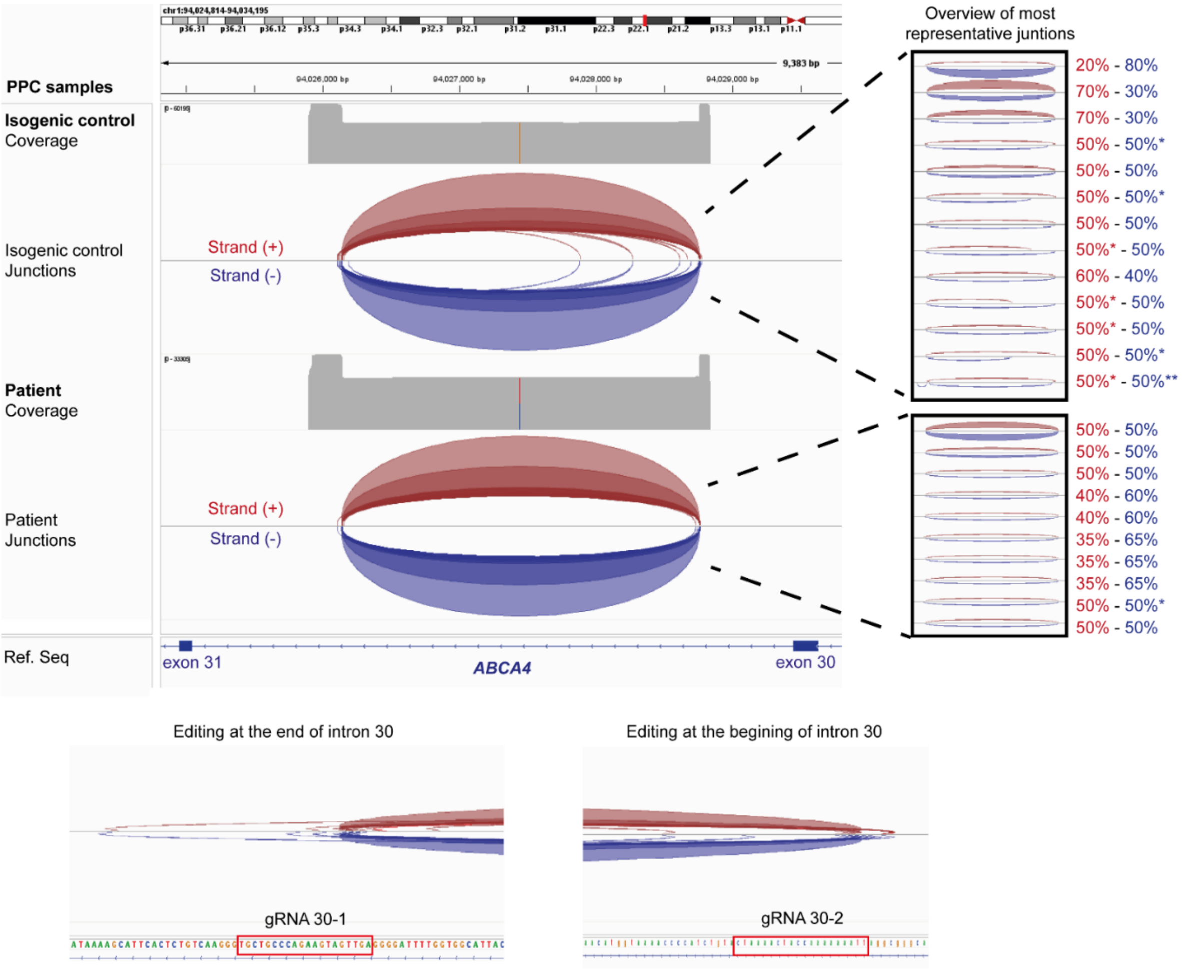

Figure S15. Overview of junctions in the isogenic control and patient PPCs. A summary of the most prevalent juctions is represented in this figure. On the right, there is a representation of different junction pattern detected in each dataset indicating the percentage of editing in the + strand (in red) and the - strand (in blue). * refers to the fact that the ending is upstream or downstream than the most prevalent cut. ** refers to double cut in that region. Ref. Seq shows the reference sequence of the reads showed on top. At the bottom of the figures is represented the end sites of the editing area highlighting the position of the gRNAs used for the PPC.

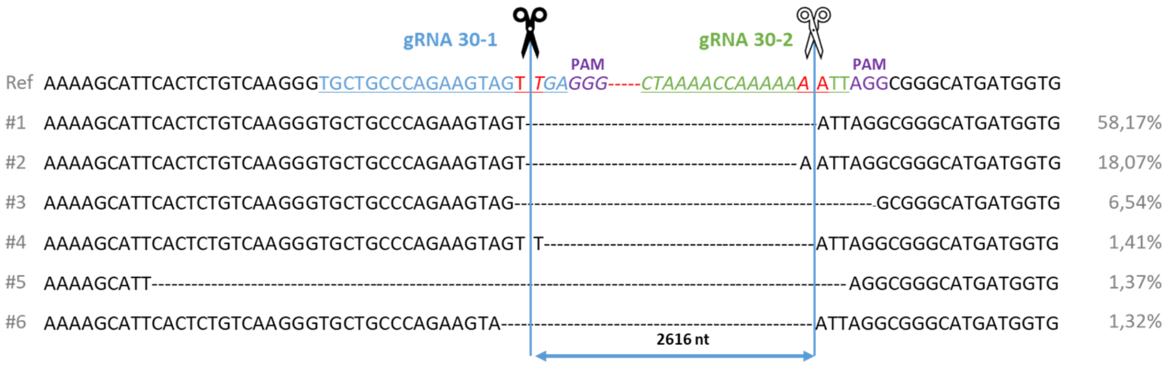

Figure S16. Overview of the repair profile after RPNC-mediated genome editing in intron 30 of *ABCA4*. This figure illustrates the six most frequent double-edited amplicons detected in both isogenic control and patient PPC lines following editing with RPNC using two specific gRNAs: gRNA 30-1 (highlighted in blue with its cutting site marked by black scissors) and gRNA 30-2 (highlighted in green with its cutting site marked by white scissors). The percentage of reads for each sequence from the pool of double-edited sequences combining both cell lines is depicted in grey on the right. The cutting positions of each gRNA on the reference sequence are highlighted in red at the top. PAM sites are highlighted in purple in the reference sequence. The top three sequences collectively accounted for approximately 82% of all reads and were consistently present in both control and patient-edited cells, showing nearly identical ratios. All detected events and number of reads for each line separately, are provided in Supplementary Table S5.

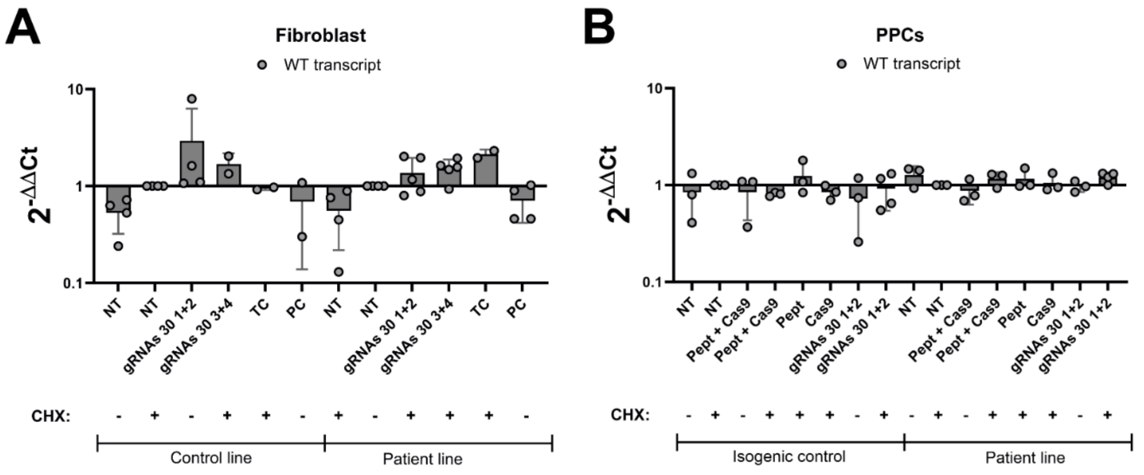

Figure S17. qPCR of the wild-type transcript after delivering the RPNC complex for intron 30 editing to fibroblast (A) or PPCs (B). Data are shown as mean ± SD of n=4 or n=3 replicates for fibroblast (A) and PPCs (B), respectively. Single data points are shown. In none of the cases, a statistical significance is detected between any of the conditions. The list of primers is indicated in Table S6.

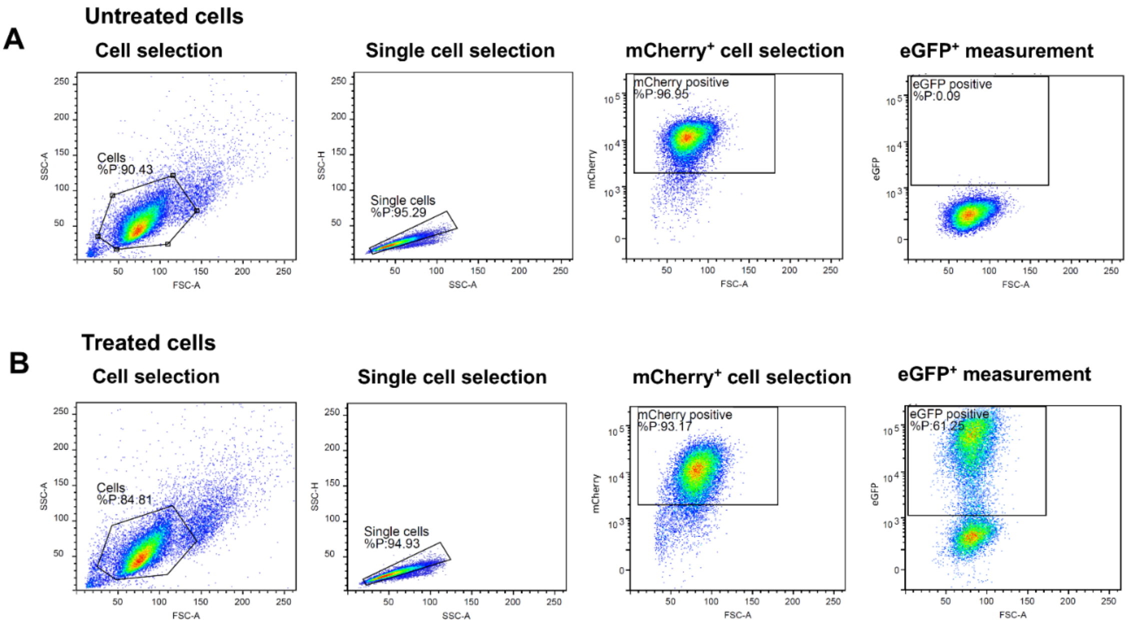

Figure S18 Flow cytometry gating strategy utilized for the assessment of gene editing in HEK293T Stoplight cells. Initially, cells were gated based on forward scatter area (FSC-A) and side scatter area (SSC-A). Subsequently, the isolation of individual cells was achieved by applying selection criteria using SSC-A and side scatter height (SSC-H) signals. Following this, the identification and measurement of Stoplight+ reporter cells was conducted using mCherry signals. Lastly, within the subset of mCherry+ cells, the percentage of eGFP+ cells were assessed. Representative flow cytometry gating strategy is shown for (A) untreated HEK293T Stoplight reporter cells and (B) or HEK293T Stoplight reporter cells treated with RPNCs for gene editing.

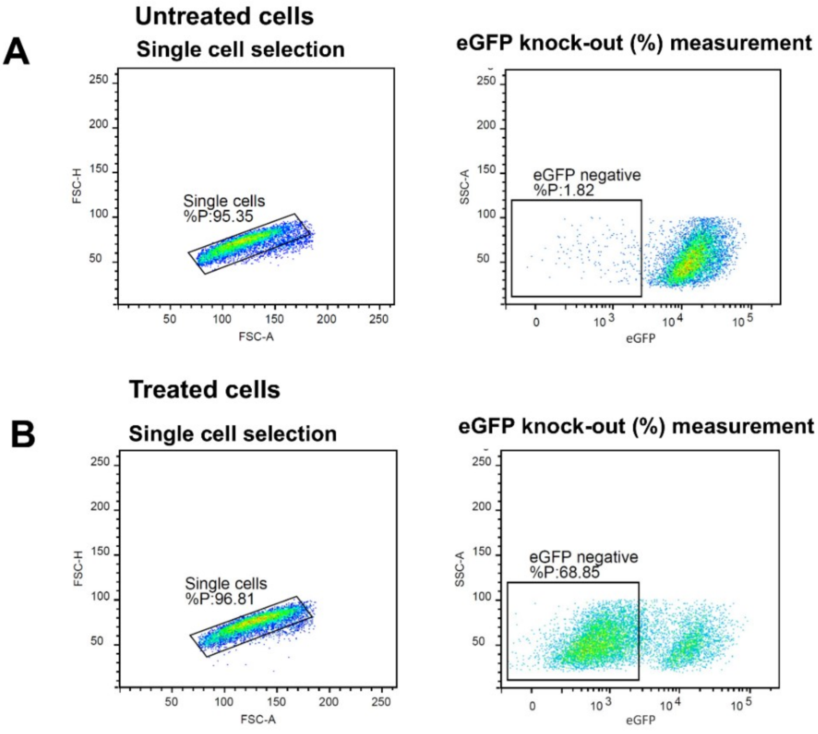

Figure S19. The gating strategy for the analysis of eGFP HEPA 1-6 cells to assess non-edited (eGFP positive), and edited (eGFP negative) cell populations. Percentage of gene editing was calculated by identified eGFP negative cells population. Representative flow cytometry gating strategy is shown for A) non-treated and B) treated with RPNCs for gene editing.

Supplemental tables.

Table S1. Summary of deep-intronic (DI) variants in intron 30 and 36 of *ABCA4*.

Chromosomal position (hg19)	DNA variant	Intron	Severity*	Number of alleles	RNA variant	Predicted protein variant	Reference
1:g.94493901T>C	c.4539+1100A>G	30	Moderate	5	r.[4539_4540ins4539+1033_4539+1100,4539_4540ins4539+989_4539+1100,=]	p.[Arg1514Valfs*31,Arg1514Glyfs*3,=]	Sangermano et al. 2019[6]
1:g.94493895G>A	c.4539+1106C>T	30	Severe	5	r.[4539_4540ins4539+1033_4539+1100,4539_4540ins4539+989_4539+1100]	p.[Arg1514Glyfs*3,Arg1514Valfs*31]	Sangermano et al. 2019[6] Bauwens et al. 2019[7]
1:g.94493037C>A	c.4539+1964G>T	30	Severe	2	r.[4539_4540ins4539+1891_4539+1962]	p.(Gln1513_Arg1514ins(24))	Corradi et al. 2023[8]
1:g.94493000C>T	c.4539+2001G>A	30	Severe	84(┼)	r.[=,4539_4540ins4539+1891_4540-2162]	p.[=,Arg1514Leufs*36]	Braun et al. 2013[9] Bax et al. 2015[10] Albert et al. 2018[11]
1:g.94492973G>A	c.4539+2028C>T	30	Moderate	38	r.[=,4539_4540ins4539+1891_4540-2162]	p.[=,Arg1514Leufs*36]	Braun et al. 2013[9] Bax et al. 2015[10] Albert et al. 2018[11]
1:g.94492937G>A	c.4539+2064C>T	30	Mild	50	r.[=,4539_4540ins4539+1891_4540-2162]	p.[=,Arg1514Leufs*36]	Bauwens et al. 2019[7]
1:g.94492936G>C	c.4539+2065C>G	30	Mild	3	r.[4539_4540ins4539+1891_4539+2060,=]	p.[Arg1514Lysfs*35,=]	Khan et al. 2019[12]
1:g.94492935G>C	c.4539+2066C>G	30	Mild	1	r.[=,4539_4540ins4539+1891_4539+2065,[4254_4352del,4539_4540ins4539+1891_4539+2065],4254_4352del]	p.[=,Gln1513_Arg1514ins*6,[Ser1418_Pro1451delinsArg,Arg1514Ser*5],Ser1418_Pro1451delinsArg]	Jespersgaard et al. 2019[13]
1:g.94490612A>T	c.4540-8T>A	30	Severe	1	r.4539_4540ins4540-6_4540-1	p.Gln1513insProGln	Khan et al. 2020[14]
1:g.94485132_94485135del	c.5196+3_5196+6del	36	Severe	6	r.4849_5196del	p.Val1617_Ile1732del	Sangermano et al. 2018[15]

1:g.94484125T>C	c.5196+1013A>G	36	Severe	1	r.5196_5197ins5196+880_5196+1008	p.Met1733Valfs*2	Khan et al. 2020[4]
1:g.94484082T>C	c.5196+1056A>G	36	Severe	34	r.5196_5197ins5196+880_5196+1056	p.Met1733Valfs*2	Braun et al. 2013[9] Khan et al. 2020[4]
1:g.94484004G>C	c.5196+1134C>G	36	Moderate	1	r.[=,5196_5197ins5196+1135_5196+1212]	p.[=,Met1733Tyrfs*7]	De Angeli et al. 2023
1:g.94484001C>T	c.5196+1137G>A	36	Severe	136	r.[=,5196_5197ins5196+1140_5196+1212]	p.[=,Met1733Glufs*78]	Braun et al. 2013[9] Khan et al. 2020[4]
1:g.94483922G>T	c.5196+1216C>A	36	Moderate	4	r.[=,5196_5197ins5196+1140_5196+1212]	p.[=,Met1733Glufs*78]	Braun et al. 2013[9] Khan et al. 2020[4]
1:g.94481967C>A	c.5197-557G>T	36	Severe	1	r.5196_5197ins5197-563_5197-750	p.Met1733*	Bauwens et al. 2019[7]

Listed are variants that have been reported as pathogenic and for which induced missplicing has been confirmed either by using midigene assays or iPSC-derived models. Number of alleles reported were obtained from LOVD database for unique variants in ABCA4 (last access on May 6th, 2024) and only variants showing pseudoexon inclusion were included in the list; url: https://databases.lovd.nl/shared/variants/ABCA4/unique. ┼ means that this variants has been reported as founder mutation in the paper of Cremers, Collin and Allikments, 2020.[16] * Classification according to Khan et al. 2020[4] based on WT transcript present in either a midigene system or patient-derived retinal cells.

Table S2. Summary of the gRNAs designed and the result of the initial pair screening in HEK293T.

	Intron 30 upstream of hot-spot region				
**#**	**gRNA**	**Targeting sequence**	**PAM**	**Comments**	**Result of initial screening in HEK293T (Figure S4A)**
1	30-3	GACTTTAGAACTCCCAGGAC	AGG	Does not cover c.4539+859C>T	Selected for further experiments in fibroblasts and PPCs
2	30-6	TTCTGGACTTTAGAACTCCC	AGG	Does not cover c.4539+859C>T	Not selected
3	11	GCACAAGAATTGAACCTGGG	AGG	Discarded too many predicted off-targets	Not tested
**4**	**30-2**	**CTAAAACTACCAAAAAAATT**	**AGG**		Selected for further experiments in fibroblasts and PPCs
5	13	CATCTAAAAGTCTACGTGGG	TGG	Not covering more than 3 variants described in the intron	Not tested
6	14	GCCTCATCAGCCAATAAGGC	AGG	Not covering more than 3 variants described in the intron	Not tested
	Intron 30 downstream of hot-spot region				
**#**	**gRNA**	**Targeting sequence**	**PAM**	**Comments**	**Result of initial screening in HEK293T (Figure S4A)**
7	30-4	TCTAAGCTGGACAGACCGCG	TGG	Not covering more than 3 variants described in the intron	Selected for further experiments in fibroblasts
8	30-5	ACAGCTCCTGTGCATATGCG	TGG	Does not cover c.4540-2036C>A	Not selected for further experiments
9	E10	AGAATGGTGCGCAATAACGG	CGG	gRNA discarded. TIDE test did not detect the gRNA sequence	Not tested
**10**	**30-1**	**TGCTGCCCAGAAGTAGTTGA**	**GGG**	Selected for further experiments	Selected for further experiments in fibroblasts and PPCs
11	2	GGTGCATGGGTTTGTCCACC	AGG	Cloning on pX458 failed.	Not tested
12	4	GTTAGCTGACTTGCACTGAC	TGG	Cloning on pX458 failed.	Not tested
	Intron 36 uptream of hot-spot region				
**#**	**gRNA**	**Targeting sequence**	**PAM**	**Comments**	**Result of initial screening in HEK293T (Figure S4B)**
**13**	**36-1**	**TCAAATAATCACTGCACCGC**	**AGG**		Selected for further experiments in fibroblasts and PPCs
14	36-5	TTAGATTAACCACCGCTACA	GGG		Not selected for further experiments
15	15	ATTAGATTAACCACCGCTAC	AGG	(overlap with sequence 36-5). Discarded.	Not tested
	Intron 36 downstream of hot-spot region				
**#**	**gRNA**	**Targeting sequence**	**PAM**	**Comments**	**Result of initial screening in HEK293T (Figure S4B)**
16	36-3	TCCAAAATGATGGATCGCGG	AGG		Selected for further experiments in fibroblast
17	36-4	GTATACATCGGACGTGCTGA	GGG		Not selected for further experiments
**18**	**36-2**	**TATACATCGGACGTGCTGAG**	**GGG**		Selected for further experiments in fibroblasts and PPCs

tracrRNA sequence is property of IDT and it cannot be disclosured. The one employed in this work was purchased by the reference 1072532 (Alt-R® CRISPR-Cas9 tracrRNA; IDT).

Table S3. MFI values of RPNCs used for Figure S5D.

Sample	eGFP MFI (AU)				647 MFI (AU)			
	N1	N2	Mean	SD	N1	N2	Mean	SD
GFP Cas9/ATTO647 gRNA/C18:1-LAH5 replicate 1	6503,3	6838,5	6670,9	167,6	20713,5	23101,6	21907,6	1194,0
GFP Cas9/ATTO647 gRNA/C18:1-LAH5 replicate 2	8570,3	11314,6	9942,4	1372,1	24585,6	32518,2	28551,9	3966,3
Cas9/ ATTO488 gRNA1/ATTO647 gRNA2/C18:1-LAH5 replicate 1	3908,1	2921,0	3414,6	493,6	16828,6	12789,2	14808,9	2019,7
Cas9/ ATTO488 gRNA1/ATTO647 gRNA2/C18:1-LAH5 replicate 2	4700,3	4622,2	4661,3	39,0	22847,9	23344,7	23096,3	248,4

Table S4. Off-target list. Sheet 1 includes the complete list of predicted off-target regions associated with the gRNA gRNA 30-1. Those candidates with more than 3 mismatches are indicated in brown. Sheet 2 includes the complete list of predicted off-target regions associated with the gRNA 30-2. Those candidates with more than 3 mismatches are indicated in brown. Sheet 3 included the final selected list of off-targets for both gRNA.

Table S5. Overview of the 44 repaired sequences identified in PPCs after editing intron 30 with RPNC. All repair events identified in the double edited control and patient samples using PacBio long-read sequencing. The table includes sequences upstream and downstream of the cutting site, along with any insertions present. Columns detail the number of reads containing each mutation across both PPC lines (COMBINED) and their respective percentages (COMBINED %), as well as the number of reads and corresponding frequency in percentage in each line: isogenic control (CONTROL) and patient-derived PPCs (PATIENT). In red the nucleotides in between which the editing is expected. In italics the intronic regions of the reference. In blue substitutions found that could or could not be directly related to the editing. The PAM is depicted in purple.

Table S6. Disclosure of the sequences employed in the C18:1-LAH5 lipopeptide characterization.

**gRNA sequences employed in reporter and characterization experiments**	
**Target**	**gRNA targeting sequence**
Stoplight construct	GGACAGTACTCCGCTCGAGT
eGFP construct	GCTGAAGCACTGCACGCCGT
CCR5	TGACATCAATTATTATACAT
Non-target gRNA	GTTAATGTGGCTCTGGTTCT
ATTO550-gRNA	CCTGACAATCGATAGGTACC
ATTO647-gRNA	CTGTCCATAATTAGTCCATG
ATTO488-gRNA	TCAGTCTATACCCGATCCAC
**PCR primers for the amplification of the *CCR5* locus in the T7E1 assay**	
Primer name	Sequence 5'- 3'
CCR5 Forward	CAACAGAGCCAAGCTCTCCAT
CCR5 Reverse	CCTGGGAGAGACGCAAACAC

Table S7. **List of primers used in the genome editing of *ABCA4* intron 30 and 36.** Underlined sequence on the Site-directed primers represents the binding region complementary to the DNA strand while the non-underlined sequence is a tail complementary to the binding region of the other primer.

Primers for site-directed mutagenesis on the in-house midigene

ABCA4 region	Sequence (5'-3')	Length (bp)	Tm (°C)	%GC	Amplicon size
Intron 30_SDM_1	CATCCATGTCCGATGAACTAGCTTCCAGTCCT	32	64.4	50.0	1680
Intron 30_SDM1	TAGTTCATCGGACATGGATGCATGTTCCTAG	32	61.7	45.0	

Primers used to amplify the gRNA sequences for cloning in the pX548 plasmid

gRNA	Direction	Sequence (5'-3')	Length (bp)	Tm (°C)	%GC	Amplicon (bp)
gRNA 30-1	Forward	GAAAGTCAAATGGGAAAACAGG	22	51.1	41.0	253
	Reverse	AGGACTGGAAGCTAGTTCATCG	22	54.8	50.0	
gRNA 30-2	Forward	GAGTCTCTCTCTGTCACCCAGG	22	58.6	59.0	237
	Reverse	TGGTAGGCAGAAGTGGGTAGAT	22	54.8	50.0	
gRNA 30-3	Forward	CTCTCCATGTGCTGTGTCTC	20	53.8	55.0	210
	Reverse	GAGGCCCATTATCAACCACG	20	53.8	55.0	
gRNA 30-4	Forward	GGACGTCAAGGCTGTGATAC	20	53.8	55.0	533
	Reverse	GTGTTTGAGTGGCTAGGGTG	20	53.8	55.0	
gRNA 30-5	Forward	GGACGTCAAGGCTGTGATAC	20	53.8	55.0	533
	Reverse	GTGTTTGAGTGGCTAGGGTG	20	53.8	55.0	
gRNA 30-6	Forward	CTCTCCATGTGCTGTGTCTC	20	53.8	55.0	210
	Reverse	GAGGCCCATTATCAACCACG	20	53.8	55.0	
gRNA 36-1	Forward	ATCTGCTGTCCCTTGACATCTT	22	53.0	45.0	268
	Reverse	ACCTTTACCCTTTTGAAGGTGG	22	53.0	45.0	
gRNA 36-2	Forward	GTAACTTCTCAGCAAGATGCCC	22	54.8	50.0	182
	Reverse	GGTGTGAGGATTAGGTGGTGAT	22	54.8	50.0	
gRNA 36-3	Forward	AATTTACACACTTGGGGCTG	20	49.7	45.0	652
	Reverse	GGTGTGAGGATTAGGTGGTGAT	22	54.8	50.0	
gRNA 36-4	Forward	AATTTACACACTTGGGGCTG	20	49.7	45.0	652
	Reverse	GGTGTGAGGATTAGGTGGTGAT	22	54.8	50.0	
gRNA 36-5	Forward	GGCATCCTAGATTTTTATTTGCC	23	51.7	39.0	225
	Reverse	CTACCAATTCCTTTGACCCTG	21	52.4	48.0	

Primers used for Sanger sequencing the pDONR_pX458 plasmids

Name	Sequence (5'-3')	Length (bp)	Tm (°C)	%GC

SEQ_pX458_U6-Cas9_Fwd1	TCGCGTTAACGCTAGCATGGATCTC	25	59.3	52.0
SEQ_pX458_U6-Cas9_Fwd2	CTCTGACTGACCGCGTTACT	20	53.8	55.0
SEQ_pX458_U6-Cas9_Fwd3	GAGTCCTTCCTGGTGGAAGA	20	53.8	55.0
SEQ_pX458_U6-Cas9_Fwd4	GACATCCTGAGAGTGAACAC	20	51.8	50.0
SEQ_pX458_U6-Cas9_Fwd5	GAGAAGGTGCTGCCCAAGCA	20	60.0	55.9
SEQ_pX458_U6-Cas9_Fwd6	GACATCCAGAAAGCCCAGGT	20	53.8	55.0
SEQ_pX458_U6-Cas9_Fwd7	GGCAGATCACAAAGCACGTG	20	53.8	55.0
SEQ_pX458_U6-Cas9_Fwd8	GACAGCCCCACCGTGGCCTA	20	60.0	70.0
SEQ_pX458_U6-Cas9_Rev1	ACTGCCAAGTAGGAAAGTCCC	21	54.0	52.0
SEQ_pX458_U6-Cas9_Rev2	GTAACATCAGAGATTTTGAGACAC	24		

DNA analysis

Region and gene	Oligo name	Sequence (5’-3’)	Length (bp)	Tm (°C)	%GC	Amplicon (bp)
Intron 30 *ABCA4*	Intron 30_Fwd	CTCCCAGGTTCAATTCTTGTG	21	52.4	48.0	2936
	Intron 30_Rev	GGCATACAGATGACACAGTTG	21	52.4	48.0	
Intron 36 *ABCA4*	Intron 36_Fwd	ATCTGCTGTCCCTTGACATCTT	22	53.0	45.0	1840
	Intron 36_Rev	GGTGTGAGGATTAGGTGGTGAT	22	54.8	50.0	
5’UTR *RPE65*	5’UTR_Fwd	CTGAACACTCTTCTACCTGC	20	51.8	50.0	485
	Intron1_Rev	TCCAAATCTTCTTAAACCAGC	21	48.5	38.0	

RNA analysis

Region and gene	Oligo name	Sequence (5’-3’)	Length (bp)	Tm (°C)	%GC	Amplicon (bp)
ABCA4 PE 30	Intron 30_Fwd	CTCCACTCAAAACCCTCCAG	20	53.8	55	271
	Exon 31_Rev	ATGTTCCTGTCCGTCAGGTC	20	53.8	55	
ACTB	Exon 3_Fwd	ACTGGGACGACATGGAGAAG	20	60.5	55.0	398
	Exon 4_rev	TCTCAGCTGTGGTGGTGAAG	20	60.5	55.0	
ABCA4 exon 30-31	Exon 30_Fwd	AAACATCACCCAGCTGTTCC	20	51.8	50.0	173
	Exon 31_Rev	GAAGTCGGAGATGTTCCTGTC	21	54.4	52.0	
ABCA4 exon 27-35	Exon 27_Fwd	GCTGCTGGTCAAGAGATTCC	20	60.5	55.0	807
	Exon 35_Rev	GCATGCCAGCCTTTGTTATT	20	56.4	45.0	
ABCA4 exon 34-38	Exon 34_Fwd	ACCTGATTTCCTTAAACATCTAGAAA	26	60.1	31.0	608
	Exon 38_Rev	TTGATGCCGATGAACAGATT	20	54.3	40.0	
RHO-exon 5 (midigenes)	Exon 5_Fwd	ATCTGCTGCGGCAAGAAC	18	64.7	55.6	140
	Exon 5_Rev	AGGTGTAGGGGATGGGAGAC	20	64.5	60.0	

Table S8. qPCR primers list. All qPCR reactions were conducted using an annealing temperature of 60°C

Gene	Sequence (5'-3')	Length	GC%	Amplicon size (bp)
ABCA4_wt transcript_F	AGCACCAGGGAGAAGCTC	18	58.0	81
ABCA4_wt transcript_R	GCGCTGTGTTCTCTGGGG	18	61.0	
ABCA4 intron 30 PE_F (fibroblast)	CTCACCATGCTGCCAGAGT	19	58.0	97
ABCA4 intron 30 PE_R (fibroblast)	CCTTGGGTCCTTCTTTTGGG	20	55.0	
ABCA4 intron 30 PE_F (PPCs)	TAGTGTTGGTCCTTGGTCCC	20	59.0	85
ABCA4 intron 30 PE_R (PPCs)	CTTGTAGAATTTCCGTGCTGC	21	58.0	
*PAX6*_qPCR_F	GCTGCAAAGAAATAGAACATCC	22	41.0	111
*PAX6*_qPCR_R	TTGGCTGCTAGTCTTTCTCG	20	50.0	
*CRX*_qPCR_F	CCCCAGTGTGGATCTGATG	19	58.0	116
*CRX*_qPCR_R	CAAACAGTGCCTCCAGCTC	19	58.0	
*RECOVERIN*_qPCR_F	ACACCAAGTTCTCGGAGGAG	20	55.0	108
*RECOVERIN*_qPCR_R	ACTTGGCGTAGATGCTCTGG	20	55.0	
*OPN1SW*_qPCR_F	TTCTTCTCCAAGAGTGCTTGC	21	48.0	97
*OPN1SW*_qPCR_R	CCTTCCCACACACCATCTTC	20	55.0	
*OTX2*_qPCR_F	TATCTTAAGCAACCGCCTTACG	22	45.0	75
*OTX2*_qPCR_R	GGAGGGGTGCAGCAAGTC	18	67.0	
*RPE65*_qPCR_F	TTACTACGCTTGCACAGAGACC	22	50.0	105
*RPE65*_qPCR_R	GCCCCATTGACAGAGACATAG	21	52.0	
*OCT3/4*_qPCR_F	GTTCTTCATTCACTAAGGAAGG	22	41.0	101
*OCT3/4*_qPCR_R	CAAGAGCATCATTGAACTTCAC	22	41.0	
*GUSB*_qPCR_F	AGAGTGGTGCTGAGGATTGG	20	55.0	80
*GUSB*_QPCR_R	CCCTCATGCTCTAGCGTGTC	20	60.0	
*ABCA4*_qPCR_F	CATCCTGTTCCACCACCTCA	20	55.0	113
*ABCA4*_qPCR_R	CTGTGTCCTCCAACATGGCT	20	55.0	

Table S9. List of antibodies used for Western Blot analysis study.

**Primary Antibodies**	**Target protein**	**Produced by**	**Antibody Catalog #**	**Host**	**Diluent**	**WB dilution**
	ABCA4	Abcam	ab72955	Rabbit	2.5% Not-fally blotto Milk	1:1000
	β-TUBULIN	Abcam	ab15568	Rabbit		1:1000
**Secondary antibodies**	**Target host**	**Produced by**	**Antibody Catalog #**	**Host/ Fluorophore**	**Diluent**	**WB dilution**
	Rabbit	Molecular Probes	A21076	Goat/Alex Flulor 680	2.5% Not-fally blotto Milk	1:10000
	Rabbit	Li-COR biosciences	LI 926-32211	Goat/IRDye800		1:10000

References:

1. de Jong, OG, Murphy, DE, Mager, I, Willms, E, Garcia-Guerra, A, Gitz-Francois, JJ, Lefferts, J, Gupta, D, Steenbeek, SC, van Rheenen, J*, et al.* (2020). A CRISPR-Cas9-based reporter system for single-cell detection of extracellular vesicle-mediated functional transfer of RNA. *Nat Commun* **11**: 1113.
2. Haer-Wigman, L, den Ouden, A, van Genderen, MM, Kroes, HY, Verheij, J, Smailhodzic, D, Hoekstra, AS, Vijzelaar, R, Blom, J, Derks, R*, et al.* (2022). Diagnostic analysis of the highly complex OPN1LW/OPN1MW gene cluster using long-read sequencing and MLPA. *NPJ Genom Med* **7**: 65.
3. Robinson, JT, Thorvaldsdottir, H, Winckler, W, Guttman, M, Lander, ES, Getz, G, and Mesirov, JP (2011). Integrative genomics viewer. *Nat Biotechnol* **29**: 24-26.
4. Khan, M, Arno, G, Fakin, A, Parfitt, DA, Dhooge, PPA, Albert, S, Bax, NM, Duijkers, L, Niblock, M, Hau, KL*, et al.* (2020). Detailed Phenotyping and Therapeutic Strategies for Intronic ABCA4 Variants in Stargardt Disease. *Mol Ther Nucleic Acids* **21**: 412-427.
5. Suarez-Herrera, N, Li, CHZ, Leijsten, N, Karjosukarso, DW, Corradi, Z, Bukkems, F, Duijkers, L, Cremers, FPM, Hoyng, CB, Garanto, A, and Collin, RWJ (2024). Preclinical Development of Antisense Oligonucleotides to Rescue Aberrant Splicing Caused by an Ultrarare ABCA4 Variant in a Child with Early-Onset Stargardt Disease. *Cells* **13**.
6. Sangermano, R, Garanto, A, Khan, M, Runhart, EH, Bauwens, M, Bax, NM, van den Born, LI, Khan, MI, Cornelis, SS, Verheij, J*, et al.* (2019). Deep-intronic ABCA4 variants explain missing heritability in Stargardt disease and allow correction of splice defects by antisense oligonucleotides. *Genet Med*.
7. Bauwens, M, Garanto, A, Sangermano, R, Naessens, S, Weisschuh, N, De Zaeytijd, J, Khan, M, Sadler, F, Balikova, I, Van Cauwenbergh, C*, et al.* (2019). ABCA4-associated disease as a model for missing heritability in autosomal recessive disorders: novel noncoding splice, cis-regulatory, structural, and recurrent hypomorphic variants. *Genet Med*.
8. Corradi, Z, Khan, M, Hitti-Malin, R, Mishra, K, Whelan, L, Cornelis, SS, Group, AB-S, Hoyng, CB, Kampjarvi, K, Klaver, CCW*, et al.* (2023). Targeted sequencing and in vitro splice assays shed light on ABCA4-associated retinopathies missing heritability. *HGG Adv* **4**: 100237.

9. Braun, TA, Mullins, RF, Wagner, AH, Andorf, JL, Johnston, RM, Bakall, BB, Deluca, AP, Fishman, GA, Lam, BL, Weleber, RG*, et al.* (2013). Non-exomic and synonymous variants in ABCA4 are an important cause of Stargardt disease. *Hum Mol Genet* **22**: 5136-5145.

10. Bax, NM, Sangermano, R, Roosing, S, Thiadens, AA, Hoefsloot, LH, van den Born, LI, Phan, M, Klevering, BJ, Westeneng-van Haaften, C, Braun, TA*, et al.* (2015). Heterozygous deep-intronic variants and deletions in ABCA4 in persons with retinal dystrophies and one exonic ABCA4 variant. *Hum Mutat* **36**: 43-47.

11. Albert, S, Garanto, A, Sangermano, R, Khan, M, Bax, NM, Hoyng, CB, Zernant, J, Lee, W, Allikmets, R, Collin, RWJ, and Cremers, FPM (2018). Identification and Rescue of Splice Defects Caused by Two Neighboring Deep-Intronic ABCA4 Mutations Underlying Stargardt Disease. *Am J Hum Genet* **102**: 517-527.

12. Khan, M, Cornelis, SS, Khan, MI, Elmelik, D, Manders, E, Bakker, S, Derks, R, Neveling, K, van de Vorst, M, Gilissen, C*, et al.* (2019). Cost-effective molecular inversion probe-based ABCA4 sequencing reveals deep-intronic variants in Stargardt disease. *Hum Mutat* **40**: 1749-1759.

13. Jespersgaard, C, Fang, M, Bertelsen, M, Dang, X, Jensen, H, Chen, Y, Bech, N, Dai, L, Rosenberg, T, Zhang, J*, et al.* (2019). Molecular genetic analysis using targeted NGS analysis of 677 individuals with retinal dystrophy. *Sci Rep* **9**: 1219.

14. Khan, M, Cornelis, SS, Pozo-Valero, MD, Whelan, L, Runhart, EH, Mishra, K, Bults, F, AlSwaiti, Y, AlTalbishi, A, De Baere, E*, et al.* (2020). Resolving the dark matter of ABCA4 for 1054 Stargardt disease probands through integrated genomics and transcriptomics. *Genet Med* **22**: 1235-1246.

15. Sangermano, R, Khan, M, Cornelis, SS, Richelle, V, Albert, S, Garanto, A, Elmelik, D, Qamar, R, Lugtenberg, D, van den Born, LI*, et al.* (2018). ABCA4 midigenes reveal the full splice spectrum of all reported noncanonical splice site variants in Stargardt disease. *Genome Res* **28**: 100-110.

16. Cremers, FPM, Lee, W, Collin, RWJ, and Allikmets, R (2020). Clinical spectrum, genetic complexity and therapeutic approaches for retinal disease caused by ABCA4 mutations. *Prog Retin Eye Res* **79**: 100861.
