# Supplementary material for: Lipopeptide-mediated Cas9 RNP delivery: A promising broad therapeutic strategy for safely removing deep-intronic variants in ABCA4
Source: Mol Ther Nucleic Acids. 2024 Sep 26;35(4):102345. doi: 10.1016/j.omtn.2024.102345 (PMC11531624; doi:10.1016/j.omtn.2024.102345)
Supplement: Document S2. Article plus supplemental information [file mmc4.pdf]

Molecular Therapy
Nucleic Acids
Original Article

Lipopeptide-mediated Cas9 RNP delivery: A promising broad therapeutic strategy for safely removing deep-intronic variants in *ABCA4*

Irene Vázquez-Domínguez,[1,6] Mert Öktem,[2,6] Florian A. Winkelaar,[1] Thai Hoang Nguyen,[2] Anita D.M. Hoogendoorn,[3] Eleonora Roschi,[1] Galuh D.N. Astuti,[1,4] Raoul Timmermans,[1] Nuria Suárez-Herrera,[1] Ilaria Bruno,[1] Albert Ruiz-Llombart,[1] Joseph Brealey,[5] Olivier G. de Jong,[2] Rob W.J. Collin,[1] Enrico Mastrobattista,[2,7] and Alejandro Garanto[1,3,7]

[1]Radboud University Medical Center, Department of Human Genetics, 6525 GA Nijmegen, the Netherlands; [2]Department of Pharmaceutics, Utrecht Institute for Pharmaceutical Sciences (UIPS), Faculty of Science, Utrecht University, 3584 CG Utrecht, the Netherlands; [3]Radboud University Medical Center, Amalia Children's Hospital, Department of Pediatrics, 6525 GA Nijmegen, the Netherlands; [4]Center for Biomedical Research, Faculty of Medicine, Diponegoro University, Semarang 50275, Indonesia; [5]NanoFCM Co Ltd. MediCity, D6 Thane Road, Nottingham NG90 6BH, UK

Deep-intronic (DI) variants represent approximately 10%–12% of disease-causing genetic defects in *ABCA4*-associated Stargardt disease (STGD1). Although many of these DI variants are amenable to antisense oligonucleotide-based splicing-modulation therapy, no treatment is currently available. These molecules are mostly variant specific, limiting their applicability to a broader patient population. In this study, we investigated the therapeutic potential of the CRISPR-Cas9 system combined with the amphipathic lipopeptide C18:1-LAH5 for intracellular delivery to correct splicing defects caused by different DI variants within the same intron. The combination of these components facilitated efficient editing of two target introns (introns 30 and 36) of *ABCA4* in which several recurrent DI variants are found. The partial removal of these introns did not affect *ABCA4* splicing or its expression levels when assessed in two different human cellular models: fibroblasts and induced pluripotent stem cell-derived photoreceptor precursor cells (PPCs). Furthermore, the DNA editing in STGD1 patient-derived PPCs led to a ∼50% reduction of the pseudoexon-containing transcripts resulting from the c.4539+2001G>A variant in intron 30. Overall, we provide proof-of-concept evidence of the use of C18:1-LAH5 as a delivery system for therapeutic genome editing for *ABCA4*-associated DI variants, offering new opportunities for clinical translation.

INTRODUCTION

Stargardt disease (STGD1) is the most frequently inherited macular degeneration, with a prevalence of ∼1:8,000–10,000 individuals worldwide.[1–4] It is caused by biallelic variants in the *ABCA4* gene,[5] which encodes a transporter protein that localizes within the outer rim of the disks (rod) and invaginations (cone) of the photoreceptor's outer segment and actively removes toxic by-products of the visual cycle.[6] To date, more than 1,200 disease-causing variants have been identified and, among them, deep-intronic (DI) variants account for approximately 10%–12% of the STGD1 cases.[7] These DI variants can lead to the creation of splicing regulatory elements as well as new acceptor or donor splice sites, leading to splicing defects such as pseudoexon (PE) inclusion. These splicing defects often alter the mRNA open reading frame, resulting in premature stop codons, which lead to either the degradation of the mRNA or the synthesis of a truncated and most likely non-functional protein.[8] Among the 49 introns of *ABCA4*, there are two that present a higher density of causative DI variants. This is the case for introns 30[9–16] and 36,[10,12,17,18] in which nine and seven pathogenic DI variants, respectively, have been described (Table S1). Most of the DI in these hot-spots are rare or ultrarare events (i.e., found in one or few individuals), with the exception of a small portion of variants that are relatively recurrent in STGD1 patients (i.e., found in more than 10 alleles according to LOVD; https://databases.lovd.nl/shared/variants/ABCA4/unique; last accessed on May 6, 2024; Table S1). One such example is variant c.4539+2001G>A,[8,12–14,18] which is a frequent DI variant associated with this disorder.[8] This causes the inclusion of a retina-specific 345-nt PE.[14] The same PE can also result from the presence of two other DI variants in intron 30: c.4539+2028C>T[8,14,19] and c.4539+2064C>T.[11,19] Similarly, DI variants in intron 36 result in the inclusion of different PEs. For instance, c.5196+1137G>A, the most frequent variant in intron 36, causes the inclusion of a PE of 73 nt.[8,15] Another relatively recurrent DI variant is c.5196+1056A>G, which generates the inclusion of a 117-nt PE.[8,15]

Received 28 December 2023; accepted 24 September 2024;
https://doi.org/10.1016/j.omtn.2024.102345.

[6]These authors contributed equally

[7]These authors contributed equally

Correspondence: Alejandro Garanto, Department of Pediatrics and Department of Human Genetics, Radboud University Medical Center, Postbus 9101 (855), 6500 HB Nijmegen, the Netherlands.
E-mail: alex.garanto@radboudumc.nl

© 2024 The Author(s).
Published by Elsevier Inc. on behalf of The American Society of Gene and Cell Therapy.
This is an open access article under the CC BY license (http://creativecommons.org/licenses/by/4.0/).

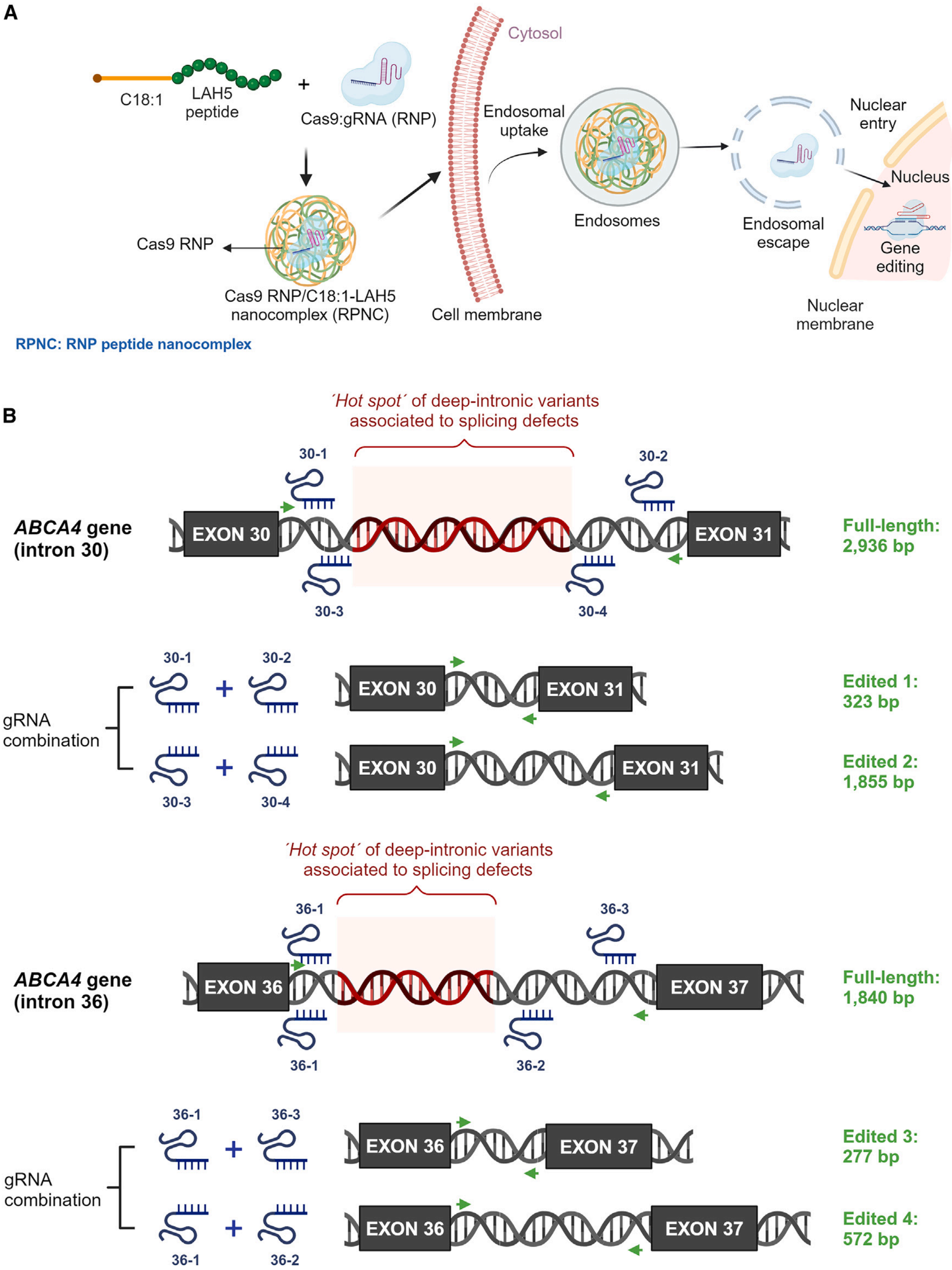

Figure 1. Design of delivery approach and therapeutic strategy

(A) Schematic representation of the proposed mechanism of internalization of the RPNC designed either for gene editing or partial intron removal (PIR). In the acidic endosomal compartments, the C18:1-LAH5 lipopeptide undergoes protonation, leading to the disassembly of the nanocomplexes. This protonation event triggers the release and subsequent escape of the payload from the endosomes, facilitating its delivery to the cytoplasm. Following this, the Cas9 ribonucleoprotein (RNP) enters the nucleus, aided by nuclear localization signals (NLSs) present on Cas9. (B) Schematic representation of intron 30 (upper panel) and intron 36 (lower panel) of *ABCA4* gene. The red

(legend continued on next page)

Based on the aforementioned data, these hot-spot clusters constitute regions with high therapeutic potential. Unfortunately, most of the therapeutic approaches for DI variants in *ABCA4* are based on the use of antisense oligonucleotides (AONs),[7,14,20,21] which, among other functions, allow the correction of splicing defects.[22] Nevertheless, a limitation of these molecules is their mostly variant-specific nature, restricting their therapeutic potential to a single variant or splicing defect rather than a broader group of patients. Conversely, the CRISPR-Cas9 genome-editing technology enables precise targeting of specific regions within the genome.[23] In this case, a specific guide RNA (gRNA) can guide the Cas9 endonuclease to introduce a double-strand break at a specific genomic position, as long as the protospacer adjacent motif (PAM) is present. By combining two gRNAs targeting the flanking regions of an intronic mutation hot spot, the splicing defects resulting from different pathogenic variants can be corrected using a single therapeutic approach.[24–26] One of the limiting factors of genome editing by CRISPR-Cas9 is the efficient delivery of the Cas9 and gRNAs into the target retinal cell type.[27] To solve this, various approaches have been explored, such as the use of viral-based vector systems[28] or Cas9 orthologs.[29] Recently, swift advancements in nonviral delivery vectors, encompassing lipid,[30–33] polymer,[34,35] peptide,[36–39] and inorganic nanoparticle-based delivery systems,[40–42] and nanoclews[43] have firmly established nonviral delivery methodologies as a feasible substitute for viral vectors. Among them, peptides and peptide-like materials offer the possibility to extensively engineer properties that facilitate the delivery of Cas9 ribonucleoproteins (RNPs), irrespective of their size and molecular weight. Peptide-mediated Cas9 RNP delivery can be accomplished through covalent conjugation to the Cas9 protein[44,45] or noncovalent ionic interactions with the overall negatively charged Cas9 RNP.[37,46–48] With respect to covalent peptide conjugates, the Cas9 RNP remains exposed, potentially leading to degradation of the protein or single gRNA (sgRNA). Previous studies in our group demonstrated that the use of amphipathic LAH5 peptide (NH2-KKALLALA LHHLAHLAHHLALALKKA-COOH), which was initially developed for the delivery of plasmid DNA,[49] can be repurposed for the delivery of Cas9 RNP and shows high levels of both gene editing and gene correction across various cell types.[50] Notwithstanding these promising results, data were obtained under low serum conditions, and the stability of the nanocomplexes was suboptimal and required a large excess of LAH5 peptide compared to Cas9 RNP to maintain the integrity of the nanocomplexes. To make the Cas9 RNP/peptide nanocomplexes more stable and to protect the peptide from serum-derived factors and other potentially detrimental conditions, a commonly employed strategy involves the conjugation of the peptide with a fatty acid moiety.[51–53] Several of these lipopeptides have been tested for delivery of RNPs to a wide range of cell types.[46–48]

This study investigates the delivery of Cas9 RNPs, both alone and combined with dual gRNAs, to fibroblast and photoreceptor precursor cells (PPCs) using oleic acid-modified LAH5 peptides (C18:1-LAH5) (Figure 1A), aiming to explore a mutation-independent approach for targeting multiple DI variants in intron 30 or intron 36 of the *ABCA4* gene associated with STGD1 (Figure 1B).

RESULTS

C18:1-LAH5 form stable nanocomplexes with Cas9 RNP

The design and proposed mechanism of cellular uptake of Cas9 RNP/C18:1-LAH5 nanocomplexes (referred to as RPNCs from now on) for both gene editing and partial intron removal (PIR) are depicted in Figure 1A. Dynamic light scattering (DLS) was used to monitor RPNC formation of the C18:1-LAH5 lipopeptide with RNP. The results showed that higher Cas9 RNP:lipopeptide ratios led to a decrease in the average size of the formed RPNCs. Varying the lipopeptide and RNP ratios, starting from a 50× ratio, resulted in RPNCs displaying average particle sizes ranging from 191 to 151 nm, with a polydispersity index (PDI) around 0.3 (Figure 2A). Moreover, leaving out the negatively charged gRNA in the formation of RPNCs resulted in a much bigger average particle size as measured by DLS (408 ± 44 nm) compared to those of RPNCs with gRNA, suggesting that complex formation is at least partially dependent on electrostatic interaction (Figure 2A). The ζ-potential of these RPNCs increased as C18:1-LAH5 lipopeptide concentrations increase, with ζ-potential values of approximately 2.86 mV at 50× molar ratio of lipopeptide, 3.89 mV at 200×, and 4.19 mV at 250× (Figure 2B).

RPNC formation was further validated by an electrophoretic mobility shift assay (EMSA). Both GFP-Cas9 and ATTO647-gRNA showed a significantly reduced mobility as the lipopeptide concentration increased with complete retention of both molecules in the gel slots at a C18:1-LAH5 molar excess of ≥50× (Figures 2C and S1).

Based on these data, we concluded that the C18:1-LAH5 lipopeptide is able to form stable complexes with RNP, starting from a 1:1:50 molar ratio relative to gRNA and Cas9 (Figure S1). Importantly, the findings also demonstrated that C18:1-LAH5 lipopeptide not only could create RPNCs with negatively charged sgRNA and RNP but could also complex with the Cas9 protein. This most likely suggests the involvement of non-electrostatic hydrophobic interactions (Figure S1).

Effective uptake of RPNCs in HeLa cells

To visualize cellular uptake of the RPNCs, fluorescence confocal imaging was conducted 24 h after RPNC addition, in which the RPNCs were fluorescently labeled with GFP-Cas9 or with ATTO550-gRNA (Figure 3A). The confocal microscopy images showed internalization of RPNCs into cells, which is shown by the green and red signals from GFP-Cas9 and ATTO550-gRNA, respectively (Figure 3A). As expected, the cell-associated fluorescence of GFP-Cas9 and ATTO550-gRNA was notably enhanced for RPNCs as compared to free Cas9 RNP (Figures 3A, S2A, and S2B). Both punctate fluorescence (reminiscent of endocytic uptake) and more faint, diffuse fluorescence

double-stranded DNA indicates the hot-spot area in which several intronic variants causing splicing defects have been reported. The PCR amplification primers are indicated with green arrows and the designed gRNAs are shown in their target position. The size of the full-length amplicon is indicated on the right. Below, the combination of the different gRNAs together with the scheme of the expected fragment and its size at genomic level are represented.

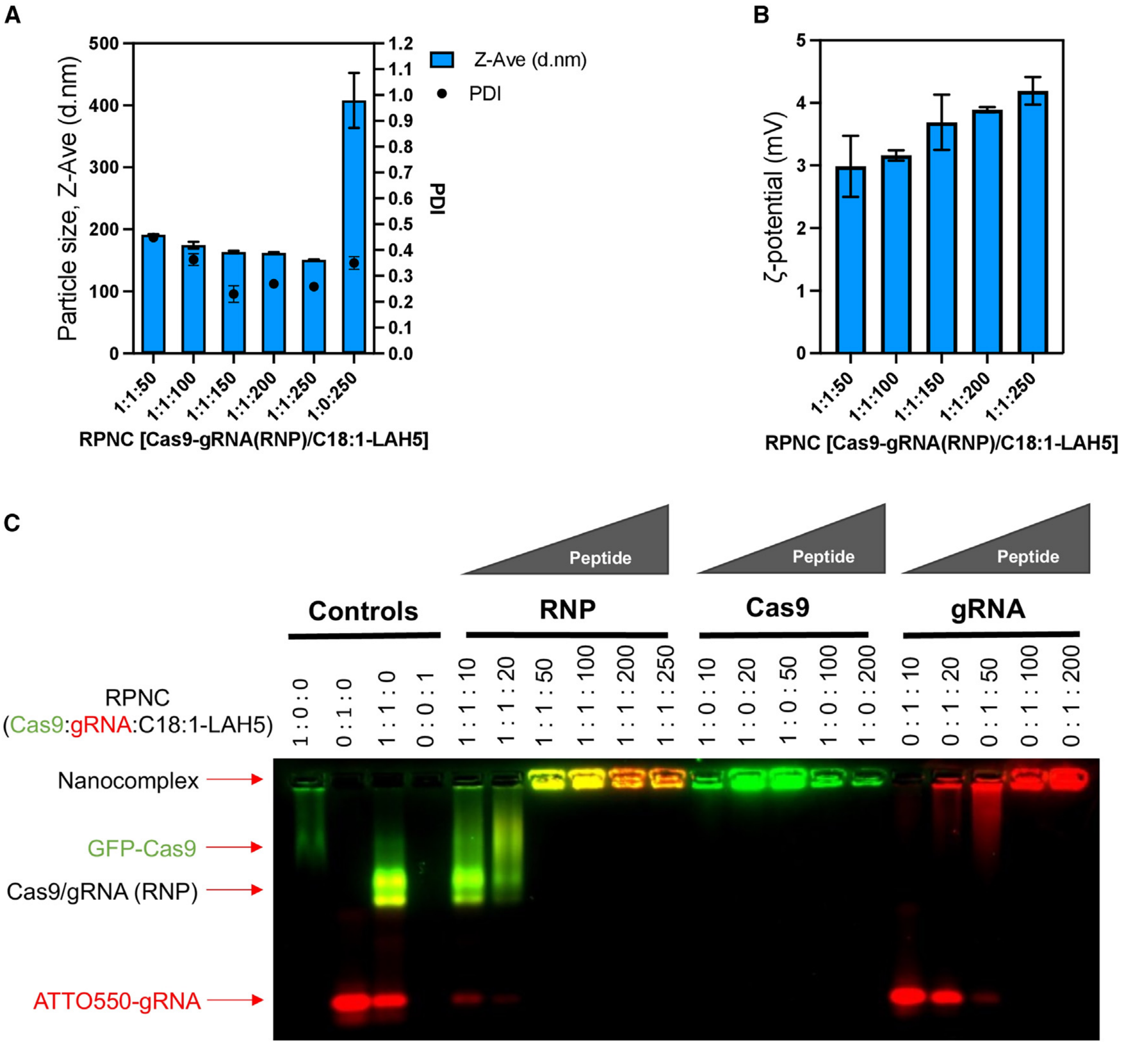

Figure 2. Characterization of RPNCs at different ratios of RNP and C18:1-LAH5 lipopeptide

(A) The particle size and polydispersity index (PDI) of the RPNC prepared at increasing molar ratios of Cas9 RNP to C18:1-LAH5 lipopeptide (20–20 nM)/increasing molar ratios of lipopeptide (RPNC) were determined using DLS. Data shown as mean ± SD (n = 3). Z-Ave parameter represents the intensity-weighted average hydrodynamic diameter of the particles in the sample and therefore describes the particle size of a sample. The higher the Z-Ave value, the larger the particle size. (B) ζ-Potential of 20 nM Cas9/gRNA (RNP) complexed with increasing molar ratios of C18:1-LAH5 lipopeptide was measured; data shown as mean ± SD (n = 3 technical repeats). (C) Electrophoretic mobility shift assay (EMSA) showing the effect of increasing C18:1-LAH5 lipopeptide concentration on RPNC formation. Controls: 1:0:0 (only GFP Cas9), 0:1:0 (only ATTO550 gRNA), 1:1:0 (RNP [GFP Cas9 and ATTO 550 gRNA]), and 0:0:1 (only C18:1-LAH5 lipopeptide). Three experimental setups were employed: (1) a combination of GFP-Cas9, ATTO550-gRNA, and the lipopeptide (indicated as RNP); (2) GFP-Cas9 along with the lipopeptide (indicated as Cas9); (3) ATTO550-gRNA in conjunction with the lipopeptide (indicated as gRNA). Within the framework of each of these configurations, the resulting RNPs were complexed with the range of lipopeptide concentrations, ranging from 0 to 250 μM.

(suggesting cytosolic delivery) could be observed, especially for the gRNA signal. This observation highlighted the ability C18:1-LAH5 lipopeptide to augment the internalization of RNP. Notably, a fainter LysoTracker fluorescence intensity was observed in samples incubated with RPNCs containing GFP-labeled Cas9 compared to GFP-Cas9 RNPs (Figures 3A and S2C). As the fluorescence intensity of LysoTracker as well as its retention in endosomes and lysosomes is pH dependent, this suggests that endosomal uptake of RPNC leads to an increased pH within the endosomal lumen, which may be an indirect indication of disruption of the pH gradient over the endosomal membrane as a consequence of membrane disruption by the LAH5 peptide.

To confirm cytosolic and subsequent nuclear delivery of Cas9 RNP complexes with RPNCs, a prerequisite for gene editing, Cas9-mediated genomic indel formation at a randomly chosen target locus (*CCR5* gene) was assessed with a T7 endonuclease 1 (T7E1) assay. DNA samples from cells treated with RPNCs showed the distinctive

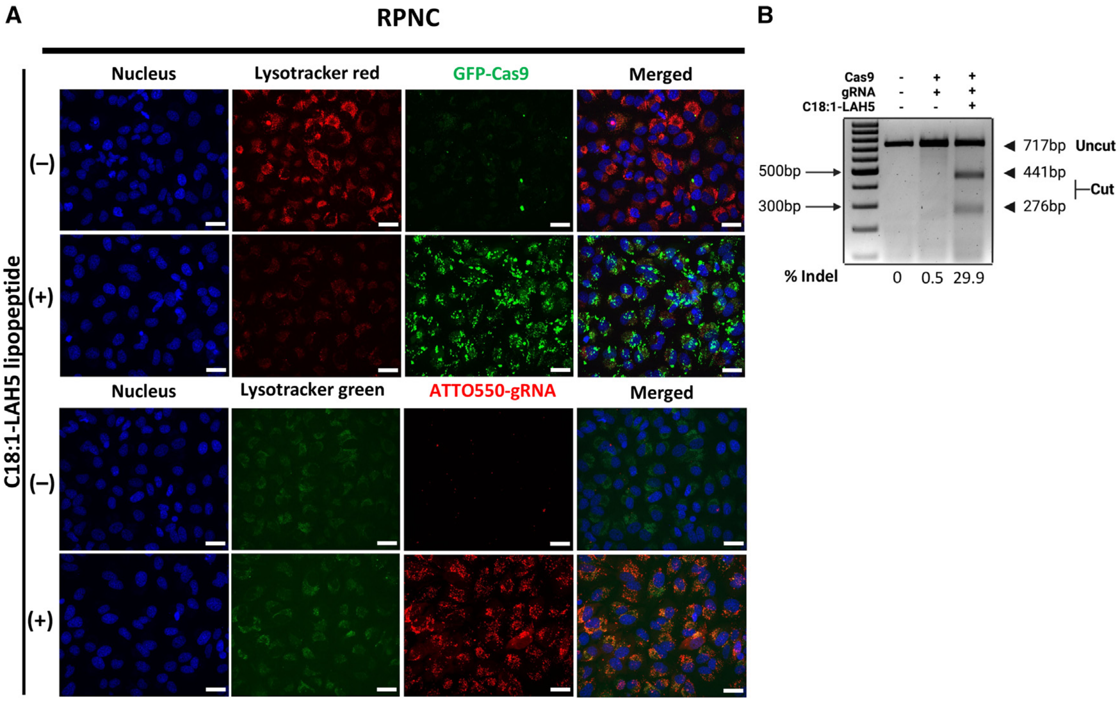

Figure 3. Cellular uptake of RPNC and functional delivery of Cas9 RNP as measured by heteroduplex cleavage with T7E1 at the targeted *CCR5* locus
HeLa cells were treated with two different RPNCs, consisting of RPNC including GFP-Cas9/gRNA/C18:1-LAH5, or non-labeled Cas9/ATTO550-gRNA/C18:1-LAH5, at a molar ratio of 1:150 for RNP formulation. Negative controls consisted of Cas9 RNP without the inclusion of C18:1-LAH5. The same contrast and brightness were used for control and C18:1-LAH5 conditions in each fluorescence channel. (A) HeLa cells were transfected with 20 nM eGFP-Cas9/gRNA and 20 nM non-labeled Cas9/ATTO550-gRNA either without (upper) or with (lower) C18:1-LAH5. Twenty-four hours after transfection, the lysosomes were stained with LysoTracker (red or green) for 30 min before fluorescence microscopy images were taken at 60× magnification. Scale bar, 50 μm. (B) Functional delivery was evaluated using a T7EI assay. Genomic DNA was extracted from both the control and treated samples, and an 800-nt fragment of the *CCR5* locus was amplified by PCR. Subsequently, the PCR product was digested with T7EI to examine heteroduplex formation. The frequency of indels was calculated using the equation provided in the "materials and methods" section.

DNA cutting bands of 276 and 441 nt indicating the formation of DNA heteroduplexes at the target locus. Band-intensity calculations yielded an indel frequency of 29.9% for the RPNC (Cas9 RNP/C18:1-LAH5)-treated sample (Figure 3B). These findings demonstrate that Cas9 RNP complexation with an excess of C18:1-LAH5 lipopeptide resulted in effective intracellular delivery of intact Cas9 RNP, leading to subsequent gene editing within the cell nucleus.

RPNCs mediate efficient gene editing in reporter cells

After characterizing RPNCs, we evaluated their gene editing efficiency within two distinct reporter cell lines: HEK293T stoplight[54] and eGFP HEPA 1–6 cells[55] (Figure 4). Additionally, alongside RPNCs including C18:1-LAH5, RPNC formed with LAH5 peptide[50] was also examined. Of note, HEK293T stoplight cells continuously express mCherry. The CRISPR-Cas9 system was used to induce indel formation downstream of the mCherry coding sequence, which for +1 or +2 frameshift mutations leads to co-expression of eGFP[54] (Figure 4A). Conversely, eGFP HEPA 1–6 cells exhibit constitutive eGFP expression, with the loss of eGFP signal indicating gene editing (Figure 4B).

Prior to transfection, the potential cytotoxicity of the RPNCs to HEK293T stoplight and eGFP HEPA 1–6 cells was assessed using an MTS assay (Figures S3A and S3C). The results showed that RPNCs made with RNP:lipopeptide ratios ranging from 1:50 to 1:250 maintained over 80% cell viability in both HEK293T and eGFP HEPA 1–6 cells after 24-h incubation (Figures S3A and S3C).

To assess delivery efficiency, HEK293T stoplight cells and eGFP HEPA 1–6 cells were incubated with RPNCs containing a constant Cas9 RNP concentration of 20 nM alongside increasing concentrations of either LAH5 peptide or C18:1-LAH5 lipopeptide for 48 h. Cellular fluorescence was quantified via flow cytometry. This analysis revealed that increasing peptide concentrations in the presence of a fixed Cas9 RNP concentration (20 nM) led to augmented gene editing, regardless of peptide type in RPNCs or cell type (Figures 4C and 4D).

At the optimal RNP:peptide ratio, which was determined to be 1:250 for both types of RPNC, we achieved a 40% induction rate of eGFP in HEK293T stoplight cells and a 31% knockout rate of eGFP in eGFP HEPA 1–6 cells using RPNC (RNP/LAH5). However, the utilization of RPNC (RNP/C18:1-LAH5) led to a significant enhancement in gene editing efficiency, reaching a 63% induction rate of eGFP in HEK293T cells and 66% knockout rate of eGFP in eGFP HEPA 1–6

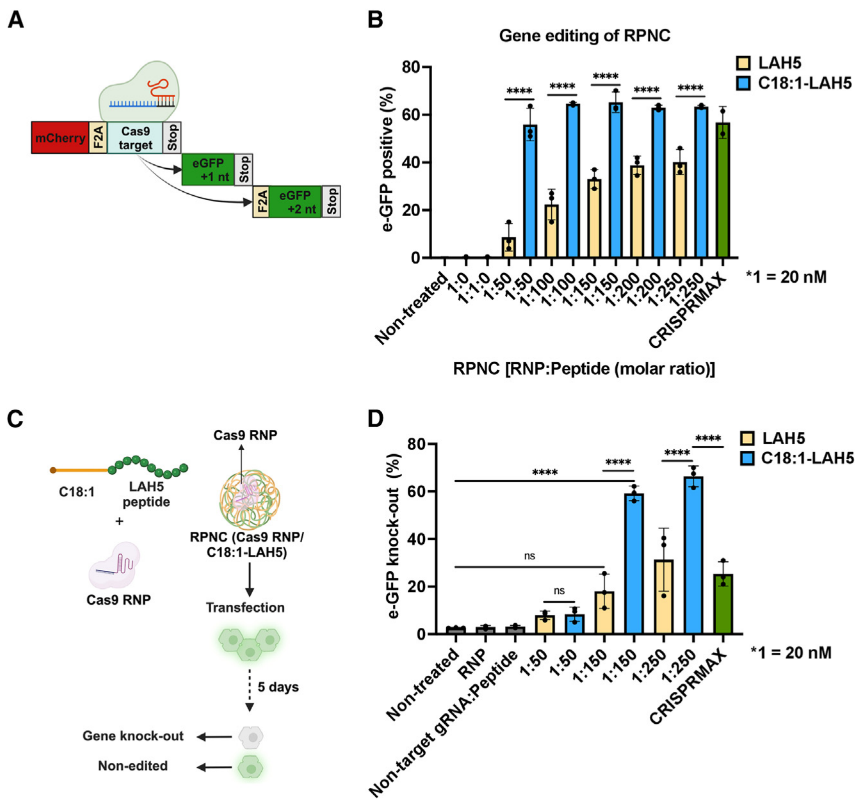

Figure 4. RPNC formulation optimization for gene editing in HEK293T stoplight and eGFP HEPA 1–6 reporter cells

(A) Schematic illustration of HEK293T stoplight cells reporter construct. These cells constitutively express mCherry and will start to express eGFP upon introduction of a +1 +2 frameshift within the linker region downstream of the mCherry gene. (B) Gene-editing efficiency for both LAH5 peptide and C18:1-LAH5 lipopeptide is represented by the e-GFP induction. Formulations ranging from 0 to 5 μM peptide concentrations were tested on HEK293T stoplight cells and quantified by flow cytometry. Representative analysis of flow cytometry analysis can be found in Figure S18. (C) Schematic illustration of the mechanism for peptide-mediated RNP delivery and the mechanism of reporter eGFP HEPA 1–6 cells. The reduction in the eGFP signal serves as an indicator of gene editing. (D) The quantification of gene-editing efficiency was quantified using flow cytometry 5 days after transfection, wherein the percentage of eGFP knockout was measured. eGFP knockout efficiency of RPNC (RNP/peptide) compared with negative (NT) and positive (CRISPRMAX, in green) controls. To assess optimal RPNC dose for gene editing, eGFP HEPA 1–6 cells were exposed to RPNCs prepared with either LAH5 or C18:1-LAH5 lipopeptide at various molar ratios ranging from 1:50 to 1:250. Representative flow cytometry analysis can be found in Figure S19. Data are shown as the mean ± SD (n = 3), Tukey's multiple comparisons test (ns, non-significant; *$p < 0.05$, **$p < 0.01$, ***$p < 0.001$, ****$p < 0.0001$).

cells (Figures 4B and 4D). Remarkably, RPNCs (RNP/C18:1-LAH5) exhibited notably higher gene editing efficiencies when compared to the commercial positive control CRISPRMAX for RNP delivery in both cell types, and this difference reached statistical significance ($p < 0.01$) in the eGFP HEPA 1–6 cells (Figure 4D). Conversely, RPNC (RNP/LAH5) displayed lower gene-editing rates in both cell types compared to CRISPRMAX (Figures 4B and 4D).

At RNP/peptide ratios of 1:150 and 1:250, RPNCs formulated with C18:1-LAH5 lipopeptide distinctly outperformed the LAH5 RPNCs in both cell types (Figures 4B and 4D). This indicates that the increased effectiveness of the peptide-mediated delivery system can be ascribed to the incorporation of a C18:1 fatty acid tail. In HEK293T stoplight cells treated with RPNC (RNP/C18:1-LAH5) consistently achieved approximately 60%–70% gene editing at all tested lipopeptide ratios (Figure 4B). Conversely, RPNCs (RNP/LAH5), particularly at lower peptide ratios such as 1:50, achieved a gene editing rate of only about 10% (Figure 4B). These findings highlight the increased stability and superior performance of RPNCs formulated with C18:1-LAH5 across different ratios and reporter cells.

Having demonstrated that RPNCs can efficiently induce gene editing via Cas9-mediated targeted indel formation in various cells in culture, we next investigated the deletion of intronic regions from the genome as a means to correct DI variants leading to STGD1.

Design and test of gRNAs on genomic DNA level in HEK293T cells showed high efficiency in editing both intron 30 and intron 36

Based on the fact that there are two introns in which a significant number of DI variants are found (intron 30 and intron 36), we hypothesized that the partial removal of the corresponding intron could be a therapeutic strategy to target multiple DI variants simultaneously. Different gRNA sequences were designed along introns 30 and 36 (upstream and downstream of the variant cluster, respectively). We hypothesized that, by combining two gRNAs (one upstream and one downstream of the DI cluster), the cluster can be removed, leading to the prevention of PE formation. In total, 12 gRNAs were designed for intron 30 (Table S2). The same approach was followed for intron 36, for which a total of six gRNAs were designed to remove the pathogenic DI-variant-containing region present in this intron (Table S2).

Hereafter, gRNA sequences were individually cloned into plasmid pX458, which expresses the Cas9 protein alongside the designed gRNAs. Subsequently, gRNAs were tested in pairs in HEK293T cells to assess their genome-editing capacity (Figure S4). From all the tested pairs, the two best-performing pairs were selected to conduct the following experiments in other cellular models. For intron 30, the pair 30 1 + 2 and 30 3 + 4 were selected, since both presented the best editing efficacies. Besides this, pair 30 1 + 2 was able to remove a bigger part of intron 30, including eight out of nine of the pathogenic

A

Ratio:RPNC (Cas9/gRNA/ C18:1-LAH5)	Size(d.nm)[a]	PDI[a]	ζ-potential (mV)[b]
1:2:50	199±24	0.3±0.1	3.2±0.6
1:2:100	171±4	0.36±0.06	3.7±0.4
1:2:150	147±3	0.25±0.02	4.4±0.2
1:2:250	144±4	0.24±0.02	4.5±0.2

[a] Determined by DLS.
[b] Determined by Zetasizer.

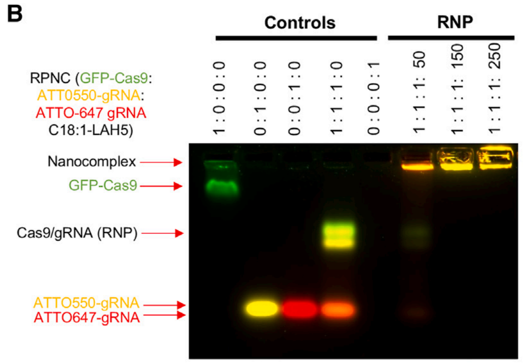

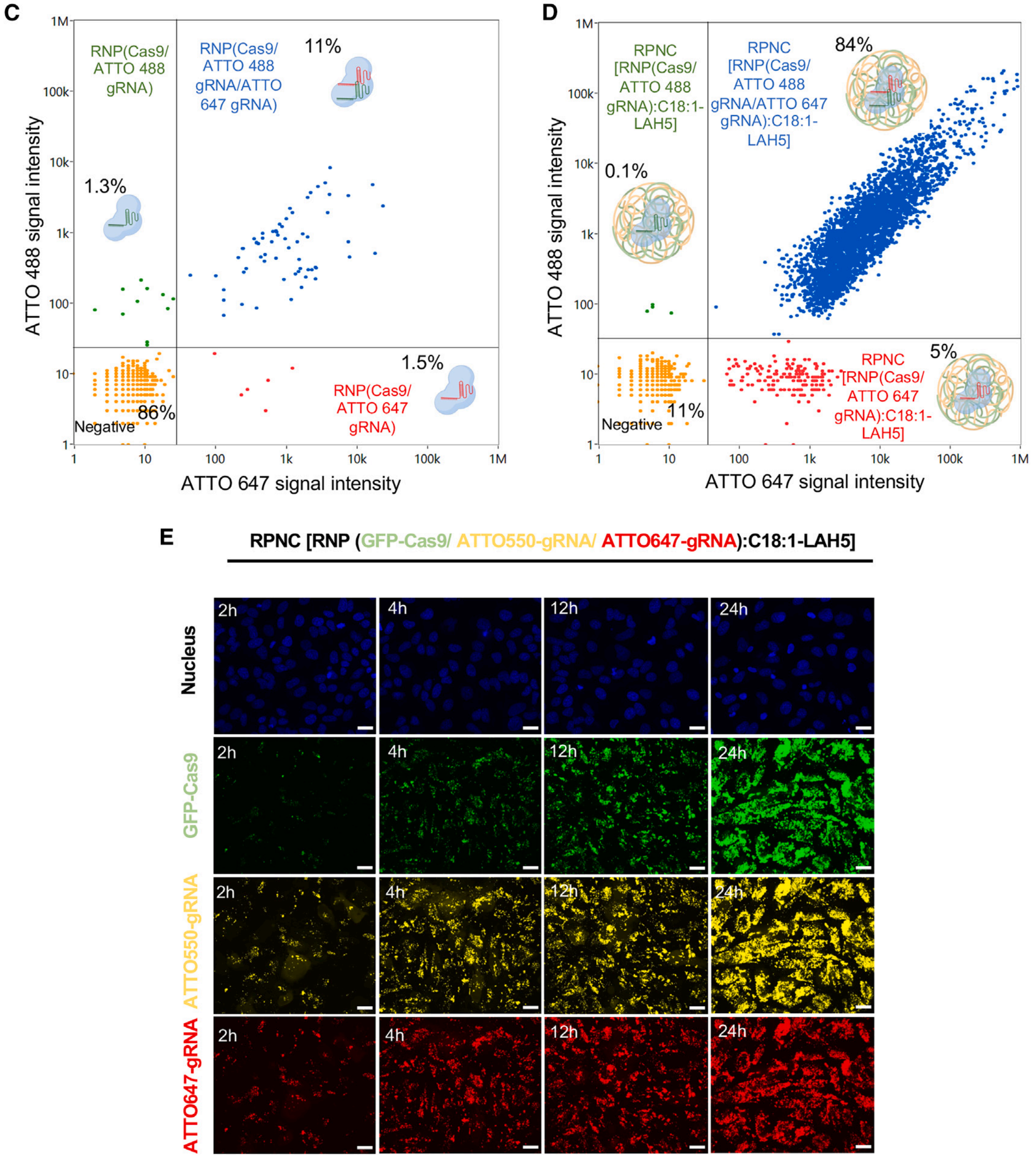

(legend on next page)

variants on Table S1 (c.4540-5T>A is not covered) (Figure S4; left). In intron 36, pairs 36 1 + 2 and 1 + 3 were selected as they showed the highest editing levels. Moreover, again these two combinations removed a region of intron 36 of 1,563 or 1,268 nt, respectively, thus increasing the number of variants that could benefit from this approach (Figure S4, right). Both pair 36 1 + 2 and pair 36 1 + 3 were able to target four out of six pathogenic variants listed on Table S1 (c.5196+1013A>G, c.5196+1056A>G, c.5196+1137G>A, and c.5196 +1216C>A).

RPNCs for PIR led to efficient co-delivery of both gRNAs and Cas9 into cells

After demonstrating the functionality of the two gRNAs for PIR, we next formulated both gRNAs into RPNCs. RPNC characterization containing two gRNAs revealed that, above Cas9:gRNA:C18:1-LAH5 lipopeptide ratios of 1:2:50 to 1:2:250, both guides were complexed, leading to stable RPNCs in the size range of 150–175 nm (Figures 5A and 5B). NanoFCM analysis, a flow-cytometry-based method to determine size and fluorescence intensities of single RPNCs within the size range of 50–2,000 nm, confirmed the presence of both gRNAs in individual RPNC, as shown in Figures 5D and S5 and Table S3. The majority of RPNCs were double positive, and only a small fraction of RPNCs contained a single gRNA RNP. Control sample containing the same amount of Cas9 RNP but without the C18:1-LAH5 lipopeptide showed around 70-fold fewer events (Figures 5C and S5C), indicating that RPNC formation requires the presence of C18:1-LAH5. The few events that are visible are most likely Cas9 RNP aggregates.

Confocal imaging was conducted to assess cellular uptake of RPNCs containing GFP-Cas9, ATTO550 gRNA1, ATTO647 gRNA2, and C18:1-LAH5 over time, as illustrated in Figures 5E and S6. In this experiment, HeLa cells were incubated with RPNCs for PIR. Confocal microscopy analysis revealed a synchronized increase in fluorescence intensities of all three labeled components, suggesting co-delivery of these components into these cells. This uptake process occurred consistently over a time span from 2 to 24 h (Figure 5E).

Genome editing was achieved in control and patient-derived fibroblast cells

Having confirmed that the C18:1-LAH5 RPNC can efficiently co-deliver Cas9 RNP with two different gRNAs, we next aimed to apply this to partially remove the pathogenic DI-variant-containing region in introns 30 and 36 of the *ABCA4* gene as a potential therapeutic approach (Figure 1B). To this end, fibroblast cells derived from both a healthy individual and an STGD1 patient carrying the variant c.4539+2001G>A in a heterozygous manner were treated using RPNCs. One of the gRNA pairs was selected to determine the best transfection conditions. The optimal ratio of Cas9:gRNAs/C18:1-LAH5 to make RPNCs for PIR was determined by a dose-curve experiment (Figure S7A). Once the optimal conditions were established, the RPNCs were delivered to both control and patient cells. In both cases, RPNC delivery was performed and was followed by a 10-day trans-differentiation protocol to boost the expression levels of *ABCA4* mRNA and PE. Of note, as the *ABCA4* pathogenic variant (c.4539+2001G>A) present on the patient line is located in intron 30, only the gRNAs against that region were tested in this patient cell line allowing DNA and RNA analyses of the genome-edited cells. In contrast, the gRNA pair for intron 30 and the gRNA pair of intron 36 were both tested in the control fibroblast line to evaluate the genome-editing capacity at the DNA level. For intron 30, our results showed that the gRNA pair 30 1 + 2 was capable of partially removing intron 30 with higher efficacy than the gRNA pair 30 3 + 4 in both control and patient fibroblasts. However, the editing levels in both cell lines were quite similar, being around 74% ± 23% and 78% ± 14% of editing in control and patient fibroblasts, respectively (Figure 6A). In some cases, we also observed some unspecific bands. However, these are also detected in the non-treated conditions pointing to PCR artifacts rather than a non-desired off-target effect of gene editing.

The gene editing detected in intron 36 in the control fibroblast cell line revealed that both pairs of gRNAs (gRNAs 36 1 + 2, and gRNAs 36 1 + 3) were able to induce the partial deletion of intron 36 (Figure 6B). The efficacy of both gRNA pairs was similar to, albeit slightly higher than, gRNA pair 36 1 + 2 as compared to the gRNA pair 36 3 + 4 (∼41.8% ± 17% versus ∼35% ± 22%). In this case, we observed unspecific bands in the edited conditions but not in the treated samples. However, sequencing analysis did not reveal any clear sequences associated with the gRNAs or the intron 36 region.

In addition to DNA analysis, RNA was examined to determine if genome editing corrected the splicing defect caused by the c.4539+2001G>A variant. Initially, we investigated the splicing pattern by selectively amplifying an amplicon from exon 30 to exon 31 (Figure S8A). Our analysis did not reveal statistically significant differences between the non-treated (NT) conditions and those

Figure 5. Representation of the characterization, simultaneous delivery, and cellular uptake of RPNCs with two different gRNAs for the application of PIR
(A) Particle size and ζ-potential characterization of RPNCs formulated with Cas9 and gRNA at a 1:2 M ratio and with increasing amounts of C18:1-LAH5 lipopeptide (molar ratios indicated as Cas9/gRNA1/gRNA2-lipopeptide), diameter values in nanometers (d.nm) and PDI shown as mean ± SD (*n* = 3), ζ-potential (mV) shown as mean ± SD (*n* = 3 technical replicates). (B) EMSA of RPNCs assembled with Cas9/RNP with two different gRNAs. GFP Cas9, ATTO550-gRNA1, and ATTO647-gRNA2 prepared using these labeled components were used as controls to visualize all separate components. Following complex formation in presence of non-labeled lipopeptide, the samples were analyzed using a 1.5% agarose gel. (C and D) RPNC characterization by NanoFCM, a flow cytometry method to determine size and fluorescence intensities of single nanoparticles >∼50 nm in size. (C) Free Cas9 RNP containing ATTO488-gRNA1 and ATTO647-gRNA2 show only a few events, most likely aggregated Cas9 RNP proteins with sizes >50 nm. (D) RPNC forming from Cas9 RNP and C18:1-LAH5 lipopeptide complexation showed more events, of which 84% were double positive for Cas9/ATTO488 gRNA1 and ATTO647. (E) Confocal imaging of time-dependent cellular uptake of RNP (GFP-Cas9, ATTO550-gRNA1 and ATTO647-gRNA2/C18:1-LAH5 [RPNC]) in HeLa cells. Scale bar, 30 μm.

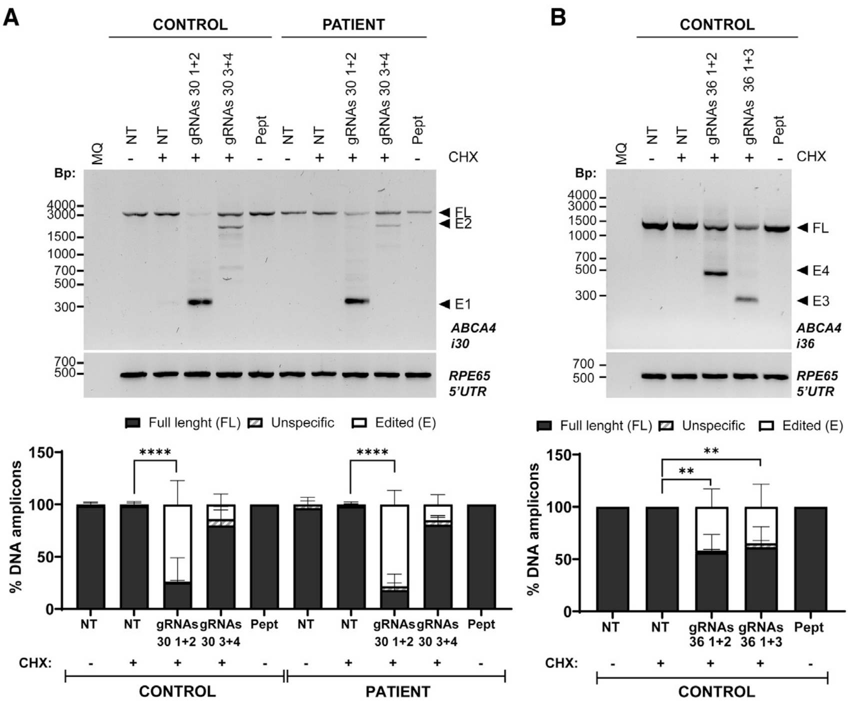

Figure 6. Analysis of PIR efficacy of RPNC-mediated editing in fibroblasts at DNA level either in intron 30 or intron 36 of the *ABCA4* gene

(A) (Upper panel) A representative electrophoresis gel of the amplification of intron 30 by PCR in both control and patient fibroblast cell lines. Edited band 1 (E1) indicates the expected edited band after treating with gRNAs 30-1 and 30-2 while the edited band 2 (E2) represents the expected band after the editing with gRNAs 30-3 and 30-4. The 5′ UTR of *RPE65* was amplified as loading control. (Lower panel) A graph chart representing the overall result of the conducted replicates ($n = 4$) indicating the percentage of the full-length (FL) amplicon, without editing and the percentage of editing (edited band, E) for each condition. Each bar is represented by the mean ± SD ($n = 4$). In addition, some unspecific amplifications were observed that did not correspond to the expected products following editing and amplification. (B) (Upper panel) A representative electrophoresis gel of the amplification of intron 36 of *ABCA4* by PCR in control fibroblast. In this case, the edited band 1 (E4) represents the expected band after treating with RPNCs, including gRNAs 36-1 and 36-2, while edited band 2 (E3) indicates the expected edited band after gRNAs 36-1 and 36-3 mediated editing. The 5′ UTR of *RPE65* was amplified as loading control. (Lower panel) A graph bar representing the percentage of FL, edited (E), and unspecific amplicons per condition. Each bar is represented by the mean ± SD ($n = 2$). MQ indicates the negative control of the PCR; NT, non-treated cells; Pept, cells treated with the lipopeptide but without Cas9 or gRNAs (negative control of the transfection). CHX indicates if cell were treated with (+) or without (−) cycloheximide 24 h before harvesting. Statistical significance with respect to the untreated condition (NT+) is indicated as $^{**}p < 0.01$ or $^{****}p < 0.0001$ using one-way ANOVA followed by Bonferroni correction.

treated with gRNAs, while we could confirm both the success of the trans-differentiation and the nonsense-mediated decay (NMD) inhibition with cycloheximide (CHX). We also assessed the presence of the 345-nt PE using primers from the PE to exon 31. No significant differences in PE exclusion were observed between treated and NT fibroblasts (Figures S8B and S8C). These results are probably explained by the detectable, but still low, *ABCA4* mRNA levels even after trans-differentiation and treatment with CHX. Finally, we evaluated the expression levels of the PE in both control and fibroblast by specific primers using qPCR (Figure S8C), and none of the edited samples showed significantly different expression levels.

Based on all the aforementioned results, gRNA pairs 30 1 + 2 and 36 1 + 2 were selected for studies in PPCs, in which *ABCA4* and the PE are robustly detected.[14]

Genome editing in PPCs showed different efficacies in control and patient cells, but PIR was successfully achieved

Induced pluripotent stem cell (iPSC)-derived PPCs are a heterogeneous culture of retinal-like cells that have been shown to have increased expression levels of *ABCA4*.[7,14,56] Similarly to the studies in fibroblasts, a dose-curve pre-analysis confirmed that the optimal ratio of RPNC for PIR was Cas9:gRNAs/C18:1-LAH5 1:2:150 (Figure S7B) in PPCs. In these studies, two iPSC-derived PPC cultures were employed: the patient cell line carrying the c.4539+2001G>A variant on *ABCA4* in a heterozygous manner derived from the fibroblast cells used in the previous section[57,58] and its corresponding isogenic control iPSCs.[57,58] As was done in fibroblasts, intron 36 gRNAs were only evaluated in the isogenic control PPC line. The previously identified most efficacious pairs of gRNAs for both introns were evaluated (gRNA pair 30 1 + 2 and 36 1 + 2). In the case of the control line, the editing levels reached ∼32%–40% for the RPNCs containing gRNA pair 30 1 + 2 in intron 30 (Figure 7A, left), while, in the patient cells, it showed a slightly higher efficacy, between ∼35% and 47% (Figure 7B). The efficacy of the RPNCs with the pair 36 1 + 2 for intron 36 only reached ∼15% editing (Figure 7A, right). To further confirm that partial removal of the intron did not affect splicing in a retinal context, we conducted RT-PCR analyses by detecting amplicons spanning multiple exon-exon junctions in intron 30 (from 27 to 34) and intron 36 (from exon 34 to 38), as shown in Figure S9. Our analysis revealed that removing (large) portions of these introns did not affect splicing or the *ABCA4* levels in treated PPCs.

Upon confirming genome editing at the DNA level and subsequent unaffected RNA splicing pattern, samples were used to investigate if the insertion of the PE was reduced. Similarly to the fibroblast samples, we initially assessed the splicing pattern through RT-PCR spanning from exon 30 to exon 31 (Figure 8A) to have a small amplicon for better visualization and determination of the PE. While the conditions treated with the RPNCs exhibited a reduction of the PE-containing transcript, it was not statistically significant. However, upon

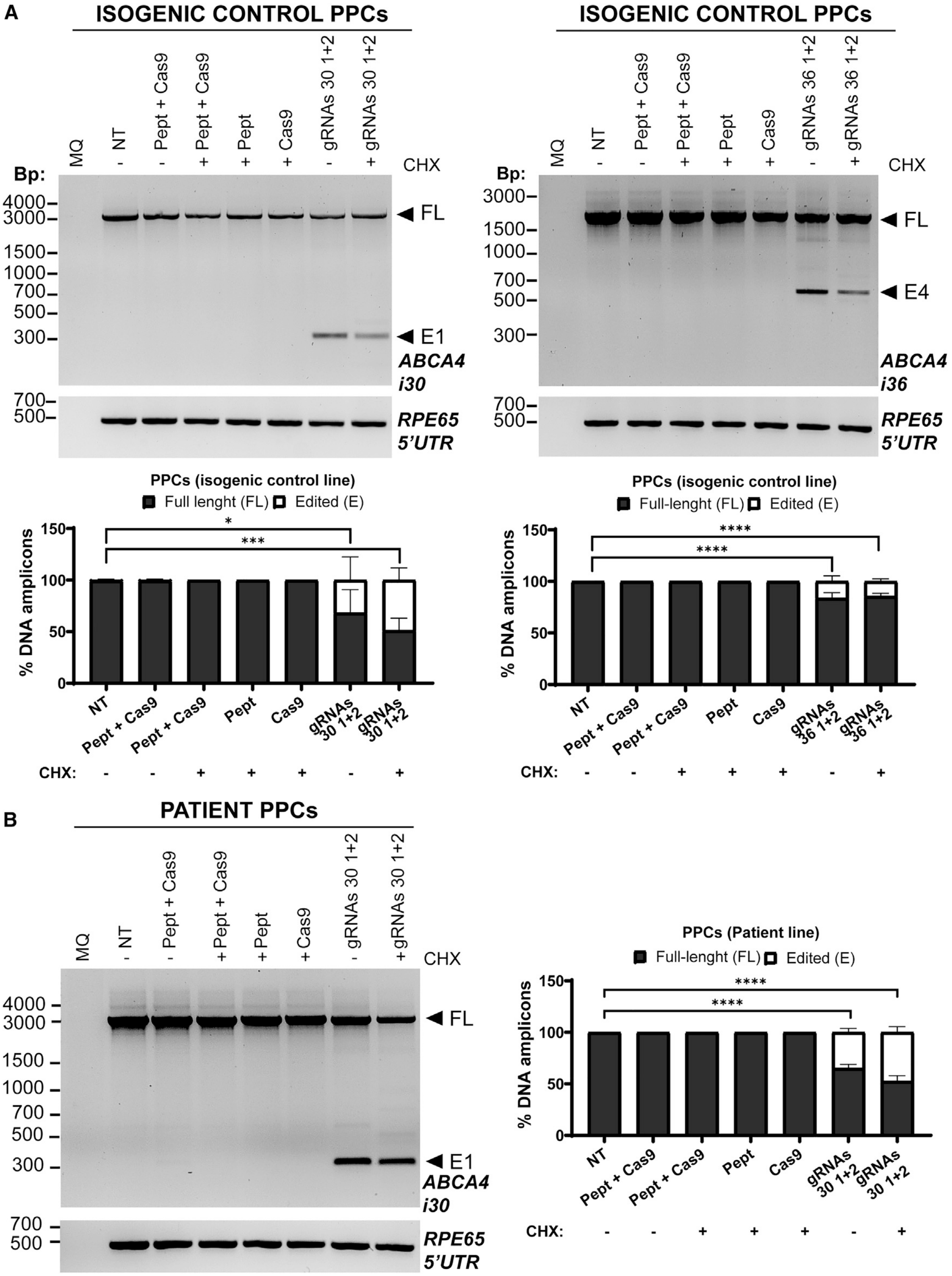

Figure 7. Analysis of the effectiveness of the RPNC-mediated gene editing in photoreceptor precursor cells at genomic DNA level

(A) Isogenic control PPCs and (B) patient-derived PPCs. (A) (Top) Representative electrophoresis gels of the amplification of the intron 30 (left) or intron 36 (right) of *ABCA4* by PCR. In each, the FL amplicon and the expected edited bands (E1 or E4) after the treatment with the selected gRNAs (gRNAs 30 1 + 2 for intron 30 of *ABCA4* and gRNAs 36 1 + 2 for intron 36 of *ABCA4*, respectively) were indicated on the right edge of the gel. The 5′ UTR of *RPE65* was amplified as loading control. (Below) A graph chart

(legend continued on next page)

specific analysis and determination of the PE transcript by RT-PCR, a statistically significant decrease of the PE-containing transcripts was detected compared to the NT control, both with and without CHX treatment prior to harvesting (Figure 8B). Considering both conditions (with and without CHX treatment), there was an ∼50% reduction in transcripts containing the PE. We confirmed this observation by qPCR in which we detected a significant reduction of those transcripts when comparing NT and RPNC conditions after CHX treatment (Figure 8C). These results therefore confirmed that the genome editing observed could significantly reduce the insertion of the PE in mRNA transcripts. Using RNA analysis, the quality of the PPC differentiation was characterized by assessing the expression of several retinal markers. The results showed a similar pattern to that observed in previous differentiations (Figure S10).[56,59,60]

Considering the observed reduction of the PE in the *ABCA4* transcripts at RNA levels, we further investigated the protein expression levels of ABCA4 in PPCs following 12 days of RPNC with gRNAs 30 1 + 2 delivery (Figure S11). Notably, neither the isogenic control nor the patient-derived PPCs exhibited discernible differences in ABCA4 protein expression between the NT conditions and samples treated with the RPNC, regardless of the presence or absence of gRNAs.

On- and off-target analysis revealed no off-target effects and the correct cleavage of intron 30

Upon confirming intron-30 scission by DNA analysis and decrease of PE insertion by RNA analysis, we proceeded with the evaluation of possible off-target effects caused by RPNCs in combination with the pair of gRNAs 30 1 + 2. To achieve this, we combined whole-genome sequencing (WGS) and targeted PacBio analysis.

Particularly, DNA samples for all replicates of both the NT PPCs and RPNC (gRNA pair 30 1 + 2) transfected samples (CHX−) were analyzed by WGS. The resulting data were filtered, and the output sequence was analyzed in the potential off-target areas based on on-line predictions. For the gRNA 30-1, none of the off-target potential areas (Table S4) present alterations that could be directly related to the transfection of the gRNAs (summary in Figures S12 and S13). Similarly, the off-target analysis on gRNA 30-2 also did not reveal any undesired effect that can be related to the gRNA transfection (summary in Figure S13).

Furthermore, to assess the on-target effect of such a big deletion, we employed long-read PacBio targeted sequencing. For that, the editing efficacy was analyzed in NT cells and edited cells; samples were not subjected to CHX treatment prior to harvesting. The analysis of the sequencing files clearly confirmed a partial deletion of the intron 30 of *ABCA4* in approximately 30% of the reads in both control and patient samples (Figure S14). Furthermore, we analyzed the editing junctions (Figure S15) and established a repair profile after partial intron 30 removal using RPNC with the gRNA pair 30 1 + 2 (Figure S16). In total we identified 44 editing events present in the isogenic control and/or the patient PPCs (Table S5). Among them, the top three sequences accounted for ∼82% of all reads and were shared between isogenic control and patient edited PPCs, with almost identical ratios (Figure S16; Table S5). Altogether, these analyses confirm that the gRNAs employed in PPCs were efficacious and specific, as no undesired effects were observed, making them attractive to be employed as a therapeutic approach for STGD1 cases caused by pathogenic variants of intron 30 of the *ABCA4* gene.

DISCUSSION

The utilization of CRISPR-Cas9 in genome editing presents significant promise for scientific research and medical applications.[6,61–70] However, the efficient delivery of CRISPR-Cas9 components into cells remains a challenge crucial for successful genome editing. Previous nonviral delivery systems, such as lipid-based nanoparticles,[30,32,33,71] gold nanoparticles,[72,73] DNA nanoclews,[43] polymer-based particles,[34,35] and extracellular vesicles,[63,74] while explored extensively, have limitations, including toxicity, reduced editing efficiency, complex formulation, and instability.

In our research, we identified the amphipathic peptide, LAH5, as a particularly efficient transporter for the delivery of ribonucleoprotein complexes (RNPs) across various cell lines.[50] In addition, we demonstrated that complexation and co-delivery of a single-stranded DNA (ssDNA) homology-directed repair (HDR) template with Cas9 RNP using the LAH5 peptide as transfection reagent exhibited efficient gene correction via HDR in a reporter cell line.[50] Nonetheless, it is pivotal to underscore that these levels of gene correction were achieved within transfection conditions characterized by reduced serum content in the culture medium. To enhance the resilience of the peptide against factors originating from serum and other potentially adverse influences, a frequently employed approach entails conjugating the peptide to a fatty acid moiety, typically in the form of a compact lipid tail.[51–53] In addition, recent investigations have introduced a range of delivery methods employing peptides modified with fatty acids (known as lipopeptides). In these studies, proficient delivery of RNPs across various cell types was demonstrated.[46–48] Therefore, in this work, we used oleic acid (C18:1)-modified LAH5 peptide (C18:1-LAH5). We showed that the RPNCs formulated with the C18:1-LAH5 lipopeptide outperformed LAH5-formulated

representing the overall result indicating the percentage of the FL amplicon and of the edited band (E) for each condition. Each bar is represented by mean ± SD (n = 3). (B) On the left, a representative electrophoresis gel of the amplification of intron 30 of *ABCA4* by PCR in patient-derived PPCs. FL amplicon and edited (E1) band were indicated at the right side of the gel. The 5′ UTR of *RPE65* was amplified as loading control. On the right, a graph bar representing the average result in which each bar is represented by the mean ± SD (n = 3 for all conditions, except gRNAs 30 1 + 2 CHX+, n = 4). MQ indicates the negative control of the PCR; NT, non-treated cells; P + C, cells treated with lipopeptide and Cas9 RNP but not with gRNAs; Pept, cells treated only with lipopeptide; Cas9, cells treated only with Cas9 RNP. CHX indicates if cell were treated with (+) or without (−) cycloheximide 24 h before harvesting. In all graph bars, a statistical significance with respect to the untreated condition (NT+) is indicated as *$p < 0.05$, **$p < 0.01$, ***$p < 0.001$, and ****$p < 0.0001$ using one-way ANOVA followed by Bonferroni correction.

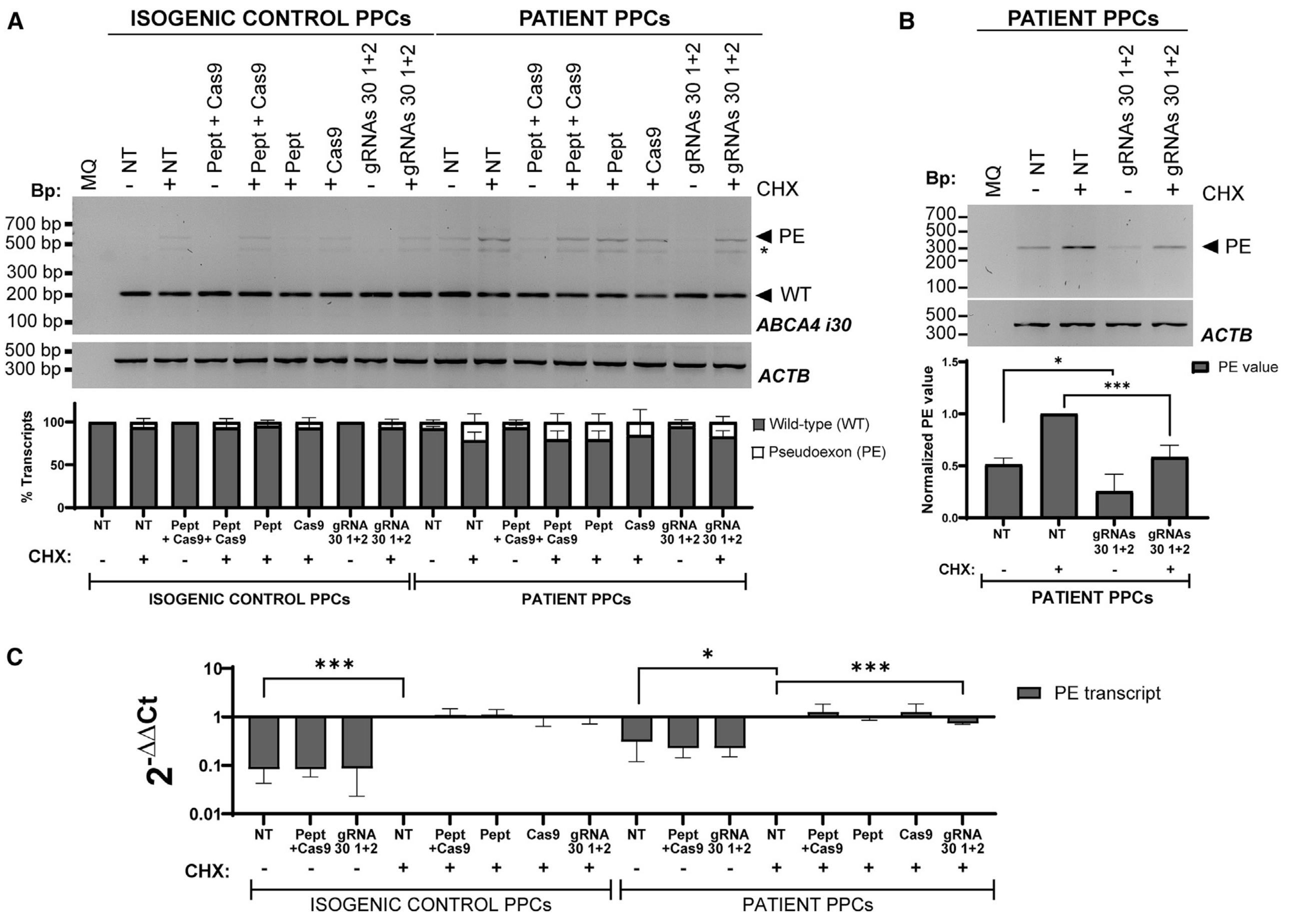

Figure 8. Analysis of the effect of the genome editing of intron 30 of *ABCA4* gene in PPCs at the RNA level

(A) RT-PCR from exon 30 to exon 31 of *ABCA4* ($n = 3$ for all conditions, except for gRNAs 1 + 2 CHX+, $n = 4$). (Top) A representative electrophoresis gel of the RT-PCR, below the bar graph showing the normalized PE-containing transcripts in comparison with the NT + condition of control or patient fibroblast. Each bar represents the mean +SD. (B) RT-PCR specifically amplifying the PE ($n = 3$ with the exception of gRNAs 30 1 + 2 CHX+, $n = 4$). (Top) A representative electrophoresis gel of a specific RT-PCR that specifically amplified the PE in patient PPCs ($n = 3$) is shown. (Bottom) The quantification of the transcripts was normalized against *ACTB* and the NT + condition (which is set to 1). (A and B) *ACTB* was amplified as loading control; MQ indicates the negative control of the PCR. CHX indicates if cell were treated with (+) or without (–) cycloheximide (CHX) 24 h prior to harvesting. Statistical significance with respect to the corresponding untreated condition (NT) is indicated. $^*p < 0.05$ or $^{***}p < 0.001$ (one-way ANOVA followed by Bonferroni correction). (C) qPCR analysis directly amplifying the PE in the control and patient line ($n = 3$ with exception of samples for gRNAs 30 1 + 2 whose $n = 4$). Each condition was normalized against *GUSB* and then compared with the NT+ condition of each cell line. (A–C) CHX indicates if cells were treated with (+) or without (–) CHX 24 h prior to harvesting. Statistical significance with respect to the untreated condition (NT+) is indicated as $^*p < 0.05$ or $^{***}p < 0.001$ by t test.

counterparts at RNP/peptide ratios of 1:150 and 1:250 in multiple cell types (Figures 4B and 4D). The ability of C18:1-LAH5 to form RPNCs with high capacity for encapsulating Cas9 protein and gRNA is shown in Figures 2B–2D, 5D, and S5C. Our interest extended to evaluating its enhanced stability and superior performance across various ratios and reporter cell types. This was affirmed through gene-editing efficiency studies in HEK293T and HEPA 1–6 cells (Figures 4B and 4D), aligning with existing literature.[46–48] Collectively, these findings firmly establish the superiority of C18:1-LAH5 over LAH5 as a vector for delivering Cas9 RNP.

Based on these results, we then tested the C18:1-LAH5 lipopeptide to deliver Cas9 RNP into different cell models to assess the suitability of genome editing as an approach to correct the effect caused by DI variants in the *ABCA4* gene. Currently, the use of AONs is the most explored treatment approach to correct the splicing defects caused by DI variants, especially in large genes such as *ABCA4* or *USH2A*, in which gene-augmentation therapy is not feasible with a single adeno-associated virus (AAV) vector.[75] However, the main limitation of using AONs to treat these splicing defects is that the molecules are mainly variant specific, limiting the number of patients that can be treated with a single therapeutic molecule. In addition, several intravitreal injections are required per year. Removing a large intron section by genome-editing approaches, as shown in this work, increases the number of patients eligible for the same therapeutic approach. In addition, because CRISPR-Cas9 acts at the DNA level, the correction

can be permanent, potentially allowing for single-dose treatment. As we sought to maximize the performance of the C18:1-LAH5 lipopeptide by using two gRNAs flanking the intron region to be excised, we needed to fully evaluate its capabilities. A critical aspect was to confirm that C18:1-LAH5 could successfully deliver Cas9 protein and two gRNAs simultaneously into the cytosol of target cells. The results derived from our live-cell confocal imaging investigations provide strong confirmation for this approach (Figures 5E and S5). Considering the robust interaction observed between the C18:1-LAH5 lipopeptide and the Cas9 RNP (Figure 5B), alongside its notable capability for RPNC formation (Figures 5D, S5B, and S5C) and its favorable safety profile (Figures S3A–S3C), this compound emerges as a promising candidate for the therapeutic strategy we had envisioned.

We further validated that RPNC is indeed a potent delivery vehicle by demonstrating the translational possibilities by assessing the editing in control and patient-derived fibroblasts and PPCs. However, the correction effect at RNA level (i.e., decrease of PE insertion) after genome editing was only clearly detectable in the PPC model and not in the fibroblast cells. This is probably because *ABCA4* mRNA levels were still very low even after trans-differentiation and treatment with CHX. To boost that expression but also increase the PE detection (as we have previously shown that, in regular fibroblasts, the PE is not detected),[14] we used a small-molecule-based approach adapted from a viral delivery trans-differentiation protocol previously described.[76] However, in our hands, the viral delivery of the different transcription factors did not induce any effect, but, by only replacing the medium with the different small molecules, *ABCA4* expression as well as the detection of the PE increased. Still, these levels are relatively low compared to other cellular systems such as PPCs or retinal organoids. Therefore, the lack of conclusive results at RNA level might be related to the limit of detection of the overall approach.

When we focused on the results obtained at the DNA level in the patient PPC line, we demonstrated that the editing efficiencies were consistent independently of the method (PacBio sequencing or agarose gel) we employed. In PPCs, an editing efficiency of ∼35%–47% led to a reduction of ∼50% of PE-containing transcripts (considering both conditions, with and without CHX treatment, respectively), suggesting a good correlation between DNA editing and reduction of transcripts containing the PE at the RNA level. One could think whether this would be enough for therapeutic rescue. Modeling studies have predicted that ∼30%–35% of ABCA4 function is needed to have normal function and therefore not cause STGD1.[77] Based on the ∼38%–50% observed transcripts without PE, PIR of one allele could reach at least 20%–25% rescue. However, one of the risks of removing big pieces of introns is to indirectly remove important splice enhancers or silencers that can affect the splicing pattern and overall *ABCA4* expression levels. We observed that the splicing pattern in our RT-PCR analysis from exon 30 to exon 31 of ABCA4 did not present any new transcripts after editing (Figures 8A and S8A), but we also evaluated the expression of *ABCA4* mRNA by qPCR (Figure S17). Our findings suggest an increase in the wild-type transcript following RPNC treatment in patient PPCs. However, this increase did not reach statistical significance, likely because the other allele produces high levels of this transcript. In addition, we did not observe any differences in the expression levels between conditions in the isogenic control PPCs, suggesting overall that the PIR of intron 30 did not affect the splicing process.

In addition, ABCA4 protein has a relatively long half-life,[78] which could also contribute to reaching the therapeutic threshold within the current editing values. Our western blot analysis revealed the presence of ABCA4 along with some additional bands, similar to those we have previously observed in PPC or fibroblast models. Here, these bands may be due to the heterogeneity of the PPC model. Nevertheless, their significance remains unclear to us. Despite this, we quantified both the ABCA4 band and these additional bands, and neither showed an increased level after RPNC treatment. As previously demonstrated by our group, assessing ABCA4 protein levels poses challenges, particularly in premature and heterogeneous models such as the PPCs utilized in this study.[79] In this case, it is even more challenging to observe an increase in ABCA4 protein levels, as the patient lines are compound heterozygous, having one allele carrying the c.4539+2001G>A and the other one a c.4892T>C missense variant, which is still able to produce normal levels of non-functional protein. While, for RNA analysis, CHX treatment is employed to block NMD and increase the differences between normal and PE-containing transcripts, for protein this is not possible as CHX inhibits protein synthesis too. Therefore, it remains challenging to correlate the levels of editing and PE-reduction with protein increase and further investigations in more complex models (e.g., retinal organoids) are required.

Furthermore, it has been previously shown that delivery of CRISPR systems to patient cells can rescue ABCA4 function. For example, using prime editing, variants in *ABCA4* could be corrected at iPSC level.[80] However, we aimed to correct the splicing defect in differentiated cells, as this is more similar to a potential therapeutic intervention in patients. De Angeli et al. also delivered the Cas9 plasmid to PPCs by nucleofection with high efficacy.[81] Unfortunately, as we will further discuss later in this section, this delivery method is also not appropriate for patients. Another alternative is to employ AAV to deliver *ABCA4* directly to patients. However, the cargo capacity of 5 kb of the AAV is quite limiting for large genes such as *ABCA4*. To circumvent this limitation, a dual-AAV genome-augmentation approach for *ABCA4* has been explored in different ways.[82–84] Despite a favorable safety profile observed so far, this delivery method still faces challenges such as the risk of unrecombined 5′-3′ transgenes, the potential high dose required for reaching the therapeutic threshold, and the reduction of the episomes over time.[85] Alternatively, we decided to create nanocomplexes using a conjugating peptide that could allow us to assess whether large removal of the intron could have deleterious effects on splicing but at the same time evaluate PE removal. Our proof-of-concept studies showed that this system is suitable to deliver to differentiated cells and verify our

hypothesis. In order to increase the editing capacity, further optimization of this RPNC will be needed; however, it is also important to verify that the C18:1-LAH5 lipopeptide will be safe for delivery to the retina. Other alternative methods to deliver this system could be, as mentioned above, the use of AAVs as used in a clinical trial for *CEP290* (NCT03872479),[86] or Cas9 delivered as mRNA in a nanoparticle, as previously shown for liver delivery in patients with transthyretin amyloidosis.[87]

Moreover, the PPC model, despite being retina specific, consists of a mixed cell population that is not well defined, and therefore this can lead to variability in Cas9 RNP uptake and *ABCA4* expression among different cell lines and batches. This diversity may result in varying editing efficiencies among individual cells,[88] which may lead to lower editing ratio while the total DNA was analyzed. Moreover, it is important to note that NMD is not universally 100% effective in all cell types,[89] which could potentially lead to either underestimation or overestimation when translating observations from DNA to RNA levels. Exploration on more complex models may contribute to enhancing the efficacy values observed in this manuscript.

To our knowledge, only three published studies have reported genome editing for treating STGD1 *ABCA4* variants.[80,81,90] The first and last studies were published by De Angeli et al., in 2022 and 2024, respectively. In both, the authors investigated the genome editing of DI variants in intron 36 in PPCs.[81] The second study published in 2023 looked at the genome editing for different *ABCA4* variants in hiPSCs.[80] However, a common feature of both studies is the use of electroporation as a delivery method for either gRNAs and Cas9 RNP[80] or for a plasmid containing both.[81] This method involves applying a localized and brief external electrical pulse to modify the permeability of cell membranes.[91] In the eye, this method has been used for the transduction of retinal cells in newborn rodents[92] and also in adult animals.[93,94] Although the results of these *in vivo* applications are promising, electroporation requires an invasive surgical procedure in order to place the microelectrodes that generate the localized electrical pulse, which makes its translation to humans more difficult. In fact, the focus of current clinical trials with electroporation-based technology (EYS606,[95] Eyevensys, Paris, France) is on the anterior segment of the eye (NCT04207983 and NCT03308045). In addition to the aforementioned studies, Botond Roska's group employed an adenine-based editing approach to correct the prevalent *ABCA4* c.5882G>A variant using AAV for CRISPR-Cas9 delivery.[96] The reported editing efficiencies ranged from 5% to 15% in retinal organoids to 10%20% in human retina explants. However, only preliminary findings presented in a conference communication (B. Gyorgy et al., 2023, ARVO, conference) are available, as the full study has not yet been published.

Given this context, it is not surprising that the sole clinical trial for direct ocular genome editing (NCT03872479) was performed through a subretinal injection. This groundbreaking trial specifically aimed to remove part of intron 26 of the *CEP290* gene in patients with Leber congenital amaurosis (LCA).[97,98] The recently published trial results showed that some patients had meaningful visual improvement, but further research is required.[99] Furthermore, the recent approval of the first CRISPR-Cas-based therapeutic approach in the United Kingdom emphasizes the significance of advancing such strategies to unlock new therapeutic possibilities.[100] This broader context has motivated our exploration of alternative delivery systems, including the investigation of cell-penetrating peptides, aimed at bringing CRISPR-based approaches one step closer to clinical trials.

In addition to an easier translational delivery approach, using a cell-penetrating peptide as a carrier for Cas9 RNP offers other advantages. First, the amphipathic properties of the C18:1-LAH5 enables supramolecular complexation involving both electrostatic and hydrophobic interactions. Furthermore, the overall positive charge of such peptide nanocomplexes facilitates non-specific binding to cell membranes. This enables efficient cellular uptake and endosomal escape, overcoming a common challenge faced by nanoparticle- and polymer-based delivery systems.[101] Furthermore, it is important to note that previous research has demonstrated that fatty acid modification of cell-penetrating peptides enhances transfection efficiency. This enhancement is primarily due to increased membrane binding and uptake. The protonation of the multiple histidine residues in the LAH5 peptide within the acidic environment of early and late endosomes subsequently promotes endosomal escape.[51,102] Additionally, fatty acid modification has been shown to increase the stability of peptides, making them less susceptible to enzymatic degradation.[51] Altogether, these properties give us confidence that this peptide vector has promising potential for local eye delivery via intravitreal or subretinal injections. However, *in vivo* studies are required to further study its potential and are currently planned to provide proof of concept for direct *in vivo* gene correction to treat STGD1.

Overall, this work provides a safe and effective delivery method for RNPs formed with Cas9 and dual gRNAs utilizing C18:1-LAH5 lipopeptide. We validated this system as an approach for correcting multiple splicing defects caused by DI variants in either intron 30 or intron 36 of the *ABCA4* gene. These findings pave the way for new promising avenues to further develop potential treatments for STGD1 and to deliver genome-editing complexes to the eye, which can also be applied for other retinal diseases.

MATERIALS AND METHODS

General reagents for peptide analysis and characterization

All chemicals and substances employed in the peptide analysis and characterization were purchased from Merck (Saint Louis, MO, USA) unless otherwise specified. LAH5 peptide and C18:1-LAH5 lipopeptide were obtained from SynPeptide (Shanghai, China). gRNA sequences were purchased from Merck (Haverhill, UK). tracrRNA-ATTO550, tracrRNA-ATTO647, tracrRNA-ATTO488, crRNA sequences, and PCR primers were purchased from Integrated DNA Technologies (Iowa, USA) (sequences are listed in Table S2), and spCas9 and GFP-Cas9 were obtained from Merck (Saint Louis, MO, USA). OptiMEM I (1×) was acquired from Gibco (Waltham, MA, USA), Lipofectamine, CRISPRMAX Transfection Reagent,

Lysotracker Green DND-26, and Lysotracker Red DND-99 were acquired from Invitrogen (Thermo Fisher Scientific, Vilnius, Lithuania). T7 endonuclease and Q5 high fidelity 2× master mix were purchased from New England Biolabs (Ipswich, MA, USA). DNA extraction and PCR purification kits were purchased from QIAGEN Benelux (Venlo, the Netherlands). TAE buffer was purchased from Bio-Rad (Hercules, CA, USA).

RPNCs for gene editing and PIR

To formulate RPNCs for gene editing, gRNA, and Cas9 protein were combined at a 1:1 molar ratio. This was done either in 10 mM HEPES buffer at pH 7.4 or in OptiMEM (used for transfection). The mixture was then incubated at room temperature for 10 min to form the RNP complex. Subsequently, this RNP complex was mixed with the C18:1-LAH5 lipopeptide at various RNP:C18:1-LAH5 molar ratios (1:50, 1:100, 1:150, 1:200, and 1:250). This was followed by a further 10-min incubation at room temperature. The RPNC (RNP/C18:1-LAH5) mixture was then further diluted with 10 mM HEPES buffer at pH 7.4 for characterization experiments and OptiMEM for cellular experiments. This dilution gave a final concentration of 20 nM for the RNP and a concentration of the C18:1-LAH5 lipopeptide in the range of 1–5 μM. The formulation of the RPNCs for PIR was conducted in the same way, using the same buffer as specified for gene editing. The distinction in this formulation lies in the composition of the RNPs, which was developed in the presence of two different gRNAs. To form the RPNCs three different molar ratios were generated: Cas9:gRNA:C18:1-LAH5 at 1:2:50, 1:2:150, and 1:2:250, respectively.

Physical characterization of RPNCs

RPNCs with one gRNA for gene editing were diluted with 10 mM HEPES buffer at pH 7.4, resulting in a final concentration of 20 nM for the RNP and C18:1-LAH5 lipopeptide concentrations ranging from 1 to 5 μM (RNP/lipopeptide). RPNCs for PIR, named Cas9/gRNA/C18:1-LAH5, were prepared at ratios varying from 1:2:50 to 1:2:250. These RPNCs were analyzed for size and PDI using DLS with a Zetasizer ZS instrument (Malvern, Worcestershire, UK). Subsequently, the samples were diluted with 10 mM HEPES buffer at pH 7.4, for ζ-potential assessment using a Zetasizer Nano Z ALV CGS-3 (Malvern, Worcestershire, UK). To determine the size and charge of the RPNCs at different lipopeptide concentrations, the sample was measured three times in each test condition. In addition, the formation of the RPNCs was determined by an EMSA.

EMSA

RPNC (Cas9-RNP/C18:1-LAH5) formation was determined by performing a fluorescence-based EMSA using a non-staining and non-denaturing 1.5% agarose gel. In this experiment, GFP-Cas9, ATTO550-gRNA, ATTO647-gRNA, and C18:1-LAH5 lipopeptides were used. The labeled components and the unlabeled C18:1-LAH5 lipopeptides were formulated to form RPNCs for gene editing and PIR at different molar ratios as previously described. All labeled elements were further diluted to yield a final concentration of 1 μM in water supplement with 2% of 40% glycerol solution. Visualization of the electrophoresis gel was done using a ChemiDoc XRS+ system (Bio-Rad). Fluorescent images were analyzed using the ImageJ version 1.53t Software.[103]

Cell culture

HEK293T cells (ATCC# CRL-3216), HEK293T stoplight cells,[54] and eGFP HEPA 1–6 cells[55,104] were cultured in Dulbecco's modified Eagle's medium (DMEM) (Thermo Fisher Scientific, Waltham, MA, USA) supplemented with 10% fetal calf serum (FCS). Fibroblasts were cultured in DMEM (Thermo Fisher Scientific) supplemented with 20% FCS. In addition, HEK293T and fibroblast culture media were additionally supplemented with 1% penicillin/streptomycin and 1% sodium pyruvate. HeLa (CCL-2) cells were cultured in low-glucose DMEM supplemented with 10% FBS. All cell lines were grown at 37°C and 5% CO_2. Cells were passed twice a week. For experiments, cells were harvested using trypsin after washing them with 1x PBS twice.

A skin biopsy from a patient carrying the *ABCA4* variants c.4539+2001G>A (p.?) and c.4892T>C (p.Leu1631Pro) was collected as described previously by the group.[14] Our research was conducted according to the tenets of the Declaration of Helsinki and the procedures to obtain the skin biopsy from the patient for establishing primary fibroblasts cultures and subsequent reprogramming into iPSCs were approved by the local Ethical Committee. Samples were collected upon written informed consent was gathered from the participant.

In vitro cytotoxicity assay

HEK293T stoplight,[54] HeLa, and eGFP HEPA 1–6 cells were cultured in a Greiner Bio-One 96-well plate at a density of 3 × 10^4 cells per well. After a 24-h incubation period, cells were transfected using a 100-μL solution of RPNC (RNP/C18:1-LAH5). These were prepared presence of 20 nM RNP and 1–20 μM lipopeptide and following at a ratio of RNP:lipopeptide ranging from 1:50 to 1:1,000. As a reference, positive-control wells were treated with a 100 μL of medium enriched with 5% Triton X-100. This experimental procedure was conducted in triplicate for each condition.

Twenty-four hours after transfection, cell viability was assessed by treating the cells with a 20-μL solution of CellTiter 96 Aqueous One solution cell proliferation assay (MTS) reagent (Promega Corporation, Madison, WI, USA), following the manufacturer's recommended protocol. Fluorescence intensity was quantified with an absorbance set of 490 nm using an IMark Microplate Reader (Bio-Rad). Data were normalized by excluding reference positive-control signals from negative-control and treated samples.

Cellular uptake of RPNCs for gene editing and PIR

HeLa cells were seeded at a density of 2 × 10^4 cells/well. The following day, the cells were treated with the prepared RPNC (RNP/C18:1-LAH5). To formulate RPNCs for gene editing, RNP complexes were prepared by mixing GFP-Cas9 RNP with *CCR5*-targeted sgRNA or Cas9 protein with ATTO550-gRNA and incubated for 10 min. Then, they were combined with C18:1-LAH5 lipopeptide at a 1:150

ratio and incubated for another 10 min prior to being added to the cells (20 nM RNP, 3 μM lipopeptide). After 24 h, the cells were treated with 5 mg/mL Hoechst 33342 together with 100 nM LysoTracker green DND-26 or LysoTracker red DND-99, all of which were added to the complete cell culture medium for a duration of 30 min. The cells were then washed with 1× PBS and imaged using a Yokogawa CV7000 Confocal Microscope (Yokogawa Corporation, Tokyo, Japan). Acquired images were standardized to brightness and contrast. ImageJ version 1.53t software[103] was used to quantify total relative fluorescence intensity. In the RNP-treated samples (RNP was delivered without lipopeptide) and those treated with RPNC (RNP/lipopeptide), the difference in fluorescence intensity between the component of interest and LysoTracker's the relative fluorescence intensity of LysoTracker was assessed. Relative mean fluorescence intensity (RMFI) was calculated as RMFI = fluorescence intensity of material − background intensity.

To assess the cellular uptake of RPNC for PIR, the experimental protocol was performed in an equivalent manner, with the only difference being the use of 40 nM Cas9, 40 nM gRNA1, 40 nM gRNA2, and 6 μM lipopeptide concentration to maintain a consistent molar ratio between RNP and lipopeptide. GFP-Cas9, ATTO550-gRNA1, and ATTO647-gRNA2 formed RNP mixed with C18:1-LAH5 lipopeptide (1:1:1:150 ratio). Cells were exposed to RPNCs at various times, and different combinations of RPNCs were used to validate cellular uptake.

Gene-editing efficiency of the RPNCs

HEK293T stoplight cells were cultured in a Greiner Bio-One 96-well plate) at 3 × 10^4 cells/well. After 24 h, cells were treated with RNP/LAH5 or RNP/C18:1-LAH5 RPNCs containing 20 nM RNP and peptides at ratios of 1:50 to 1:250. CRISPRMAX was used as a positive control according to the manufacturer's protocol. After 48 h, cells were harvested, transferred to Falcon U-Bottom 96-well plate (Becton Dickinson, USA, #353077), washed twice with 1× PBS, and fixed with 1% paraformaldehyde. Flow-cytometry analysis (mCherry and eGFP signals) was performed using the FACSCanto II instrument (Becton Dickinson, Franklin Lakes, NJ, USA). Acquired data were analyzed with Flowlogic software version 8.7 (INIVAI Technologies, Mentone, Victoria, Australia) and gated according to gating strategies shown in Figure S18. Gene-editing efficiency was determined by analyzing mCherry and eGFP expression, as previously described.[54]

Furthermore, gene-editing efficiency was assessed in eGFP HEPA 1–6 cells. They were seeded in a 96-well plate at 3 × 10^4 cells/well and treated 1 day later with RPNC (RNP [20 nM]/LAH5) or RPNC (RNP [20 nM]/C18:1-LAH5) (ratios: 1:50, 1:150, and 1:250). Three days later, cells were transferred to 24-well plates and expanded for two more days (a total of 5 days post transfection). The measurement of cell fluorescence was carried out using the BD FACSCanto II instrument, with the eGFP fluorescence signal being detected within the FITC channel. After data acquisition, analysis was executed employing the FlowLogic software FlowLogic Alias version 8.1 (INIVAI technologies). Gene editing was assessed by reduced green fluorescence intensity, following the gating strategy shown in Figure S19.

The gRNA sequences employed for the HEK293T stoplight and eGFP HEPA 1–6 cells reporter cell experiment were sgRNA. In contrast, the remaining gRNAs used in this study were a combination of transfer RNAs (tracRNAs) and CRISPRRNAs (crRNAs) and were specified as gRNAs.

Indel efficiency was evaluated by T7E1 assay. Nano-Flow cytometry (NanoFCM) was also used to assess the correct formulation of RPNC for gene editing and PIR.

T7 endonuclease assay

T7E1 assay was performed on HeLa cells to demonstrate indel efficiency. After 48 h of transfection with RPNC (GFP-Cas9/CCR5 gRNA/C18:1-LAH5), genomic DNA was extracted using the QIAGEN DNeasy Blood & Tissue Kit (QIAGEN Benelux). Primers specifically designed for the *CCR5* region (Table S6) were used for PCR amplification employing Q5 Hot Start High-Fidelity 2X Master Mix (New England Biolabs, Ipswich, MA, USA), according to the manufacturer's instructions. The resulting PCR products were denatured in NEBuffer 2 (New England Biolabs) at 95°C for 10 min, followed by gradual annealing through a temperature-reduction process (from 95°C to 85°C at a rate of 2°C per second, and from 85°C to 25°C at a rate of 1°C per second). The heteroduplex DNA sequences were then exposed to the T7E1 enzyme (New England Biolabs) for 15 min at 37°C to facilitate the cleavage of DNA mismatches.

The indel frequency in the DNA sequences was calculated using the equation %indel = (1 − (1 − (b + c)/(a + b + c))1/2) × 100, where *a* represents the band intensity of the DNA substrate, and *b* and *c* represent the intensities of the cleavage products.[105]

NanoFCM

RPNCs for gene editing and for PIR were analyzed using NanoFCM (NanoFCM). RPNCs were formed by combining GFP Cas9/ATTO647-gRNA with C18:1-LAH5 peptide at a ratio of 1:150. RPNCs for PIR contained unlabeled Cas9/ATTO488-gRNA1/ATTO647-gRNA2, with C18:1-LAH5 peptide at a fixed Cas9:gRNA:-peptide ratio of 1:2:150.

NanoFCM measurements were done using a NanoAnalyzer U30 instrument with dual 488/640-nm lasers and single-photon counting avalanche photodiode detectors (SPCM APDs). SSC and fluorescence signals were measured (488/10 for SSC, 525/40 for FL1, 670/30 for FL2). HPLC-grade water was used for sheath fluid control, maintaining a core stream diameter of 1.4 μm. Measurements were taken for 1 min at 1.0-kPa pressure. To achieve a flow of 2,000–12,000 particles per minute, samples were diluted in TE buffer. Particle concentrations were determined using a 250-nm silica nanoparticles standard (NanoFCM, Nottingham, UK). Particle sizes were evaluated using a four-modal silica nanosphere cocktail (68, 91, 113, and 155 nm) (NanoFCM).

Data processing was performed using NanoFCM Profession V2.0 software (NanoFCM). The resulting visual report allowed the analysis of subpopulations based on fluorescence. Size distribution and

concentration information was provided for each sub-population. Additional data showing intensity values for individual events were exported for the calculation of mean fluorescence intensity (MFI), as shown in the supplemental information (Table S3).

gRNA design and selection

gRNA sequences were designed employing two different web tools, CRISPOR (http://crispor.tefor.net/) and CHOPCHOP (https://chopchop.cbu.uib.no/), using intron 30 and intron 36 of *ABCA4* gene as a target sequence. The most promising sequences from the output were selected based on ranking and considering the predicted efficacy and off targets. In total, 18 gRNAs were selected (Table S2) and amplified from the *ABCA4* gene template (primers listed on Table S7) to be further cloned into pX458 (Addgene plasmid #48138; http://n2t.net/addgene:48138; RRID:Addgene_48138) for initial evaluation in HEK293T cells. Transfection of pX458 with different combinations of sgRNAs were tested based on their upstream or downstream position from the hot-spot cluster. The best combinations of gRNAs were subsequently ordered as crRNA for further evaluation using the Cas9 RNP system.

Photoreceptor precursor cell differentiation and characterization

iPSC lines were previously generated by the Radboudumc Stem Cell Technology Center and characterized elsewhere.[14,57,58] The differentiation from iPSCs to PPCs was conducted by adapting the protocols published by González-Cordero et al. in 2017 and Capowsky et al. in 2019.[106,107] For the differentiation to PPCs, iPSCs were seeded as single cells and maintained in E8F medium (Gibco, # A2858501) until becoming confluent. At that point, cells were grown in E6 medium (Gibco, # A1516401) for 2 days. Subsequently, medium was changed to Neural Induction Medium (NIM, consisting of Advanced DMEM/F12 [Thermo Fisher Scientific] supplemented with 1% N2 supplement [Gibco, # 17502048], 100 mM GlutaMAX [Gibco, # 35050038], and 1% penicillin/streptomycin). NIM medium was changed every other day. On day 6, 1.5 nM BMP4 (R&D Systems, Minneapolis, MN, USA) was added to the NIM medium. After the BMP4 pulse, half of the NIM medium was refreshed every other day until the end of the differentiation (day 30). All PPC conditions were harvested by scraping the cells after washing the wells with 1× PBS. Experiments were performed in two independent differentiation batches. Further details about the RPNC delivery and analysis are explained in the next section.

To characterize the differentiation process, RNA from day 0 and day 30 of differentiation was analyzed. In total, 1 μg of RNA was used to synthetize cDNA by using the SuperScript VILO Master Mix (Thermo Fisher Scientific) according to the manufacturer's instructions. Then, a qPCR analysis was performed with the GoTaq Real-Time Quantitative PCR Master kit (Promega). The reactions were performed in triplicates using the Applied Biosystem QuantStudio 5 Digital system (Applied Biosystem, Waltham, MA, USA). The expression levels of eight markers (*OCT3/4*, *OTX2*, *CRX*, *RECOVERIN*, *PAX6*, *NRL*, *OPN1SW*, *ABCA4*, and *RPE65*) and a housekeeping gene (*GUSB*) were studied to evaluate the success of the differentiation. Each sample was normalized against the expression of the housekeeping gene and compared with iPSCs (day 0) using the $2^{-(\Delta\Delta Ct)}$ method.[108] The complete list of primers is provided in Table S8.

RPNC delivery for PIR in fibroblast and PPCs

Two fibroblast lines and iPSCs for PPC generation were employed, originating from two individuals—one from a healthy control and the other from a patient with the heterozygous c.4539+2001G>A DI variant in intron 30 of *ABCA4*. The PPCs were obtained following the procedure outlined the previous section, and the identical protocol was applied for the delivery of RPNCs in both cell types.

gRNAs were delivered as a crRNA-tracrRNA complex (IDT, Coralville, IA, USA). To this end, gRNAs sequences were synthetized as crRNA and diluted in duplex buffer to a final concentration of 200 μM. Then, the crRNA:tracrRNA duplex was assembled by mixing the two RNA oligos in 1:1 equimolar concentration together with duplex buffer to reach a final duplex concentration of 50 μM.

For the lipopeptide-mediated delivery, the crRNA:tracrRNA (gRNAs) was first diluted to a final concentration of 2.5 μM in nuclease-free water. Then Cas9 ribonucleoprotein (RNP) (Sigma, Saint Louis, MO, USA) stock was reconstituted and further diluted to a final concentration of 1,530 nM. For cellular delivery, the stock was diluted 2.5 times in OptiMEM (Sigma) and mixed together with the crRNA:tracrRNA complex at 2.5 μM (per gRNA) for 15 min at room temperature to form RNP. Consequently, the resulting RNP ratios were equal to Cas9 (25 nM)/gRNA1 (25 nM) and gRNA2 (25nM) at a 1:2 proportion, within a total 1-mL volume in a six-well reaction plate. Lipopeptide stock was diluted in nuclease-free water with 1% of acetic acid to enhance solubility. Subsequently, dilutions were prepared with OptiMEM (Gibco). The C:18-1/LAH5 lipopeptide was prepared to a final concentration of 6 μM, within a total 1-mL volume in a six-well reaction plate. Then, the C:18-1/LAH5 lipopeptide was thoroughly mixed with the gRNAs-Cas9 RNP solution and incubated together for 20 min at room temperature. This process resulted in the creation of RPNC for PIR with determined molar ratios. Finally, these RPNCs were supplemented to the cells. Of note, to be able to detect the PE, samples for RNA analysis were treated with CHX 24 h before harvesting.

The optimal concentration of lipopeptide was determined by a dose-response experiment that was conducted using increasing concentrations of the C18:1-LAH5 lipopeptide. These concentrations ranged from 1 μM (1:2:25) to 12 μM (1:2:300) or from 3 μM (1:2:75) to 30 μM (1:2:750) when considering the RPNC (Cas9:gRNAs:C18:1-LAH5) molar ratios. Fibroblast cells were subjected to RPNC for PIR delivery using concentrations ranging from 1 μM (1:2:25) to 12 μM (1:2:300), while PPCs were treated with concentrations ranging from 3 μM (1:2:75) to 30 μM (1:2:750). For the treatment of PPCs, RPNCs were delivered on day 20–22 PPCs, and samples were collected on day 30 or 31 for DNA and RNA analysis. For protein studies, PPCs were harvested on day 38–39. For fibroblast treatment, RPNCs were delivered the day after seeding 200,000 cells in a six-well plate. After RPNC delivery, fibroblasts were subsequently trans-differentiated to

boost the expression of *ABCA4*, as explained in the next section. In both cellular models, the same wells were split into two to perform DNA and RNA analysis from the same batch of cells, directly correlating DNA editing with its effect at the RNA level.

Fibroblast trans-differentiation

After lipopeptide-mediated delivery of the Cas9 RNP, fibroblasts were trans-differentiated over 10 days using a method adapted from Seko et al.[76] For this, the culture medium was replaced by DMEM/Ham's F-12 Nutrient Mix medium (1:1) (Thermo Fisher Scientific) supplemented with 1% FCS and 0.2% primocin. Prior to each addition, medium was supplemented with 40 ng/mL bFGF, 20 ng/mL EGF, and 1× fibronectin and B27. The medium was changed every other day until the end of the trans-differentiation.

DNA isolation and genome-editing analysis

DNA isolation was conducted using the QIAmp DNA Mini Kit (QIAGEN Benelux) according to the manufacturer's instructions. A total of 40 ng of the obtained DNA was employed for the PCR to amplify intron 30 or intron 36. The 5′ UTR of *RPE65* was employed as a loading control. PCRs were conducted using Ampli TaqGold 360 mix (Applied Biosystems) mixed with the corresponding primers (Table S7) at a concentration of 10 μM. The PCR program included a denaturation step of 94°C for 10 min, followed by 35 cycles of melting (94°C for 30 s), annealing (58°C for 30 s), and extension (72°C for 2 min and 30 s) steps, with a final elongation step of 72°C for 5 min. Finally, the gel-based semi-quantification of the different transcripts was performed using ImageJ version 1.53t software.[103] The information regarding the whole-genome sequencing (WGS) and PacBio analysis is explained in the supplemental information.

RNA isolation and splicing correction analysis

RNA isolation was isolated using the Nucleospin RNA kit (Machery Nagel Duren, Germany) according to the manufacturer's instructions. Total RNA concentrations were measured using Nanodrop 2000 and 1 μg of total RNA was used for cDNA synthesis using the VILO superscript IV cDNA synthesis kit (Thermo Fisher Scientific) following the manufacturer's recommendations. Next, 50 ng of cDNA was used as a template to amplify the PE of intron 30. As a loading control, *ACTB* was also amplified. A complete list of primers can be found in Table S7. All PCR mixtures contained 1× PCR buffer with $MgCl_2$ (Roche, Manheim, Germany), 1× Q-solution (QIAGEN Benelux), 2.5 mM $MgCl_2$, 2 μM dNTPs, 0.2 mM each primer, and 0.5 U Taq DNA Polymerase (Roche). The PCR program followed was 94°C for 5 min, followed by 35 cycles of melting (94°C for 30 s), annealing (56°C for 30 s), and extension (72°C for 1 min for transcript <1,000 nt or 2 min for transcript >1,000 nt) steps, with a final elongation step of 72°C for 10 min. PCR products were resolved by electrophoresis and all the transcripts were verified by Sanger sequencing. Semiquantitative gel-based analysis was conducted using ImageJ version 1.53t software.[103]

Statistics

GraphPad Prism 10 software (GraphPad, San Diego, CA, USA) was used for graph generation and statistical analysis. For visualization, all assays were presented as mean ± standard deviation (SD). A two tailed unpaired t test was used to statistically compare RMFI differences between non-peptide and peptide-treated samples. Differences in the assays related to the C18:LAH5 peptide analysis were determined by using one-way ANOVA Tukey's multiple comparisons test in Figures 4B and 4D only specified the *p* values illustrated in the figures. To evaluate the differences with respect to the isogenic control samples, the comparison was evaluated using a one-way ANOVA test followed by the Bonferroni correction. In all cases, a *p* value of 0.05 or less was considered statistically significant, and each experiment was performed in triplicate unless otherwise stated.

DATA AND CODE AVAILABILITY

The data supporting the findings described in this work are available within the article and the supplemental information. Raw data are available from the corresponding authors upon request.

ACKNOWLEDGMENTS

We want to thank Michael Kwint for his guidance in the preparation of the samples for PacBio analysis. We also acknowledge Ronny Derks for his help in the off-target and on-target analyses, respectively. We also want to thank Dr. Brooke L. Latour for reviewing the manuscript.

This research was partially funded by the Netherlands Organisation for Scientific Research (NWO) Talent program VICI, grant number 867.17.005 (to E.M.) and by Uit-Zicht, grant number UZ2019-17 (to A.G.). M.Ö. was supported by the PhD scholarship provided by the Republic of Türkiye Ministry of National Education. The funding organizations provided unrestricted grants and had no role in the design or conduct of this research. Graphical abstract; Figures 1A, 3A, 3C, and 5; and the RNP and RPNC in Figures 4C and 4D were created with BioRender.com.

AUTHOR CONTRIBUTIONS

I.V.-D., conceptualization, formal analysis, investigation, methodology, supervision, validation, visualization, writing – original draft, and writing – review & editing; M.Ö., conceptualization, formal analysis, investigation, methodology, validation, visualization, writing – original draft, and writing –review & editing; F.A.W., investigation, methodology, and writing – review & editing; T.H.N., investigation, methodology, and writing – review & editing; A.D.M.H., investigation, methodology, and writing – review & editing; E.R., investigation, methodology, and writing – review & editing; G.D.N.A., formal analysis, and writing – review & editing; R.T., formal analysis and writing –review & editing; N.S.-H., investigation, methodology, and writing – review & editing; I.B., investigation, methodology, and writing – review & editing; A.R.-L., investigation, methodology, and writing – review & editing; J.B., formal analysis and methodology; O.G.d.J., supervision, validation, and writing – review & editing; R.W.J.C., conceptualization, funding acquisition, resources, supervision, validation, and writing – review & editing; E.M., conceptualization, funding acquisition, project administration, resources, supervision, validation, writing – original draft, and writing – review & editing; and A.G., conceptualization, funding acquisition, project administration, resources, supervision, validation, writing – original draft, and writing – review & editing.

DECLARATION OF INTERESTS

The gRNAs used for the PIR strategy for intron 30 and intron 36 as a therapeutic strategy to correct splicing defects is filed under the application no. EP24161788.5. R.W.J.C. and A.G. would like to declare that they are inventors on several patents describing the use of antisense oligonucleotides to target some of the variants in *ABCA4* intron 30 and intron 36 (WO2013036105A1, WO2018109011A1, WO2020015959A1, WO2020115106A1, WO2021023863A1), which have been out-licensed to Astherna. I.V.-D. is currently partially employed by Astherna, but all work in this manuscript was performed before this appointment. R.W.J.C. is founder and Chief Scientific Officer of Astherna.

SUPPLEMENTAL INFORMATION

Supplemental information can be found online at https://doi.org/10.1016/j.omtn.2024.102345.

REFERENCES

1. Allikmets, R., Singh, N., Sun, H., Shroyer, N.F., Hutchinson, A., Chidambaram, A., Gerrard, B., Baird, L., Stauffer, D., Peiffer, A., et al. (1997). A photoreceptor cell-specific ATP-binding transporter gene (ABCR) is mutated in recessive Stargardt macular dystrophy. Nat. Genet. *15*, 236–246.

2. Cremers, F.P., van de Pol, D.J., van Driel, M., den Hollander, A.I., van Haren, F.J., Knoers, N.V., Tijmes, N., Bergen, A.A., Rohrschneider, K., Blankenagel, A., et al. (1998). Autosomal recessive retinitis pigmentosa and cone-rod dystrophy caused by splice site mutations in the Stargardt's disease gene ABCR. Hum. Mol. Genet. *7*, 355–362.

3. Lambertus, S., Lindner, M., Bax, N.M., Mauschitz, M.M., Nadal, J., Schmid, M., Schmitz-Valckenberg, S., den Hollander, A.I., Weber, B.H.F., Holz, F.G., et al. (2016). Progression of Late-Onset Stargardt Disease. Invest. Ophthalmol. Vis. Sci. *57*, 5186–5191.

4. Al-Khuzaei, S., Broadgate, S., Foster, C.R., Shah, M., Yu, J., Downes, S.M., and Halford, S. (2021). An Overview of the Genetics of ABCA4 Retinopathies, an Evolving Story. Genes *12*, 1241.

5. Huang, D., Heath Jeffery, R.C., Aung-Htut, M.T., McLenachan, S., Fletcher, S., Wilton, S.D., and Chen, F.K. (2022). Stargardt disease and progress in therapeutic strategies. Ophthalmic Genet. *43*, 1–26.

6. Molday, R.S., Garces, F.A., Scortecci, J.F., and Molday, L.L. (2022). Structure and function of ABCA4 and its role in the visual cycle and Stargardt macular degeneration. Prog. Retin. Eye Res. *89*, 101036.

7. Khan, M., Arno, G., Fakin, A., Parfitt, D.A., Dhooge, P.P.A., Albert, S., Bax, N.M., Duijkers, L., Niblock, M., Hau, K.L., et al. (2020). Detailed Phenotyping and Therapeutic Strategies for Intronic ABCA4 Variants in Stargardt Disease. Mol. Ther. Nucleic Acids *21*, 412–427.

8. Cremers, F.P.M., Lee, W., Collin, R.W.J., and Allikmets, R. (2020). Clinical spectrum, genetic complexity and therapeutic approaches for retinal disease caused by ABCA4 mutations. Prog. Retin. Eye Res. *79*, 100861.

9. Corradi, Z., Khan, M., Hitti-Malin, R., Mishra, K., Whelan, L., Cornelis, S.S., ABCA4-Study Group, Hoyng, C.B., Kämpjärvi, K., Klaver, C.C.W., et al. (2023). Targeted sequencing and *in vitro* splice assays shed light on ABCA4-associated retinopathies missing heritability. HGG Adv. *4*, 100237.

10. Sangermano, R., Garanto, A., Khan, M., Runhart, E.H., Bauwens, M., Bax, N.M., van den Born, L.I., Khan, M.I., Cornelis, S.S., Verheij, J., et al. (2019). Deep-intronic ABCA4 variants explain missing heritability in Stargardt disease and allow correction of splice defects by antisense oligonucleotides. Genet. Med. *21*, 1751–1760.

11. Bauwens, M., Garanto, A., Sangermano, R., Naessens, S., Weisschuh, N., De Zaeytijd, J., Khan, M., Sadler, F., Balikova, I., Van Cauwenbergh, C., et al. (2019). ABCA4-associated disease as a model for missing heritability in autosomal recessive disorders: novel noncoding splice, cis-regulatory, structural, and recurrent hypomorphic variants. Genet. Med. *21*, 1761–1771.

12. Braun, T.A., Mullins, R.F., Wagner, A.H., Andorf, J.L., Johnston, R.M., Bakall, B.B., Deluca, A.P., Fishman, G.A., Lam, B.L., Weleber, R.G., et al. (2013). Non-exomic and synonymous variants in ABCA4 are an important cause of Stargardt disease. Hum. Mol. Genet. *22*, 5136–5145.

13. Bax, N.M., Sangermano, R., Roosing, S., Thiadens, A.A.H.J., Hoefsloot, L.H., van den Born, L.I., Phan, M., Klevering, B.J., Westeneng-van Haaften, C., Braun, T.A., et al. (2015). Heterozygous deep-intronic variants and deletions in ABCA4 in persons with retinal dystrophies and one exonic ABCA4 variant. Hum. Mutat. *36*, 43–47.

14. Albert, S., Garanto, A., Sangermano, R., Khan, M., Bax, N.M., Hoyng, C.B., Zernant, J., Lee, W., Allikmets, R., Collin, R.W.J., and Cremers, F.P.M. (2018). Identification and Rescue of Splice Defects Caused by Two Neighboring Deep-Intronic ABCA4 Mutations Underlying Stargardt Disease. Am. J. Hum. Genet. *102*, 517–527.

15. Khan, M., Cornelis, S.S., Khan, M.I., Elmelik, D., Manders, E., Bakker, S., Derks, R., Neveling, K., van de Vorst, M., Gilissen, C., et al. (2019). Cost-effective molecular inversion probe-based ABCA4 sequencing reveals deep-intronic variants in Stargardt disease. Hum. Mutat. *40*, 1749–1759.

16. Jespersgaard, C., Fang, M., Bertelsen, M., Dang, X., Jensen, H., Chen, Y., Bech, N., Dai, L., Rosenberg, T., Zhang, J., et al. (2019). Molecular genetic analysis using targeted NGS analysis of 677 individuals with retinal dystrophy. Sci. Rep. *9*, 1219.

17. Khan, M., Cornelis, S.S., Pozo-Valero, M.D., Whelan, L., Runhart, E.H., Mishra, K., Bults, F., AlSwaiti, Y., AlTalbishi, A., De Baere, E., et al. (2020). Resolving the dark matter of ABCA4 for 1054 Stargardt disease probands through integrated genomics and transcriptomics. Genet. Med. *22*, 1235–1246.

18. Bauwens, M., De Zaeytijd, J., Weisschuh, N., Kohl, S., Meire, F., Dahan, K., Depasse, F., De Jaegere, S., De Ravel, T., De Rademaeker, M., et al. (2015). An augmented ABCA4 screen targeting noncoding regions reveals a deep intronic founder variant in Belgian Stargardt patients. Hum. Mutat. *36*, 39–42.

19. Zernant, J., Xie, Y.A., Ayuso, C., Riveiro-Alvarez, R., Lopez-Martinez, M.A., Simonelli, F., Testa, F., Gorin, M.B., Strom, S.P., Bertelsen, M., et al. (2014). Analysis of the ABCA4 genomic locus in Stargardt disease. Hum. Mol. Genet. *23*, 6797–6806.

20. Tomkiewicz, T.Z., Suarez-Herrera, N., Cremers, F.P.M., Collin, R.W.J., and Garanto, A. (2021). Antisense Oligonucleotide-Based Rescue of Aberrant Splicing Defects Caused by 15 Pathogenic Variants in ABCA4. Int. J. Mol. Sci. *22*, 4621.

21. Garanto, A., Duijkers, L., Tomkiewicz, T.Z., and Collin, R.W.J. (2019). Antisense Oligonucleotide Screening to Optimize the Rescue of the Splicing Defect Caused by the Recurrent Deep-Intronic ABCA4 Variant c.4539+2001G>A in Stargardt Disease. Genes *10*, 452.

22. Shen, X., and Corey, D.R. (2018). Chemistry, mechanism and clinical status of antisense oligonucleotides and duplex RNAs. Nucleic Acids Res. *46*, 1584–1600.

23. Wu, W., Tang, L., D'Amore, P.A., and Lei, H. (2017). Application of CRISPR-Cas9 in eye disease. Exp. Eye Res. *161*, 116–123.

24. Liu, X., Ding, Q., Wang, W., Pan, Y., Tan, C., Qiu, Y., Chen, Y., Li, H., Li, Y., Ye, N., et al. (2022). Targeted Deletion of the First Intron of the Wx(b) Allele via CRISPR/Cas9 Significantly Increases Grain Amylose Content in Rice. Rice *15*, 1.

25. Sanz, D.J., Hollywood, J.A., Scallan, M.F., and Harrison, P.T. (2017). Cas9/gRNA targeted excision of cystic fibrosis-causing deep-intronic splicing mutations restores normal splicing of CFTR mRNA. PLoS One *12*, e0184009.

26. Panagiotopoulos, A.L., Karguth, N., Pavlou, M., Böhm, S., Gasparoni, G., Walter, J., Graf, A., Blum, H., Biel, M., Riedmayr, L.M., and Becirovic, E. (2020). Antisense Oligonucleotide- and CRISPR-Cas9-Mediated Rescue of mRNA Splicing for a Deep Intronic CLRN1 Mutation. Mol. Ther. Nucleic Acids *21*, 1050–1061.

27. Fenner, B.J., Tan, T.E., Barathi, A.V., Tun, S.B.B., Yeo, S.W., Tsai, A.S.H., Lee, S.Y., Cheung, C.M.G., Chan, C.M., Mehta, J.S., and Teo, K.Y.C. (2021). Gene-Based Therapeutics for Inherited Retinal Diseases. Front. Genet. *12*, 794805.

28. Hung, S.S., Li, F., Wang, J.H., King, A.E., Bui, B.V., Liu, G.S., and Hewitt, A.W. (2018). Methods for In Vivo CRISPR/Cas Editing of the Adult Murine Retina. Methods Mol. Biol. *1715*, 113–133.

29. Gasiunas, G., Young, J.K., Karvelis, T., Kazlauskas, D., Urbaitis, T., Jasnauskaite, M., Grusyte, M.M., Paulraj, S., Wang, P.H., Hou, Z., et al. (2020). A catalogue of biochemically diverse CRISPR-Cas9 orthologs. Nat. Commun. *11*, 5512.

30. Zuris, J.A., Thompson, D.B., Shu, Y., Guilinger, J.P., Bessen, J.L., Hu, J.H., Maeder, M.L., Joung, J.K., Chen, Z.Y., and Liu, D.R. (2015). Cationic lipid-mediated delivery of proteins enables efficient protein-based genome editing *in vitro* and *in vivo*. Nat. Biotechnol. *33*, 73–80.

31. Wei, T., Cheng, Q., Min, Y.L., Olson, E.N., and Siegwart, D.J. (2020). Systemic nanoparticle delivery of CRISPR-Cas9 ribonucleoproteins for effective tissue specific genome editing. Nat. Commun. *11*, 3232.

32. Wang, M., Zuris, J.A., Meng, F., Rees, H., Sun, S., Deng, P., Han, Y., Gao, X., Pouli, D., Wu, Q., et al. (2016). Efficient delivery of genome-editing proteins using bioreducible lipid nanoparticles. Proc. Natl. Acad. Sci. USA *113*, 2868–2873.

33. Gao, X., Tao, Y., Lamas, V., Huang, M., Yeh, W.H., Pan, B., Hu, Y.J., Hu, J.H., Thompson, D.B., Shu, Y., et al. (2018). Treatment of autosomal dominant hearing loss by *in vivo* delivery of genome editing agents. Nature *553*, 217–221.

34. Chen, G., Abdeen, A.A., Wang, Y., Shahi, P.K., Robertson, S., Xie, R., Suzuki, M., Pattnaik, B.R., Saha, K., and Gong, S. (2019). A biodegradable nanocapsule delivers a Cas9 ribonucleoprotein complex for *in vivo* genome editing. Nat. Nanotechnol. *14*, 974–980.

35. Deng, S., Li, X., Liu, S., Chen, J., Li, M., Chew, S.Y., Leong, K.W., and Cheng, D. (2020). Codelivery of CRISPR-Cas9 and chlorin e6 for spatially controlled tumor-specific gene editing with synergistic drug effects. Sci. Adv. *6*, eabb4005.

36. Krishnamurthy, S., Wohlford-Lenane, C., Kandimalla, S., Sartre, G., Meyerholz, D.K., Théberge, V., Hallée, S., Duperré, A.M., Del'Guidice, T., Lepetit-Stoffaes, J.P., et al. (2019). Engineered amphiphilic peptides enable delivery of proteins and CRISPR-associated nucleases to airway epithelia. Nat. Commun. *10*, 4906.

37. Kuhn, J., Lin, Y., Krhac Levacic, A., Al Danaf, N., Peng, L., Höhn, M., Lamb, D.C., Wagner, E., and Lächelt, U. (2020). Delivery of Cas9/sgRNA Ribonucleoprotein Complexes via Hydroxystearyl Oligoamino Amides. Bioconjug. Chem. *31*, 729–742.

38. Foss, D.V., Muldoon, J.J., Nguyen, D.N., Carr, D., Sahu, S.U., Hunsinger, J.M., Wyman, S.K., Krishnappa, N., Mendonsa, R., Schanzer, E.V., et al. (2023). Peptide-mediated delivery of CRISPR enzymes for the efficient editing of primary human lymphocytes. Nat. Biomed. Eng. *7*, 647–660.

39. Zhang, Z., Baxter, A.E., Ren, D., Qin, K., Chen, Z., Collins, S.M., Huang, H., Komar, C.A., Bailer, P.F., Parker, J.B., et al. (2024). Efficient engineering of human and mouse primary cells using peptide-assisted genome editing. Nat. Biotechnol. *42*, 305–315.

40. Alsaiari, S.K., Patil, S., Alyami, M., Alamoudi, K.O., Aleisa, F.A., Merzaban, J.S., Li, M., and Khashab, N.M. (2018). Endosomal Escape and Delivery of CRISPR/Cas9 Genome Editing Machinery Enabled by Nanoscale Zeolitic Imidazolate Framework. J. Am. Chem. Soc. *140*, 143–146.

41. Gong, J., Wang, H.X., Lao, Y.H., Hu, H., Vatan, N., Guo, J., Ho, T.C., Huang, D., Li, M., Shao, D., and Leong, K.W. (2020). A Versatile Nonviral Delivery System for Multiplex Gene-Editing in the Liver. Adv. Mater. *32*, e2003537.

42. Tang, J., Liu, J., Zheng, Q., Li, W., Sheng, J., Mao, L., and Wang, M. (2021). In-Situ Encapsulation of Protein into Nanoscale Hydrogen-Bonded Organic Frameworks for Intracellular Biocatalysis. Angew. Chem. Int. Ed. Engl. *60*, 22315–22321.

43. Sun, W., Ji, W., Hall, J.M., Hu, Q., Wang, C., Beisel, C.L., and Gu, Z. (2015). Self-assembled DNA nanoclews for the efficient delivery of CRISPR-Cas9 for genome editing. Angew. Chem. Int. Ed. Engl. *54*, 12029–12033.

44. Ramakrishna, S., Kwaku Dad, A.B., Beloor, J., Gopalappa, R., Lee, S.K., and Kim, H. (2014). Gene disruption by cell-penetrating peptide-mediated delivery of Cas9 protein and guide RNA. Genome Res. *24*, 1020–1027.

45. Suresh, B., Ramakrishna, S., and Kim, H. (2017). Cell-Penetrating Peptide-Mediated Delivery of Cas9 Protein and Guide RNA for Genome Editing. Methods Mol. Biol. *1507*, 81–94.

46. Lin, Y., Luo, X., Burghardt, T., Dorrer, S., Höhn, M., Wagner, E., and Lächelt, U. (2023). Chemical Evolution of Amphiphilic Xenopeptides for Potentiated Cas9 Ribonucleoprotein Delivery. J. Am. Chem. Soc. *145*, 15171–15179.

47. Gustafsson, O., Radler, J., Roudi, S., Lehto, T., Hallbrink, M., Lehto, T., Gupta, D., Andaloussi, S.E., and Nordin, J.Z. (2021). Efficient Peptide-Mediated In Vitro Delivery of Cas9 RNP. Pharmaceutics *13*, 878.

48. Lostale-Seijo, I., Louzao, I., Juanes, M., and Montenegro, J. (2017). Peptide/Cas9 nanostructures for ribonucleoprotein cell membrane transport and gene edition. Chem. Sci. *8*, 7923–7931.

49. Kichler, A., Leborgne, C., März, J., Danos, O., and Bechinger, B. (2003). Histidine-rich amphipathic peptide antibiotics promote efficient delivery of DNA into mammalian cells. Proc. Natl. Acad. Sci. USA *100*, 1564–1568.

50. Oktem, M., Mastrobattista, E., and de Jong, O.G. (2023). Amphipathic Cell-Penetrating Peptide-Aided Delivery of Cas9 RNP for In Vitro Gene Editing and Correction. Pharmaceutics *15*, 2500.

51. Kurrikoff, K., Veiman, K.L., Künnapuu, K., Peets, E.M., Lehto, T., Pärnaste, L., Arukuusk, P., and Langel, Ü. (2017). Effective *in vivo* gene delivery with reduced toxicity, achieved by charge and fatty acid -modified cell penetrating peptide. Sci. Rep. *7*, 17056.

52. Li, Y., Wang, Y., Wei, Q., Zheng, X., Tang, L., Kong, D., and Gong, M. (2015). Variant fatty acid-like molecules Conjugation, novel approaches for extending the stability of therapeutic peptides. Sci. Rep. *5*, 18039.

53. Thach, T.T., Bae, D.H., Kim, N.H., Kang, E.S., Lee, B.S., Han, K., Kwak, M., Choi, H., Nam, J., Bae, T., et al. (2019). Lipopeptide-Based Nanosome-Mediated Delivery of Hyperaccurate CRISPR/Cas9 Ribonucleoprotein for Gene Editing. Small *15*, e1903172.

54. de Jong, O.G., Murphy, D.E., Mäger, I., Willms, E., Garcia-Guerra, A., Gitz-Francois, J.J., Lefferts, J., Gupta, D., Steenbeek, S.C., van Rheenen, J., et al. (2020). A CRISPR-Cas9-based reporter system for single-cell detection of extracellular vesicle-mediated functional transfer of RNA. Nat. Commun. *11*, 1113.

55. Glaser, A., McColl, B., and Vadolas, J. (2016). GFP to BFP Conversion: A Versatile Assay for the Quantification of CRISPR/Cas9-mediated Genome Editing. Mol. Ther. Nucleic Acids *5*, e334.

56. Tomkiewicz, T.Z., Nieuwenhuis, S.E., Cremers, F.P.M., Garanto, A., and Collin, R.W.J. (2022). Correction of the Splicing Defect Caused by a Recurrent Variant in ABCA4 (c.769-784C>T) That Underlies Stargardt Disease. Cells *11*, 3947.

57. Karjosukarso, D.W., Bukkems, F., Duijkers, L., Leijsten, N., Hoyng, C.B., and Collin, R.W.J. (2023). Generation of iPSC lines from three Stargardt patients carrying bi-allelic ABCA4 variants. Stem Cell Res. *71*, 103164.

58. Karjosukarso, D.W., Bukkems, F., Duijkers, L., Leijsten, N., and Collin, R.W.J. (2023). Generation of three isogenic control lines from patient-derived iPSCs carrying bi-allelic ABCA4 variants underlying Stargardt disease. Stem Cell Res. *71*, 103169.

59. Vazquez-Dominguez, I., Li, C.H.Z., Fadaie, Z., Haer-Wigman, L., Cremers, F.P.M., Garanto, A., Hoyng, C.B., and Roosing, S. (2022). Identification of a Complex Allele in IMPG2 as a Cause of Adult-Onset Vitelliform Macular Dystrophy. Invest. Ophthalmol. Vis. Sci. *63*, 27.

60. Vazquez-Dominguez, I., Duijkers, L., Fadaie, Z., Alaerds, E.C.W., Post, M.A., van Oosten, E.M., O'Gorman, L., Kwint, M., Koolen, L., Hoogendoorn, A.D.M., et al. (2022). The Predicted Splicing Variant c.11+5G>A in RPE65 Leads to a Reduction in mRNA Expression in a Cell-Specific Manner. Cells *11*, 3640.

61. Cong, L., Ran, F.A., Cox, D., Lin, S., Barretto, R., Habib, N., Hsu, P.D., Wu, X., Jiang, W., Marraffini, L.A., and Zhang, F. (2013). Multiplex genome engineering using CRISPR/Cas systems. Science *339*, 819–823.

62. Xie, F., Ye, L., Chang, J.C., Beyer, A.I., Wang, J., Muench, M.O., and Kan, Y.W. (2014). Seamless gene correction of beta-thalassemia mutations in patient-specific iPSCs using CRISPR/Cas9 and piggyBac. Genome Res. *24*, 1526–1533.

63. Montagna, C., Petris, G., Casini, A., Maule, G., Franceschini, G.M., Zanella, I., Conti, L., Arnoldi, F., Burrone, O.R., Zentilin, L., et al. (2018). VSV-G-Enveloped Vesicles for Traceless Delivery of CRISPR-Cas9. Mol. Ther. Nucleic Acids *12*, 453–462.

64. Ye, L., Wang, J., Beyer, A.I., Teque, F., Cradick, T.J., Qi, Z., Chang, J.C., Bao, G., Muench, M.O., Yu, J., et al. (2014). Seamless modification of wild-type induced pluripotent stem cells to the natural CCR5Delta32 mutation confers resistance to HIV infection. Proc. Natl. Acad. Sci. USA *111*, 9591–9596.

65. Schumann, K., Lin, S., Boyer, E., Simeonov, D.R., Subramaniam, M., Gate, R.E., Haliburton, G.E., Ye, C.J., Bluestone, J.A., Doudna, J.A., and Marson, A. (2015). Generation of knock-in primary human T cells using Cas9 ribonucleoproteins. Proc. Natl. Acad. Sci. USA *112*, 10437–10442.

66. Jinek, M., East, A., Cheng, A., Lin, S., Ma, E., and Doudna, J. (2013). RNA-programmed genome editing in human cells. Elife *2*, e00471.

67. Amabile, A., Migliara, A., Capasso, P., Biffi, M., Cittaro, D., Naldini, L., and Lombardo, A. (2016). Inheritable Silencing of Endogenous Genes by Hit-and-Run Targeted Epigenetic Editing. Cell *167*, 219–232.e14.

68. da Cruz, L., Dorn, J.D., Humayun, M.S., Dagnelie, G., Handa, J., Barale, P.O., Sahel, J.A., Stanga, P.E., Hafezi, F., Safran, A.B., et al. (2016). Five-Year Safety and Performance Results from the Argus II Retinal Prosthesis System Clinical Trial. Ophthalmology *123*, 2248–2254.

69. Diao, Y., Li, B., Meng, Z., Jung, I., Lee, A.Y., Dixon, J., Maliskova, L., Guan, K.L., Shen, Y., and Ren, B. (2016). A new class of temporarily phenotypic enhancers identified by CRISPR/Cas9-mediated genetic screening. Genome Res. *26*, 397–405.

70. Romanel, A., Garritano, S., Stringa, B., Blattner, M., Dalfovo, D., Chakravarty, D., Soong, D., Cotter, K.A., Petris, G., Dhingra, P., et al. (2017). Inherited determinants of early recurrent somatic mutations in prostate cancer. Nat. Commun. *8*, 48.

71. Chen, Z., Liu, F., Chen, Y., Liu, J., Wang, X., Chen, A.T., Deng, G., Zhang, H., Liu, J., Hong, Z., and Zhou, J. (2017). Targeted Delivery of CRISPR/Cas9-Mediated Cancer Gene Therapy via Liposome-Templated Hydrogel Nanoparticles. Adv. Funct. Mater. *27*, 1703036.

72. Lee, Y.W., Mout, R., Luther, D.C., Liu, Y., Castellanos-García, L., Burnside, A.S., Ray, M., Tonga, G.Y., Hardie, J., Nagaraj, H., et al. (2019). In Vivo Editing of

Macrophages through Systemic Delivery of CRISPR-Cas9-Ribonucleoprotein-Nanoparticle Nanoassemblies. Adv. Ther. *2*, 1900041.

73. Mout, R., Ray, M., Yesilbag Tonga, G., Lee, Y.W., Tay, T., Sasaki, K., and Rotello, V.M. (2017). Direct Cytosolic Delivery of CRISPR/Cas9-Ribonucleoprotein for Efficient Gene Editing. ACS Nano *11*, 2452–2458.

74. Wang, Q., Yu, J., Kadungure, T., Beyene, J., Zhang, H., and Lu, Q. (2018). ARMMs as a versatile platform for intracellular delivery of macromolecules. Nat. Commun. *9*, 960.

75. Vazquez-Dominguez, I., Garanto, A., and Collin, R.W.J. (2019). Molecular Therapies for Inherited Retinal Diseases-Current Standing, Opportunities and Challenges. Genes *10*, 654.

76. Seko, Y., Iwanami, M., Miyamoto-Matsui, K., Takita, S., Aoi, N., Umezawa, A., and Kato, S. (2018). The manner of decay of genetically defective EYS gene transcripts in photoreceptor-directed fibroblasts derived from retinitis pigmentosa patients depends on the type of mutation. Stem Cell Res. Ther. *9*, 279.

77. Kaltak, M., de Bruijn, P., Piccolo, D., Lee, S.E., Dulla, K., Hoogenboezem, T., Beumer, W., Webster, A.R., Collin, R.W.J., Cheetham, M.E., et al. (2023). Antisense oligonucleotide therapy corrects splicing in the common Stargardt disease type 1-causing variant ABCA4 c.5461-10T>C. Mol. Ther. Nucleic Acids *31*, 674–688.

78. Pollock, N.L., McDevitt, C.A., Collins, R., Niesten, P.H.M., Prince, S., Kerr, I.D., Ford, R.C., and Callaghan, R. (2014). Improving the stability and function of purified ABCB1 and ABCA4: the influence of membrane lipids. Biochim. Biophys. Acta *1838*, 134–147.

79. Suarez-Herrera, N., Li, C.H.Z., Leijsten, N., Karjosukarso, D.W., Corradi, Z., Bukkems, F., Duijkers, L., Cremers, F.P.M., Hoyng, C.B., Garanto, A., and Collin, R.W.J. (2024). Preclinical Development of Antisense Oligonucleotides to Rescue Aberrant Splicing Caused by an Ultrarare ABCA4 Variant in a Child with Early-Onset Stargardt Disease. Cells *13*, 601.

80. Siles, L., Ruiz-Nogales, S., Navinés-Ferrer, A., Méndez-Vendrell, P., and Pomares, E. (2023). Efficient correction of ABCA4 variants by CRISPR-Cas9 in hiPSCs derived from Stargardt disease patients. Mol. Ther. Nucleic Acids *32*, 64–79.

81. De Angeli, P., Reuter, P., Hauser, S., Schöls, L., Stingl, K., Wissinger, B., and Kohl, S. (2022). Effective splicing restoration of a deep-intronic ABCA4 variant in cone photoreceptor precursor cells by CRISPR/SpCas9 approaches. Mol. Ther. Nucleic Acids *29*, 511–524.

82. Dyka, F.M., Molday, L.L., Chiodo, V.A., Molday, R.S., and Hauswirth, W.W. (2019). Dual ABCA4-AAV Vector Treatment Reduces Pathogenic Retinal A2E Accumulation in a Mouse Model of Autosomal Recessive Stargardt Disease. Hum. Gene Ther. *30*, 1361–1370.

83. McClements, M.E., Barnard, A.R., Charbel Issa, P., and MacLaren, R.E. (2020). Assessment of AAV Dual Vector Safety in theAbca4(-/-) Mouse Model of Stargardt Disease. Transl. Vis. Sci. Technol. *9*, 20.

84. Tornabene, P., Trapani, I., Centrulo, M., Marrocco, E., Minopoli, R., Lupo, M., Iodice, C., Gesualdo, C., Simonelli, F., Surace, E.M., and Auricchio, A. (2021). Inclusion of a degron reduces levelsof undesired inteins after AAV-mediated proteintrans-splicing in the retina. Mol. Ther. Methods Clin. Dev. *23*, 448–459.

85. Wang, D., Tai, P.W.L., and Gao, G. (2019). Adeno-associated virus vector as a platform for gene therapy delivery. Nat. Rev. Drug Discov. *18*, 358–378.

86. Garanto, A., Chung, D.C., Duijkers, L., Corral-Serrano, J.C., Messchaert, M., Xiao, R., Bennett, J., Vandenberghe, L.H., and Collin, R.W.J. (2016). *In vitro* and *in vivo* rescue of aberrant splicing in CEP290-associated LCA by antisense oligonucleotide delivery. Hum. Mol. Genet. *25*, 2552–2563.

87. Gillmore, J.D., Gane, E., Taubel, J., Kao, J., Fontana, M., Maitland, M.L., Seitzer, J., O'Connell, D., Walsh, K.R., Wood, K., et al. (2021). CRISPR-Cas9 In Vivo Gene Editing for Transthyretin Amyloidosis. N. Engl. J. Med. *385*, 493–502.

88. Wang, T., Wei, J.J., Sabatini, D.M., and Lander, E.S. (2014). Genetic screens in human cells using the CRISPR-Cas9 system. Science *343*, 80–84.

89. Linde, L., Boelz, S., Neu-Yilik, G., Kulozik, A.E., and Kerem, B. (2007). The efficiency of nonsense-mediated mRNA decay is an inherent character and varies among different cells. Eur. J. Hum. Genet. *15*, 1156–1162.

90. De Angeli, P., Flores-Tufiño, A., Stingl, K., Kühlewein, L., Roschi, E., Wissinger, B., and Kohl, S. (2024). Splicing defects and CRISPR-Cas9 correction in isogenic homozygous photoreceptor precursors harboring clustered deep-intronic ABCA4 variants. Mol. Ther. Nucleic Acids *35*, 102113.

91. Rosazza, C., Meglic, S.H., Zumbusch, A., Rols, M.P., and Miklavcic, D. (2016). Gene Electrotransfer: A Mechanistic Perspective. Curr. Gene Ther. *16*, 98–129.

92. Matsuda, T., and Cepko, C.L. (2004). Electroporation and RNA interference in the rodent retina *in vivo* and *in vitro*. Proc. Natl. Acad. Sci. USA *101*, 16–22.

93. Chalberg, T.W., Vankov, A., Molnar, F.E., Butterwick, A.F., Huie, P., Calos, M.P., and Palanker, D.V. (2006). Gene transfer to rabbit retina with electron avalanche transfection. Invest. Ophthalmol. Vis. Sci. *47*, 4083–4090.

94. Johnson, C.J., Berglin, L., Chrenek, M.A., Redmond, T.M., Boatright, J.H., and Nickerson, J.M. (2008). Technical brief: subretinal injection and electroporation into adult mouse eyes. Mol. Vis. *14*, 2211–2226.

95. Touchard, E., Benard, R., Bigot, K., Laffitte, J.D., Buggage, R., Bordet, T., and Behar-Cohen, F. (2018). Non-viral ocular gene therapy, pEYS606, for the treatment of non-infectious uveitis: Preclinical evaluation of the medicinal product. J. Control. Release *285*, 244–251.

96. Gyorgy, B., Muller, A., Sullivan, J., Schwarzer, W., Wang, M., Park-Windhol, C., Hasler, P.W., Moreau, P.H., Azoulay, T., Janeschitz-Kriegl, L., et al. (2023). Adenine-base Editing Corrects the Most Common ABCA4 Mutation Causing Stargardt Disease. Invest. Ophthalmol. Vis. Sci. *64*.

97. Maeder, M.L., Stefanidakis, M., Wilson, C.J., Baral, R., Barrera, L.A., Bounoutas, G.S., Bumcrot, D., Chao, H., Ciulla, D.M., DaSilva, J.A., et al. (2019). Development of a gene-editing approach to restore vision loss in Leber congenital amaurosis type 10. Nat. Med. *25*, 229–233.

98. (2020). First CRISPR therapy dosed. Nat. Biotechnol. *38*, 382.

99. Pierce, E.A., Aleman, T.S., Jayasundera, K.T., Ashimatey, B.S., Kim, K., Rashid, A., Jaskolka, M.C., Myers, R.L., Lam, B.L., Bailey, S.T., et al. (2024). Gene Editing for CEP290-Associated Retinal Degeneration. N. Engl. J. Med. *390*, 1972–1984.

100. Wong, C. (2023). UK first to approve CRISPR treatment for diseases: what you need to know. Nature *623*, 676–677.

101. Azevedo, C., Macedo, M.H., and Sarmento, B. (2018). Strategies for the enhanced intracellular delivery of nanomaterials. Drug Discov. Today *23*, 944–959.

102. Lehto, T., Vasconcelos, L., Margus, H., Figueroa, R., Pooga, M., Hällbrink, M., and Langel, Ü. (2017). Saturated Fatty Acid Analogues of Cell-Penetrating Peptide PepFect14: Role of Fatty Acid Modification in Complexation and Delivery of Splice-Correcting Oligonucleotides. Bioconjug. Chem. *28*, 782–792.

103. Schneider, C., Rasband, W., and Eliceiri, K. (2012). NIH Image to ImageJ: 25 years of image analysis. Nat Methods *9*, 671–675.

104. Walther, J., Porenta, D., Wilbie, D., Seinen, C., Benne, N., Yang, Q., de Jong, O.G., Lei, Z., and Mastrobattista, E. (2023). Comparative Analysis of Lipid Nanoparticle-Mediated Delivery of CRISPR-Cas9 RNP Versus mRNA/sgRNA for Gene Editing *in vitro* and *in vivo*. Eur. J. Pharm. Biopharm. *196*, 114207.

105. Sentmanat, M.F., Peters, S.T., Florian, C.P., Connelly, J.P., and Pruett-Miller, S.M. (2018). A Survey of Validation Strategies for CRISPR-Cas9 Editing. Sci. Rep. *8*, 888.

106. Gonzalez-Cordero, A., Kruczek, K., Naeem, A., Fernando, M., Kloc, M., Ribeiro, J., Goh, D., Duran, Y., Blackford, S.J.I., Abelleira-Hervas, L., et al. (2017). Recapitulation of Human Retinal Development from Human Pluripotent Stem Cells Generates Transplantable Populations of Cone Photoreceptors. Stem Cell Rep. *9*, 820–837.

107. Capowski, E.E., Samimi, K., Mayerl, S.J., Phillips, M.J., Pinilla, I., Howden, S.E., Saha, J., Jansen, A.D., Edwards, K.L., Jager, L.D., et al. (2019). Reproducibility and staging of 3D human retinal organoids across multiple pluripotent stem cell lines. Development *146*, dev171686.

108. Livak, K.J., and Schmittgen, T.D. (2001). Analysis of relative gene expression data using real-time quantitative PCR and the 2(-Delta Delta C(T)) Method. Methods *25*, 402–408.

OMTN, Volume *35*

Supplemental information

Lipopeptide-mediated Cas9 RNP delivery: A promising broad therapeutic strategy for safely removing deep-intronic variants in *ABCA4*

Irene Vázquez-Domínguez, Mert Öktem, Florian A. Winkelaar, Thai Hoang Nguyen, Anita D.M. Hoogendoorn, Eleonora Roschi, Galuh D.N. Astuti, Raoul Timmermans, Nuria Suárez-Herrera, Ilaria Bruno, Albert Ruiz-Llombart, Joseph Brealey, Olivier G. de Jong, Rob W.J. Collin, Enrico Mastrobattista, and Alejandro Garanto

Supplemental Material

Methods

In vitro cytotoxicity assay

HEK293T stoplight,[1] HeLa, and eGFP HEPA 1-6 cells were cultured in a Greiner Bio-One 96 well-plate at a density of 3 x 10^4 cells per well. After a 24-hour incubation period, cells were transfected using a 100 µl solution of RPNC (RNP/C18:1-LAH5). These were prepared presence of 20 nM RNP and 1 µM to 20 µM lipopeptide and following at a ratio of RNP:lipopeptide ranging from 1:50 to 1:1000. As a reference, positive control wells were treated with a 100 µL of medium enriched with 5% Triton X-100. This experimental procedure was conducted in triplicate for each condition.

Twenty-four hours after transfection, cell viability was assessed by treating the cells with a 20 µl solution of CellTiter 96® Aqueous one solution cell proliferation assay (MTS) reagent (Promega Corporation, USA), following the manufacturer's recommended protocol. Fluorescence intensity was quantified with an absorbance set of 490 nm using an IMark™ Microplate Reader (Bio-Rad, Hercules, California, USA). Data was normalized by excluding reference positive control signals from negative control and treated samples.

Transfection of pX458 constructs in HEK293T

A total of 400,000 cells of HEK293T were transfected with the pX458 vector (Addgene, #48138) in which the designed gRNAs were introduced after the U6 promoter. The transfection was done using FuGENE®-HD (Promega, Madison, WI, USA) following a 3:1 FuGENE®-HD Transfection Reagent: DNA ratio. The FuGENE®-HD/DNA mixture was prepared in Opti-MEM reduced serum medium (Gibco, Waltham, MA, USA). Samples were harvested 48 h after transfection to proceed with DNA analysis.

Protein isolation and Western blot analysis in PPCs

After 12 days of RPNC delivery, PPCs were harvested (between day 38-39 of differentiation) for protein analysis. PPCs cells were washed in 1x PBS, mechanically detached in 200 µl of RIPA (50 mM Tris pH 7.5, 1 mM EDTA, 150 mM NaCl, 0.5% Na-Deoxycholate, 1% NP-40, 0.75% SDS) supplemented with cOmplete^{TM} Mini Protease Inhibitor Cocktail (Roche,Manheim, Germany), and sonicated for 15 s/sample. Protein concentration was determined by using the Pierce^{TM} BCA Protein Assay Kit (Thermo Fisher Scientific, Waltham, MA, USA) according to manufacturer's indications. A total of 55 µg of protein per condition were loaded on 4-15% Mini-PROTEAN® TGX Stain-Free pre-cast gels (Bio-Rad) and electrophoresed for 45 min at 200 V in Tris-Glycine-SDS buffer (Bio-Rad, Hercules, CA, USA). Transfer to Trans-Blot Turbo Mini 0.2 µm nitrocellulose membranes (Bio-Rad) was conducted using the Trans-Blot Turbo Transfer System (Bio-Rad). After blocking in 5% blotto non-fatty milk diluted in 1x PBS during 1 h at RT, membranes were incubated with the primary antibody overnight at 4°C. The next day, blots were washed 3 times for 5 minutes in 1x PBS supplemented with 0.2% of Tween. Then, blots were incubated with the corresponding IRDye secondary antibody for 1 h at RT in the dark. Prior reveling the membrane, membranes were washed with 1x PBS – 0.2% Tween as previously indicated. Membranes were developed in the Odyssey Imaging System (Li-COR Biosciences, Lincoln, NE, USA). Intensity of the ABCA4 protein bands was semi-quantified using Fiji software 1.53t, with normalization to β-tubulin bands and further normalization to the non-treated (NT) condition. Employed antibodies and their dilution conditions can be found on **Table S9**.

Analysis of Whole Genome sequencing (WGS) and long-read sequencing

To confirm both on-target and off-target effects, we analyzed two DNA samples each from control and patient PPCs. These samples included the non-treated (NT) condition and the

treated with RPNC including gRNAs 30-1 and 30-2. None of the samples underwent treatment with CHX.

Long-read (LR) sequencing for on-target validation:

To assess the on-target effects after RPNC delivery with the two gRNAs, targeted PacBio long-read sequencing of the amplicon was performed. We amplified intron 30 from PPC DNA as previously described in the main manuscript. The resulting amplicons of 2936 bp in the non-treated samples and 2936 bp together with an amplicon 323 bp in the treated samples were assessed on an agarose gel and measured in Qubit. A total amount of 350 - 500 ng was used for long-read sequencing. The PCR product was then purified using AMPure PB beads (Pacific Biosciences, Menlo Park, CA, USA) with a bead ratio of 1.5x. The library was prepared according to the protocol *"Procedure and Checklist-Preparing SMRTbell Libraries using PacBio Barcoded Adapters for Multiplex SMRT Sequencing"* (Pacific Biosciences, part number 100-538-700-02) as published previously.[2] Briefly, after measuring the purified sample with Qubit, an equimolar amount of amplicons were used to perform a one-step end and adapter ligation using barcoded hairpin adapters), followed by equimolar pooling. The pool was purified with AMPure PB Beads, followed by DNA damage repair, exonuclease digestion, and two additional rounds of AMPure PB Beads purification.

Polymerase bound SMRT-bell complexes and SMRT sequencing were then performed. To generate the complexes, the Sample Setup option in SMRTLink (Pacific Biosciences) was used. Briefly, sequencing primers were conditioned and annealed to the SMRTbell library, followed by dilution and binding of the sequencing polymerase. The polymerase-bound complex was purified using AMPure PB beads and the concentration measured using Qubit. An internal control sample was diluted and added to the polymerase-bound complex. Sequencing was then performed using the Run Design option in SMRTLink. Libraries were loaded at a plate concentration of 4.5 pM. All runs were sequenced using a movie time of 20 h

per SMRTcell and included pre-extension. Sequencing was performed on a Sequel I system (Pacific Biosciences) using ICS version 8.0. Finally, targeted long-read sequencing was performed on a Sequel II machine (Pacific Biosciences). Subreads were demultiplexed using lima V.2.5.0. The subreads were combined into a consensus sequence using CCS V.6.3.0, these reads were filtered for RQ 0.99 to obtain HiFi reads. The HiFi reads were then mapped along the GRCh38 reference genome using pbmm2 V1.8.0 with the "--preset ISOSEQ" parameter.

Whole genome-sequencing (WGS) for off-target analysis:

A minimum of 1 µg of DNA of the aforementioned samples were sent to BGI Genomics Poland. The library type used was for amplicon ≤ 800 bp using the sequencing platform DNBseq™. Prediction of the possible off-targets for gRNA 30.1 and gRNA 30.2 was done using the web tool: Off-Spotter (https://cm.jefferson.edu/Off-Spotter/), indicating the none position is using as sit. From the output list all targets with 3 mismatches or less were analyzed as potential off-targets (**Table S4**). Then, the predicted SVs, SNVs, and off-target areas were manually inspected using Integrative Genomics Viewer 2.4 software [3], by aligning the reads with the Human Reference Genome (GRCh38.p14).

Repair profile analysis

To assess the mutational profile after genome editing of intron 30 of *ABCA4*, we first extracted correctly mapped reads from our PacBio data. We then filtered these reads by size, removing all those > 1000 nt. This resulted in 19136 and 10399 reads left in the data from the isogenic control and patient PPCs, respectively. Subsequently, we filtered out all those reads that were identified fewer than five times, leaving 9444 and 4554 reads explaining 34 and 17 different editing events in the isogenic control and patient edited PPC cells, respectively. In total, 44 different repair events were identified, of which seven events were shared between both lines.

The top 3 events explained ~82% of all reads and they were shared by both lines at similar ratios (**Table S5**).

Supplemental Figures

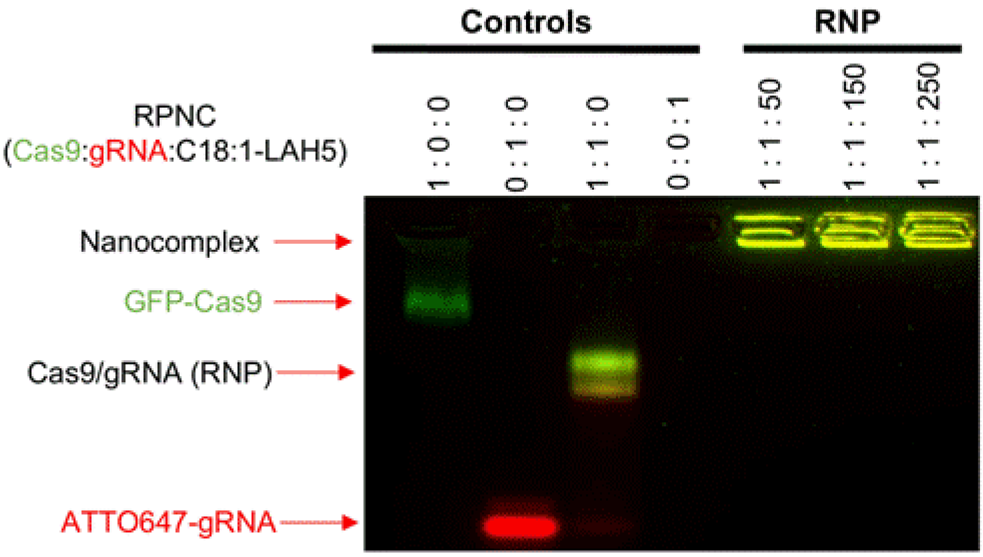

Figure S1. Electrophoretic mobility shift assay showing the effect of increasing C18:1-LAH5 lipopeptide concentration on RPNC formation. The efficient capacity of C18:1-LAH5 lipopeptide to form complexes with RNP was tested in escalating lipopeptide concentrations through an agarose-based electrophoretic mobility shift assay (EMSA), employing equivalent quantities of ATTO647-gRNA and GFP-Cas9.

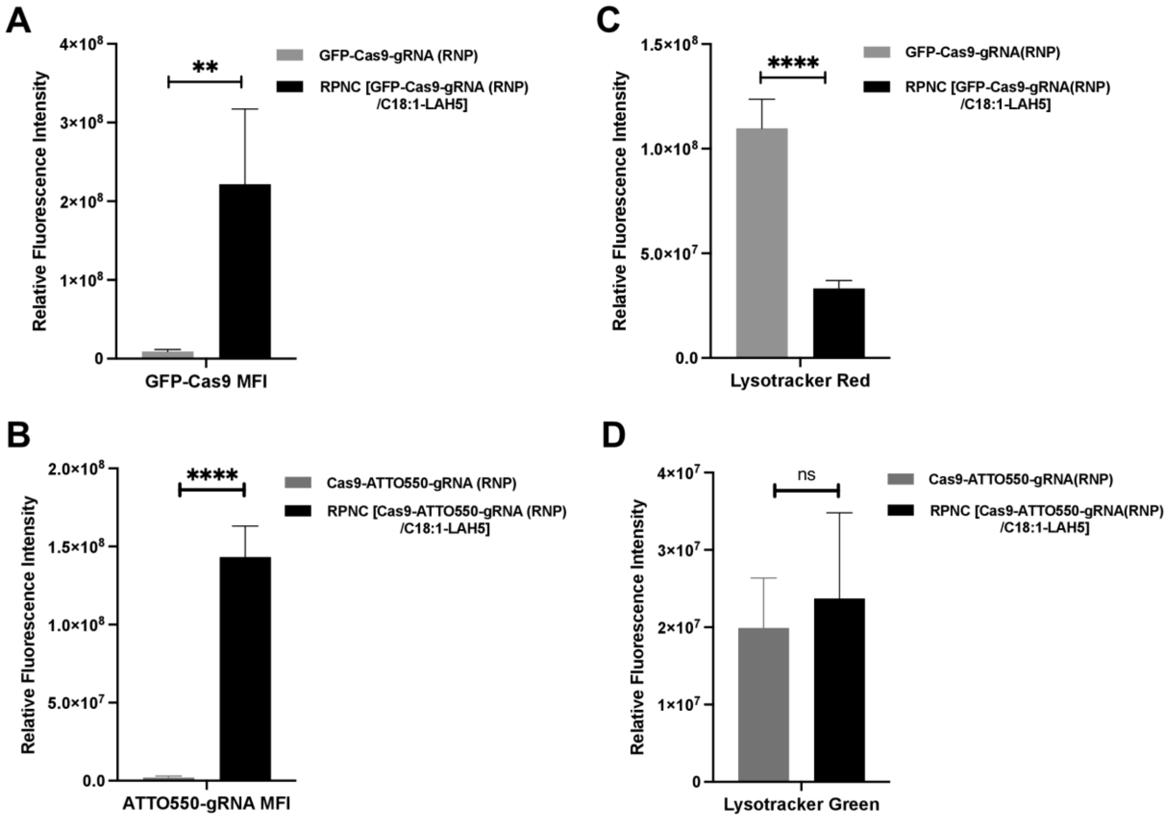

Figure S2. Relative cellular uptake of fluorescently labeled RNPs in the absence and presence of C18:1-LAH5. HeLa cells were treated with RPNCs, **A)** including GFP-Cas9/gRNA/C18:1-LAH5, **B)** non-labeled Cas9/ATTO 550-gRNA/C18:1-LAH5, with a molar ratio of 1:150. In addition, the cells were treated with **C)** Lysotracker red, and **D)** Lysotracker green. Control experiments were conducted using RNP preparations without the inclusion of C18:1-LAH5. The fluorescence of each material was imaged through confocal microscopy. The relative fluorescence intensity (RMFI) was quantified by measuring the total fluorescence intensity of the whole image using ImageJ, and RMFI was calculated based the following formula: *RMFI = fluorescence intensity of measured material value – background fluorescence intensity value.* The difference in RMFI of each material in non-lipopeptide treated and lipopeptide treated sample were compared using a two tailed unpaired t-test. (non-significant (ns), * $p < 0.05$, ** $p < 0.01$, *** $p < 0.001$, **** $p < 0.0001$).

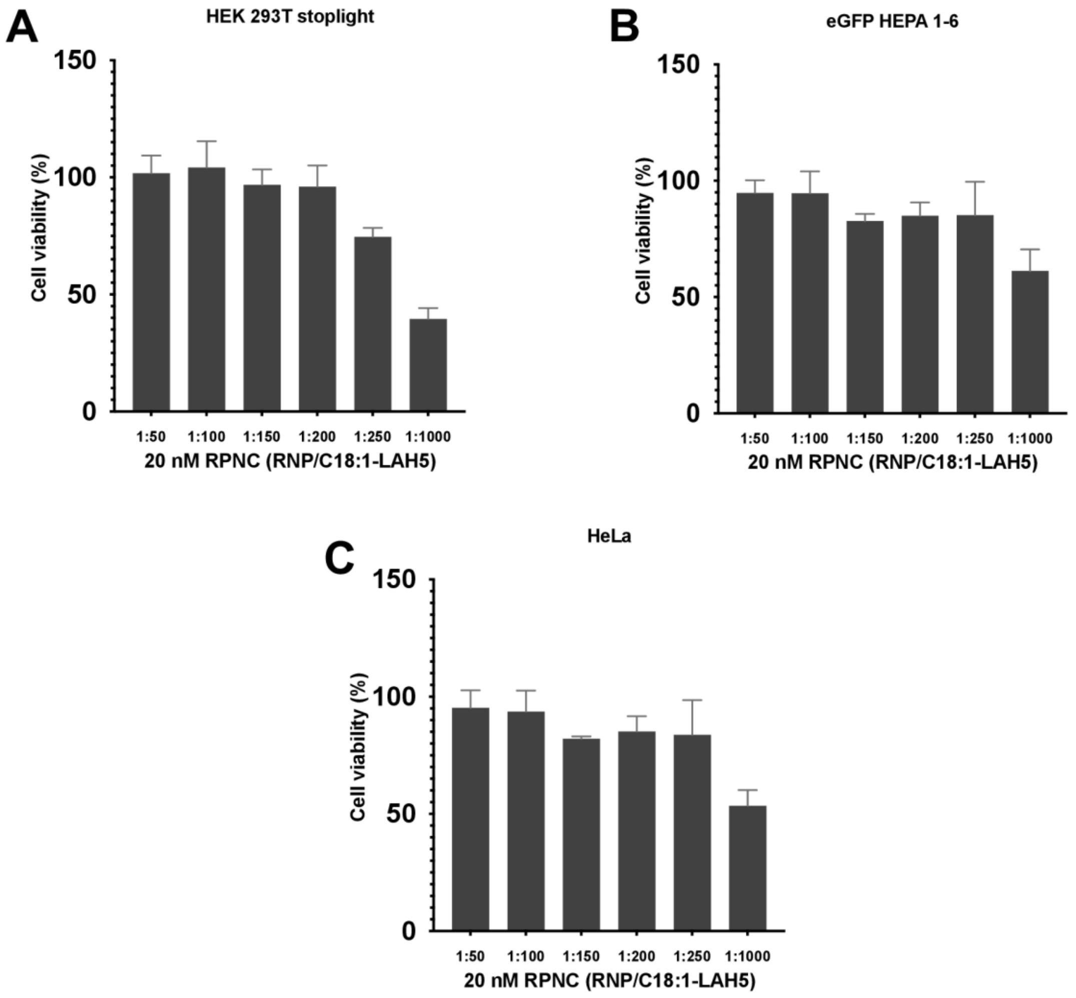

Figure S3. The cytotoxicity assessment of the RPNCs in HEK293T stoplight, eGFP HEPA 1-6, and HeLa cells. A) HEK293T, **B)** eGFP HEPA 1-6 and **C)** HeLa cells were treated with RNP/C18:1-LAH5 peptide (RPNC) at ratios ranging from 1:50 to 1:500, at a fixed concentration of 20 nM. After 24 h incubation, cell viability was tested using MTS assay and the fluorescence intensity was measure with an absorbance set of 490 nm. The normalization process involved excluding the reference positive control signal from both negative control and treated samples. Data shown as the mean ± SD (n=2).

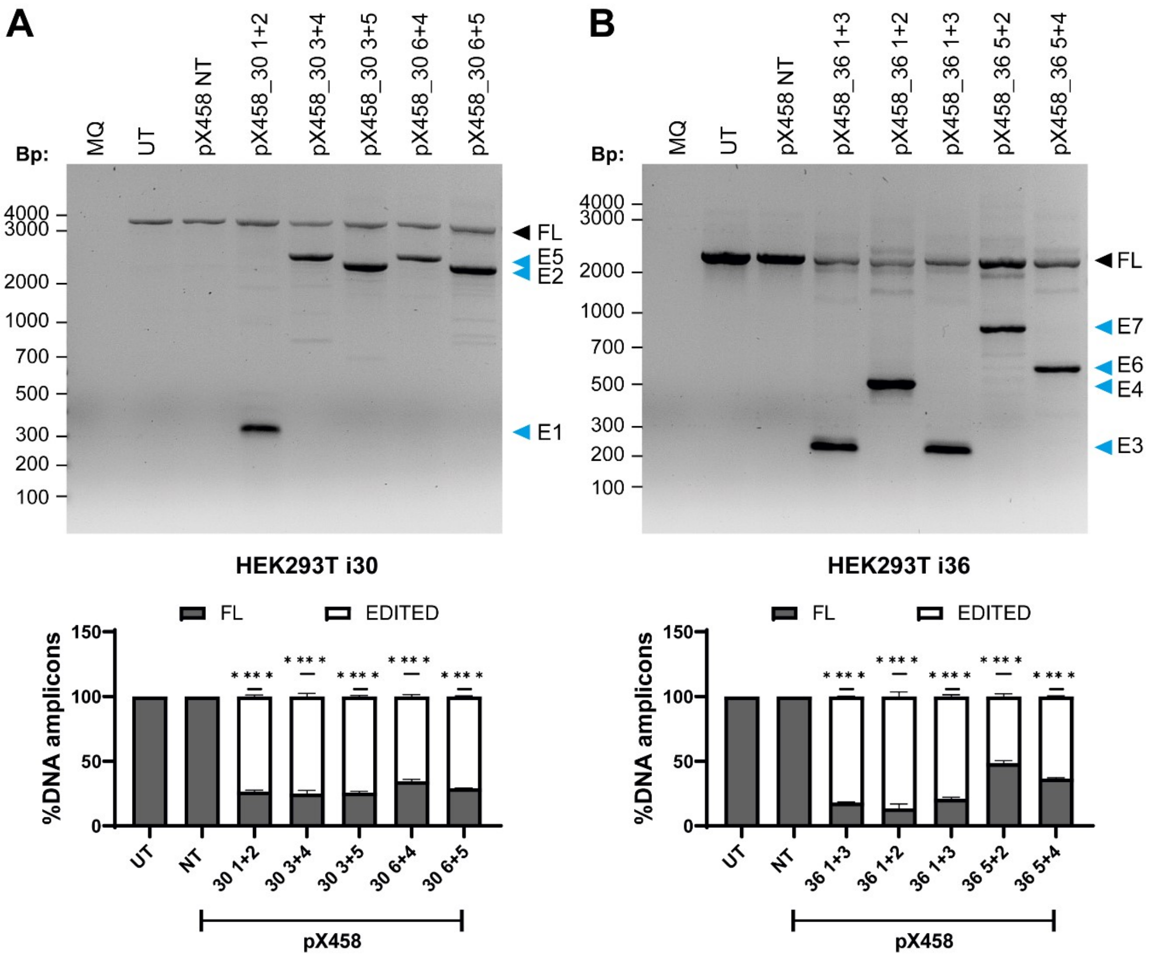

Figure S4. Screening of the efficiency of multiple pairs of gRNAs at genomic DNA level on intron 30 (A) or intron 36 (B) of *ABCA4* gene. On top, a representative electrophoresis gel of the analysis of the gRNAs targeting the intron 30 or intron 36 of *ABCA4.* Each column represents different combinations of transfected pX458 plasmids containing the indicated sgRNAs. These were compared to the empty pX548 vector (pX548 NT) or the untransfected condition (UT) allowing the identification of the expected edited bands, which are highlighted with a blue arrow. a graph chart representing the overall result of the conducted replicates (n=2) indicating the percentage of the full-length amplicon (FL), without editing fragment (EDITED) and the percentage of editing per each condition. Each bar is represented by the mean ± SD (n=2). Statistical significance with respect to the pX458 NT indicated as **** $p < 0.0001$ by one-way ANOVA test followed by Bonferroni correction. Of note, condition pX458_36 1+3 is presented double as same gRNA was named differently, and therefore same condition was performed twice.

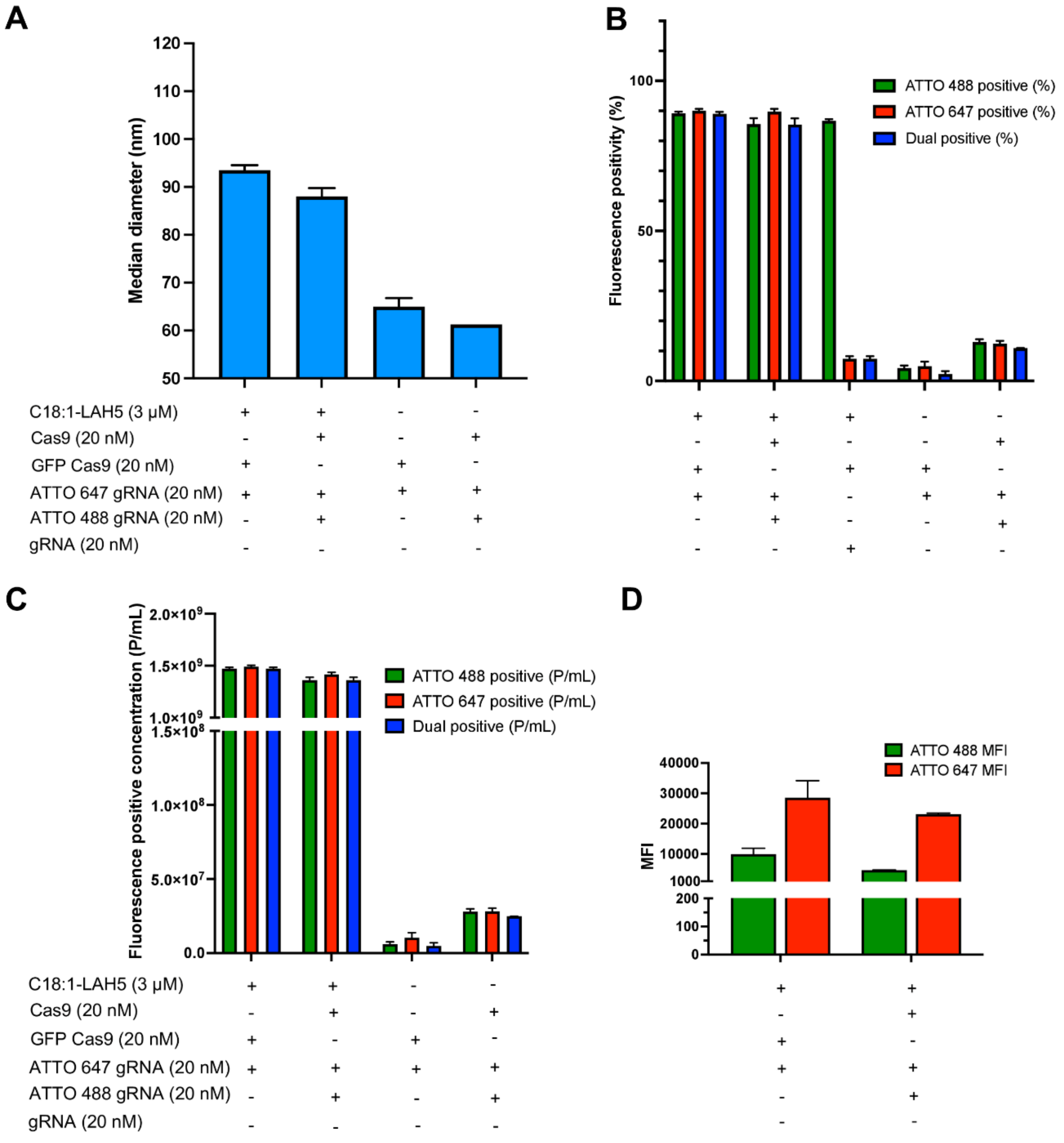

Figure S5. Peptide mediated nanocomplexation (RPNC formation) determined by Nano-Flow Cytometry. A) Median diameter of the RPNCs determined as determined by NanoFCM. The median diameters were consistently higher in the samples (GFP Cas9/ATTO 647 gRNA) (RNP) and samples (Cas9/ ATTO488-gRNA1/ATTO647-gRNA2) (RNP) when they were formulated with the C18:1-LAH5 peptide, as compared to samples that did not contain this peptide. Data shown: mean ± SD. Of note, the experiment was conducted in duplicate for samples containing C18:1-LAH5 in two independent trials, while the control samples without C18:1-LAH5 were conducted in duplicate in a single independent trial. **B)** Cas9/RNP encapsulation efficiency was defined as percentage of fluorescently labeled components positivity (GFP Cas9, ATTO 488 gRNA1, and ATTO647-gRNA2). The presence of the C18:1-LAH5 lipopeptide in the (GFP Cas9/ATTO647-gRNA) and (GFP Cas9/gRNA), as well as in the (Cas9/ ATTO488-gRNA1/ATTO647-gRNA2), resulted in higher levels of fluorescence positivity compared to samples that did not complex with the peptide. Data shown: mean ± SD (n=2). **C)** Cas9/RNP loading efficiency was defined as Fluorescence positive particle concentration (Particles/mL) of fluorescently labeled components (GFP Cas9, ATTO 488 gRNA1, and ATTO 647 gRNA2). The complexation with the C18:1-LAH5 peptide enhanced the concentration of fluorescence-positive particles (P/mL) in both the GFP Cas9/ATTO647-gRNA (RNP) and the Cas9/ATTO488-gRNA1/ATTO647-gRNA2 (RNP) RPNCs. **D)** MFI of RPNCs for gene editing and PIR. Data shown as the mean ± SD. The experiment was conducted in duplicate for samples containing C18:1-LAH5 in two independent trials, while the control samples without C18:1-LAH5 were conducted in duplicate in a single independent trial.

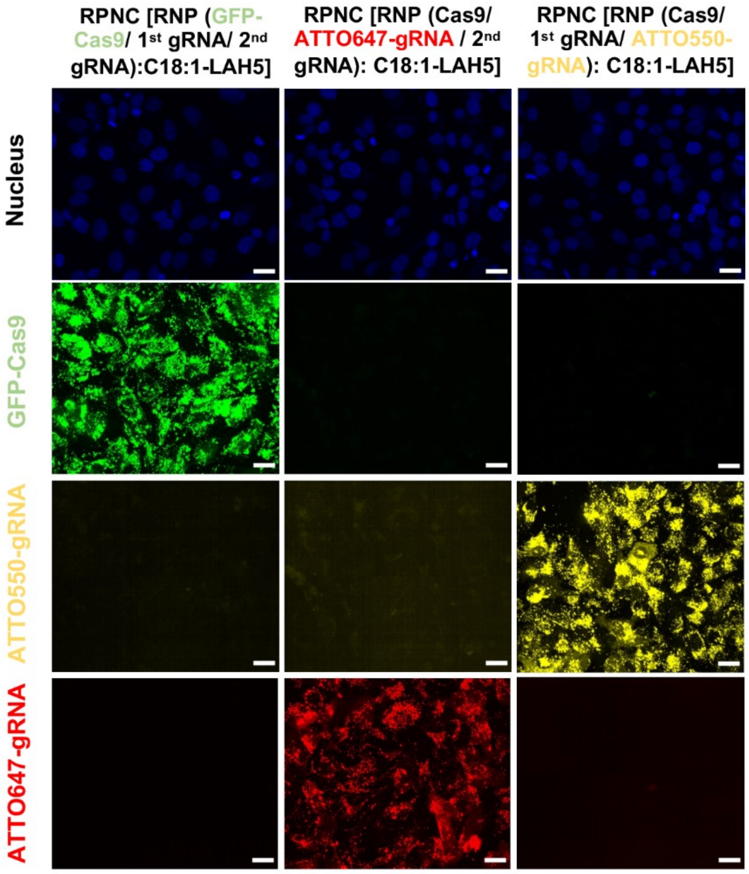

Figure S6. Verification of the simultaneous intracellular uptake of three different fluorescently labeled compounds. To validate the cellular uptake of three distinct fluorescently labeled molecules, HeLa cells were treated with the RPNC including only a single labeled component. Each individual component was combined in different combinations with GFP-Cas9/gRNA1/gRNA2, non-labeled Cas9/ATTO647-gRNA1/gRNA2, and non-labeled Cas9/gRNA1/ATTO550-gRNA2 to form ribonucleoprotein (RNP) complexes. Subsequently, these RNP complexes were combined with the C18:1-LAH5 lipopeptide to form RPNC, after which cells were treated with them. These control images were recorded 24 h after treatment. Scale bars represent 30 µm.

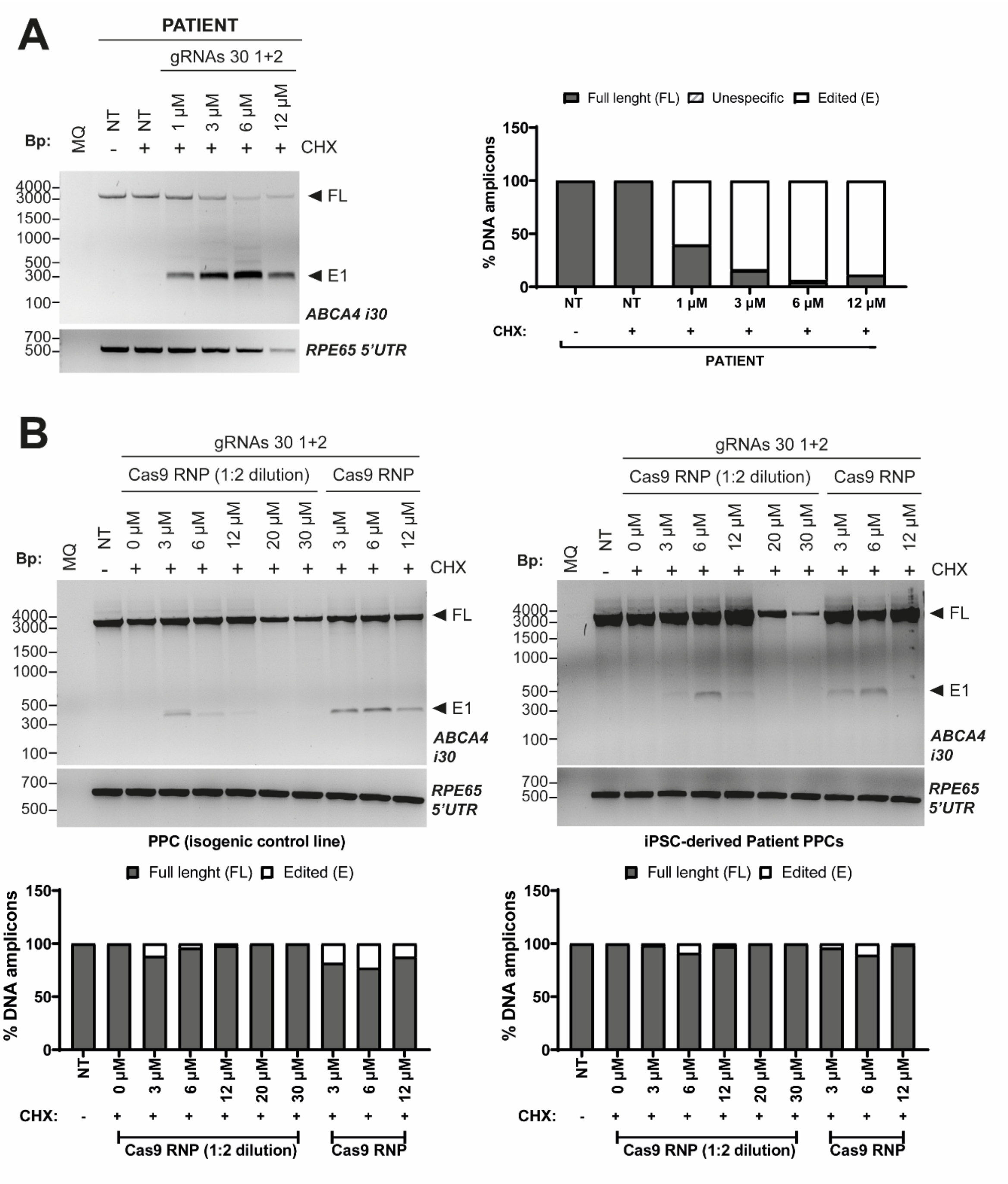

Figure S7. Dose-curve analysis of the RPNCs in fibroblast (A) and photoreceptor precursor cells (B) at genomic DNA level. A) On the left, representative electrophoresis gel of the amplification of intron 30 of *ABCA4* by PCR in both control and patient fibroblast cell lines. Edited band 1 (E1) indicates the expected edited band after treating with RPNC including gRNAs 30-1 and 30-2 after different lipopeptide dose treatment (from 1 – 12 µM). The 5´UTR region of *RPE65* was amplified as loading control. On the right, a graph chart representing the overall result (n=1) indicating the percentage of the full-length amplicon (FL), without editing and the percentage of editing (edited band, E) per each condition. Each bar is represented by median. In addition, some unspecific amplifications that do not correspond to the editing but neither associated with a specific PCR product were also represented. B) On top, representative electrophoresis gels of the amplification of the intron 30 in the isogenic control- (left) or patient-derived PPCs (right) line by PCR. In each, the full-length (FL) amplicon and the expected edited band (E1) are indicated on the right edge of the gel. Each column represents the treatment with the gRNA 30 1+2 together with different dilution of Cas9 RNP and different lipopeptide concentrations (0 – 30 µM). The 5´UTR region of *RPE65* was amplified as loading control. Below, a graph chart representing the overall result indicating the percentage of the full-length transcript (FL) and of the edited band (E) per each condition.

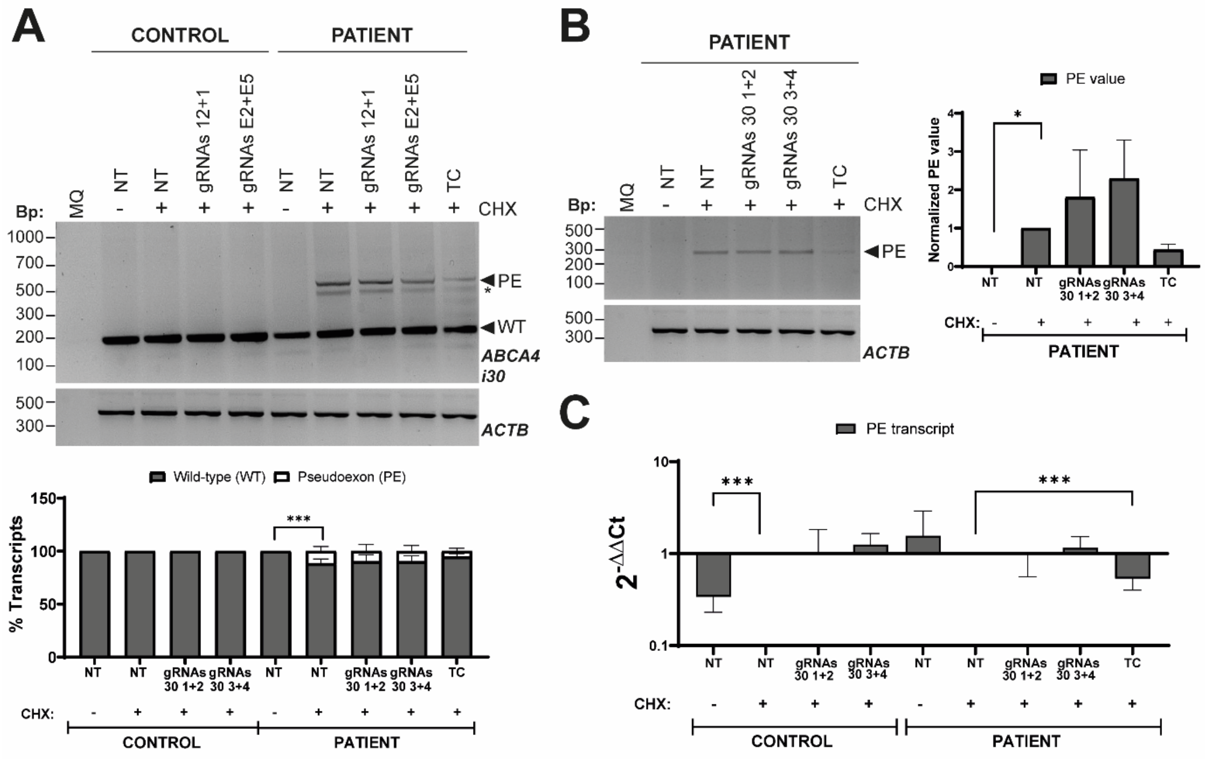

Figure S8. Analysis of the effect of the genome editing of intron 30 of *ABCA4* of fibroblasts at the RNA level of fibroblast. A) RT-PCR from exon 30 to exon 31 of *ABCA4* (n=4). On top, representative electrophoresis gel of the RT-PCR, below the graph chart showing the normalized value of the pseudoexon in comparison with the NT+ condition of control or patient fibroblast. Each bar represents the mean + SD. * means the presence of heteroduplex between the PE transcript and the WT transcript. B) RT-PCR which specific amplifies the pseudoexon (PE) transcript (n=4). On the left a representative electrophoresis gel of the analysis. On the right, a graph chart indicates the normalized value of pseudoexon transcript in comparison with NT+ of each condition of the two conducted replicated. Each bar represents the mean + SD. A and B) *ACTB* was amplified as a loading control. MQ indicates the negative control of the PCR; NT means non-treated cells. The trans-differentiation control (TC) consists of fibroblasts that were seeded and cultured together with the trans-differentiated fibroblasts. However, the TC fibroblasts were cultured without trans-differentiation medium, meaning that they did not undergo trans-differentiation. Therefore, they serve as a negative control for the trans-differentiation process. Statistical significance with respect to the untreated condition (NT +) is indicated as * $p < 0.05$ between NT- and NT+ by one-way ANOVA test followed by Bonferroni correction. This indicates that the CHX treatment worked. However, no statistical difference was observed with respect to the remaining remained conditions. C) qPCR of the pseudoexon (PE) in the control and patient line (n=4). Each condition was normalized against *GUSB* and then compared with the NT+ condition of each cell line. A to C) CHX indicates if cell were treated with (+) or without (-) cycloheximide 24 hours prior to harvesting. Statistical significance with respect to the untreated condition (NT +) is indicated as *** $p < 0.001$ between by T-test.

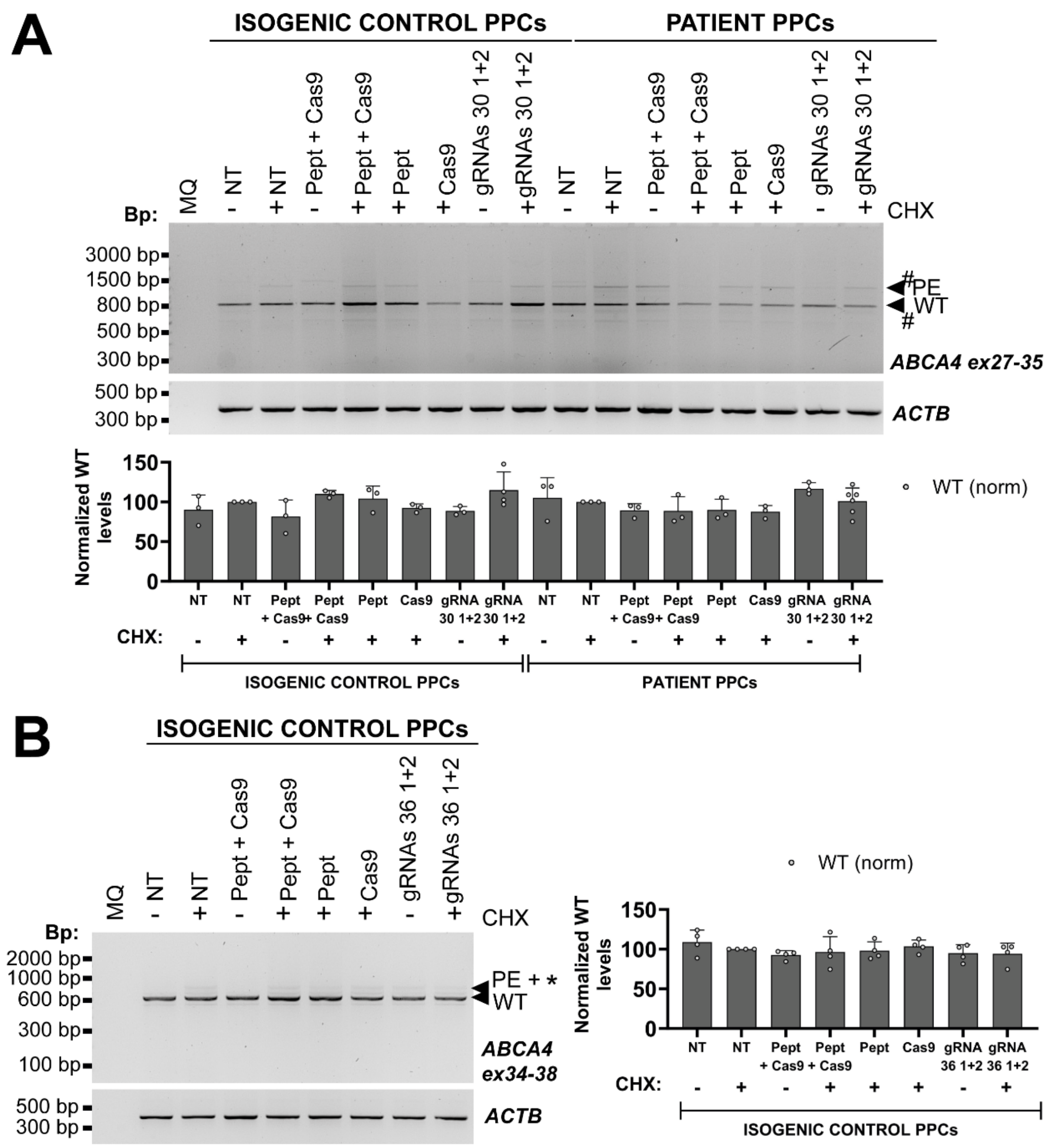

Figure S9. Analysis of the effect of the partial intron removal in the *ABCA4* gene after delivery of RPNC with gRNA30 1+2 or gRNA36 1+3 in photoreceptor precursor cells (PPCs) at RNA level. A) RT-PCR from exon 27 to exon 35 of *ABCA4* (n=3 with exception of samples for gRNAs 1+2 CHX+ whose n=4-6). Upper panel, a representative electrophoresis gel of the RT-PCR is shown. Two main transcripts are visible: wild-type transcript (WT) and the pseudo exon (PE) transcript. # denotes PCR artifacts that we could not assign to any specific transcript, but present in all samples independently of the treatment. Below, the graph chart displays the normalized expression of the amplified region against *ACTB,* which was used as loading control. NT CHX + samples (for each cell line) was set as reference at 100%. Each bar represents the mean + SD, where each light gray dot represents the normalized value obtained in each replicate. B) RT-PCR from exon 34 to exon 38 of *ABCA4* (n=4). On the left, a representative electrophoresis gel of the RT-PCR. Of note, the PE at intron 36 can naturally appear when using control PPC RNA as described previously (Khan et al., 2020[4]), this PE is more apparent after CHX treatment. Asterisk (*) indicates an heteroduplex between the WT and the PE transcripts. On the right, the graph chart showing the normalized expression of the WT transcript against *ACTB*, which was used as loading control. NT CHX + condition was set as reference at 100%. A and B) MQ indicates the negative control of the PCR; NT means non-treated cells. CHX indicates if cell were treated with (+) or without (-) cycloheximide 24 h prior to harvesting. MQ indicates the negative control of the PCR; NT means non-treated cells. CHX indicates if cell were treated with (+) or without (-) cycloheximide 24 h prior to harvesting. No statistical significance was found relative to the corresponding NT condition.

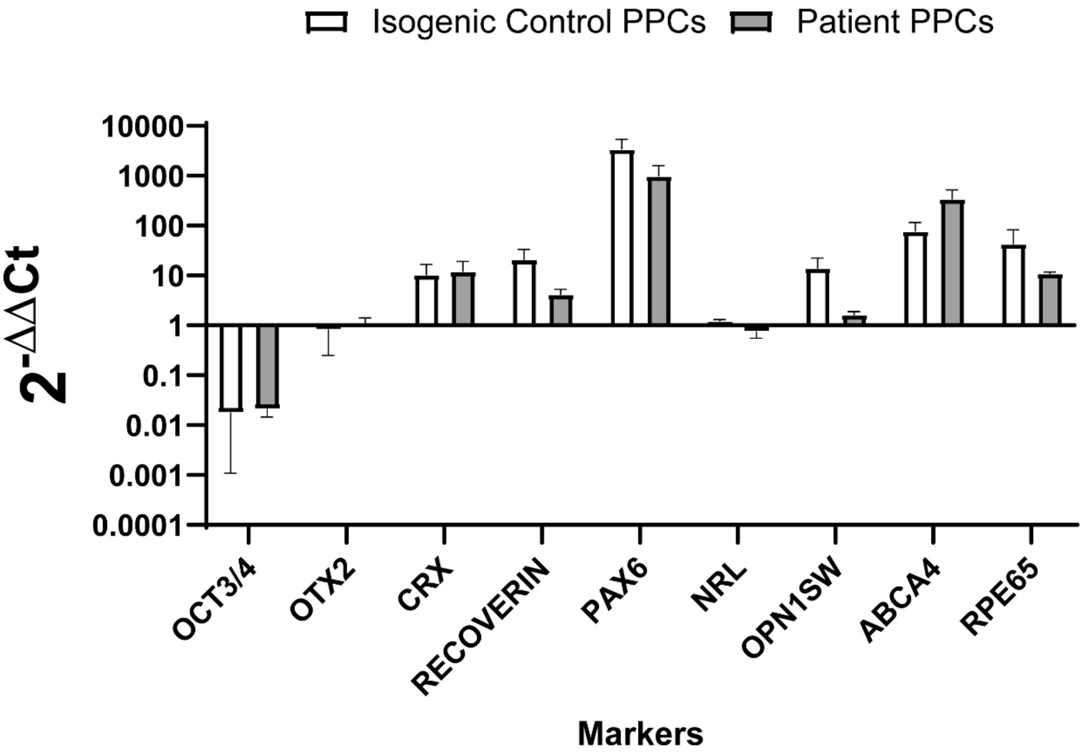

Figure S10. Gene expression profile analysis by qPCR of the photoreceptor progenitor cells (PPCs) differentiation markers in the isogenic control and patient-derived PPCs. Gene expression profile of isogenic control-derived PPC (with) and patient-derived PPCs (in grey) after 30 days of differentiation. The $2^{-\Delta\Delta CT}$ represents the relative expression against *GUSB* and then normalized to the relative value of the iPSC (Day 0 of the differentiation) (n=3). Differentiation into PPCs is observed by the reduced expression of the pluripotent marker *OCT3/4* and an increased expression of photoreceptor markers. Finally, isogenic control and patient-derived PPCs presented an increased expression of *ABCA4*.

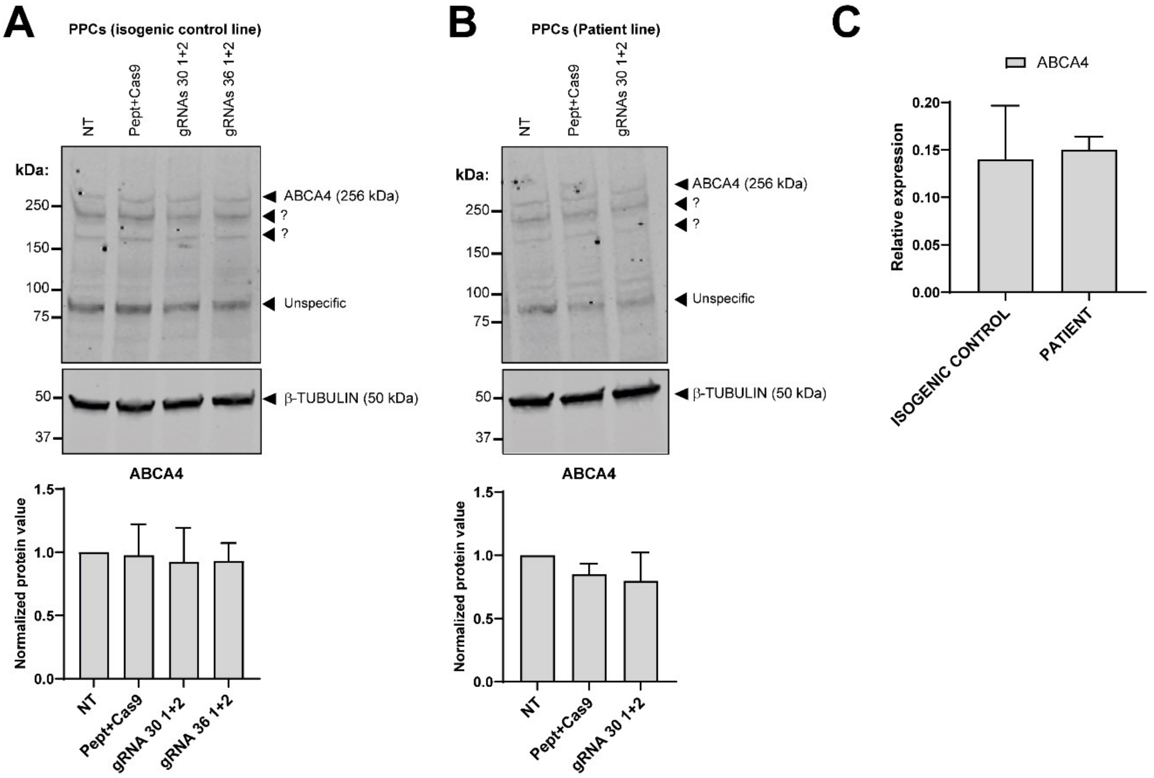

Figure S11. Western blot analysis on PPCs after 12 days of treatment with the RPNC with gRNAs 30-1 and 30-2. On day ~21 of differentiation, RPNCs were delivered to the isogenic control (A) and to patient PPCs (B). A and B) On top a representative western blot for ABCA4 and β-TUBULIN. The upper panel depicts the immunodetection of the ABCA4 protein (256 kDa). In this panel a known unspecific band at 80 kDa was also detected together with some unidentified (?) bands between 150 and 200 kDa. The detection of these bands is very variable, but they have been detected before in other PPC models or in fibroblasts (Suárez-Herrera et al, 2024[5]), so here they seem to be related to the heterogeneity of the PPC model. The lower panel shows β-TUBULIN (50 KDa), which was used as loading control and to normalize ABCA4 levels in each condition. Data (n=2) are represented below as the mean ± SD of the semi-quantification normalized against non-treated PPCs (NT). Pept+Cas9 refers to the cells treated with the RPNC complex but without gRNAs to evaluate possible effects on the RPNC at the protein levels. C) Graph bar representing the mean ± SD values of the relative expression of ABCA4 of the NT conditions in the isogenic control and the patient-derived PPCs. Data (n=2) are normalized only against β-TUBULIN values.

A

gRNA12							
Off-Target #	Chrom	Strand	Start	End	Given query	Actual genomic hit	# of mismatches
1	10	-	129808349	129808371	TGCTGCCCAGAAGTAGTTGA	gGCTGCCCAGAAGgAcTTGA-GGG	3
2	1	-	232832138	232832160	TGCTGCCCAGAAGTAGTTGA	aGCTGCCCAGAAGaAGTgGA-TGG	3
3	14	-	58940693	58940715	TGCTGCCCAGAAGTAGTTGA	TcCTGaCCAGAAGTAGTTGc-TGG	3
4	11	+	23891308	23891330	TGCTGCCCAGAAGTAGTTGA	TcCTGCtCAGAAGTAGTTcA-GGG	3
5	20	-	2658063	2658085	TGCTGCCCAGAAGTAGTTGA	TGtgGCCCAGAAGgAGTTGA-TGG	3
6	8	-	105805786	105805808	TGCTGCCCAGAAGTAGTTGA	TGaTGCCCAGAAGTgtTTGA-AGG	3
7	20	-	50677454	50677476	TGCTGCCCAGAAGTAGTTGA	TGCTctCCAGAAGTAGTTGg-GGG	3
8	4	+	75385660	75385682	TGCTGCCCAGAAGTAGTTGA	TGCTcCCCAGAAGcAGaTGA-TGG	3
9	17	+	71552069	71552091	TGCTGCCCAGAAGTAGTTGA	TGCTGgCCAGAAGcAGTgGA-TGG	3

B

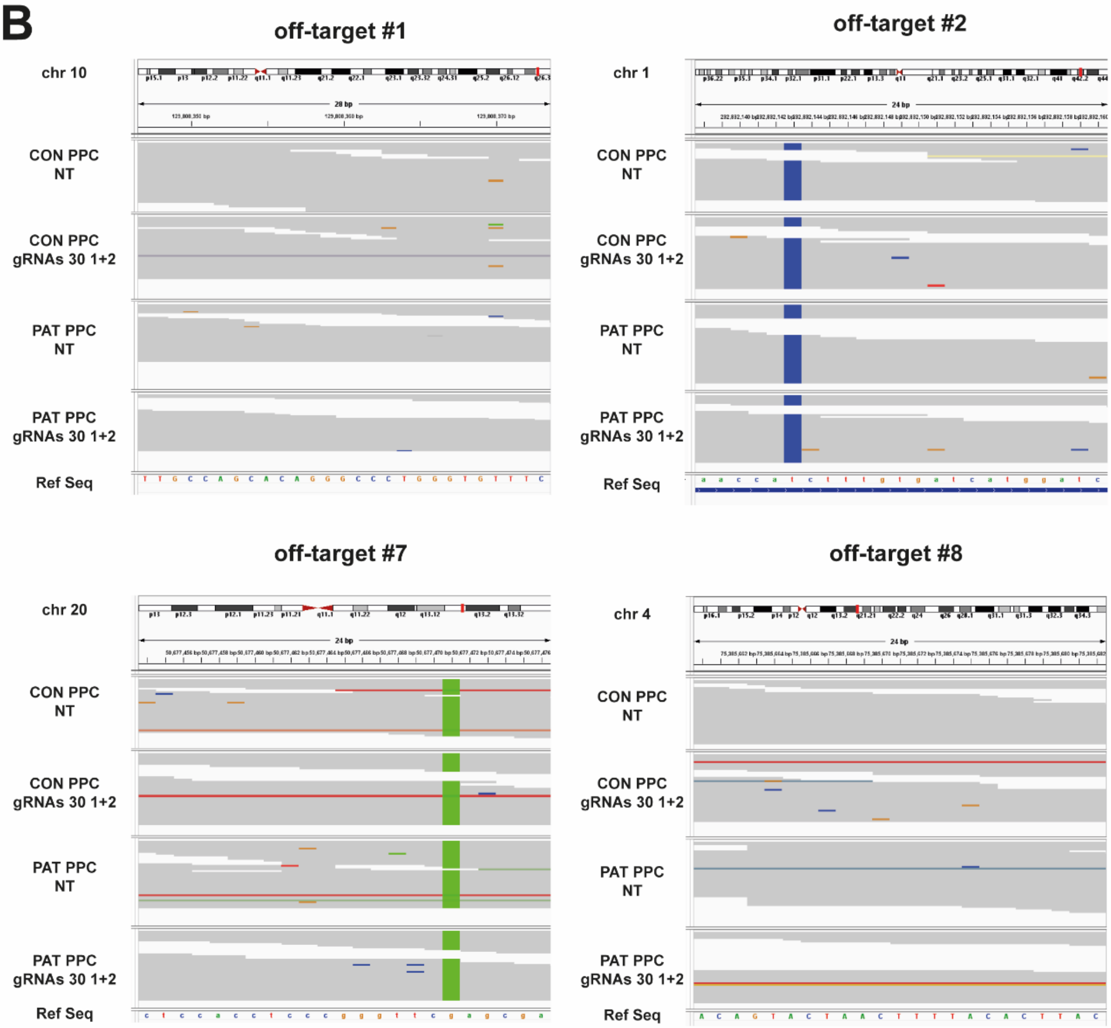

Figure S12. Summary of the analysis of the predicted off-targets for the gRNA 30-1 sequence in control and patient-derived PPCs. A) Table listing the position of the predicted off-targets for gRNA 30-1 indicating the chromosome (chrom), the strand (+ or -), the start and end position, the gRNA sequence (Given query) and the off-target sequence (Actual genomic hit). The mismatches are highlighted in red. The number of mismatches is indicated in the last column. **B)** Representative Integrative Genomics Viewer (IGV) browser screenshots of four off-target sites which showed more discrepancies between non-treated samples (NT) and edited samples. In none of the potential off-target, an effect caused by the gRNA transfection was detected. In all cases, grey color indicated no differences while squares with different color indicated a nucleotide change (C, blue; G, black; A, green and T, red). Some rows are a combination of gray and colored sections rows, indicating a partial overlap of the read with the reference sequence (Ref Seq). CON PPC means isogenic control-derived PPCs. PAT PPC means patient-derived PPCs. In the case off-target #2 and off-target #7 a SNP is emphasized in all samples in blue (T>C) and green (G>A), respectively.

A

gRNA 30-2 (Representative selection)							
**Off-Target #**	**Chrom**	**Strand**	**Start**	**End**	**Given query**	**Actual genomic hit**	**# of mismatches**
**1**	7	+	140319829	140319851	CTAAAACTACCAAAAAAATT	CTAAAAaTACCAAAAAAATT-AGG	1
**2**	16	+	17619500	17619522	CTAAAACTACCAAAAAAATT	CTAAAAaTACCAAAAAAATT-AGG	1
**3**	20	+	47511398	47511420	CTAAAACTACCAAAAAAATT	CTAAAAaTACCAAAAAAATT-AGG	1
**4**	3	+	49861718	49861740	CTAAAACTACCAAAAAAATT	CTAAAACTACaAAAAAAATT-AGG	1
**5**	9	+	36196110	36196132	CTAAAACTACCAAAAAAATT	CTAAAACTACaAAAAAAATT-AGG	1
**6**	17	+	36326901	36326923	CTAAAACTACCAAAAAAATT	tTAAAAaTACCAAAAAAATT-AGG	2
**7**	4	-	140694223	140694245	CTAAAACTACCAAAAAAATT	tTAAAACTACCAAAAAAATc-TGG	2
**8**	11	+	8925266	8925288	CTAAAACTACCAAAAAAATT	CaAAAAgTACCAAAAAAATT-AGG	2
**9**	1	+	65100512	65100534	CTAAAACTACCAAAAAAATT	CTAAAAaTACaAAAAAAATT-AGG	2
**10**	8	+	30563853	30563875	CTAAAACTACCAAAAAAATT	CTAAAAaTACaAAAAAAATT-AGG	2
**11**	10	+	14084798	14084820	CTAAAACTACCAAAAAAATT	CTAAAAaTACaAAAAAAATT-AGG	2
**12**	11	+	20818945	20818967	CTAAAACTACCAAAAAAATT	CTAAAAaTACaAAAAAAATT-AGG	2
**13**	14	+	31521767	31521789	CTAAAACTACCAAAAAAATT	CTAAAAaTACaAAAAAAATT-AGG	2
**14**	17	+	49165992	49166014	CTAAAACTACCAAAAAAATT	CTAAAAaTACaAAAAAAATT-AGG	2
**15**	20	+	59611448	59611470	CTAAAACTACCAAAAAAATT	CTAAAAaTACaAAAAAAATT-AGG	2
**16**	1	-	92585337	92585359	CTAAAACTACCAAAAAAATT	CTAAAAaTACaAAAAAAATT-CGG	2
**17**	1	-	113206286	113206308	CTAAAACTACCAAAAAAATT	CTAAAAaTACaAAAAAAATT-AGG	2
**18**	1	-	204466246	204466268	CTAAAACTACCAAAAAAATT	CTAAAAaTACaAAAAAAATT-TGG	2
**19**	8	-	88867494	88867516	CTAAAACTACCAAAAAAATT	CTAAAAaTACaAAAAAAATT-AGG	2
**20**	11	-	32482726	32482748	CTAAAACTACCAAAAAAATT	CTAAAAaTACaAAAAAAATT-AGG	2
**21**	11	-	110069699	110069721	CTAAAACTACCAAAAAAATT	CTAAAAaTACaAAAAAAATT-AGG	2
**22**	14	-	41444071	41444093	CTAAAACTACCAAAAAAATT	CTAAAAaTACaAAAAAAATT-AGG	2
**23**	17	-	31313729	31313751	CTAAAACTACCAAAAAAATT	CTAAAAaTACaAAAAAAATT-AGG	2
**24**	20	-	33253305	33253327	CTAAAACTACCAAAAAAATT	CTAAAAaTACaAAAAAAATT-AGG	2

B

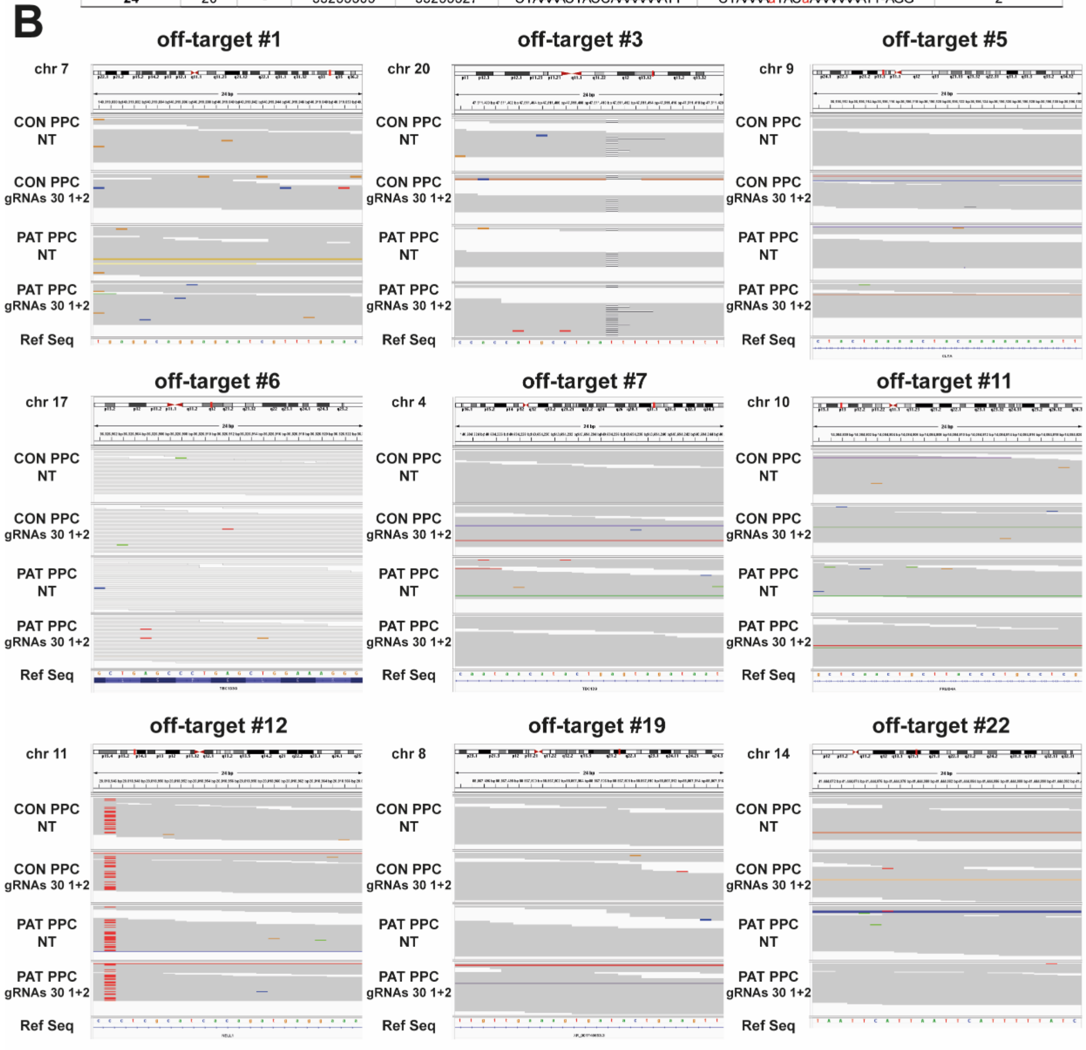

Figure S13. Summary of the analysis of the predicted off-targets for the gRNA 30-2 sequence in control and patient-derived PPCs A) Table listing the position of the predicted off-targets for gRNA 30-2 indicating the chromosome (chrom), the strand (+ or -), the start and end position, the gRNA sequence (Given query) and the off-target sequence (Actual genomic hit). The mismatches are highlighted in red. The number of mismatches is indicated in the last column. **B)** Representative Integrative Genomics Viewer (IGV) browser screenshots of four off-target sites which showed more discrepancies between non-treated samples (NT) and edited samples. In none of the potential off-target, an effect caused by the delivery of the RPNC including gRNAs 30-1 and 30-2was detected. In all cases, grey color indicated no differences while squares with different color indicated a nucleotide change (C, blue; G, black; A, green and T, red). A dark black line indicates a nucleotide missing. Some rows are

a combination of gray and colored sections rows, indicating a partial overlap of the read with the reference sequence (Ref Seq). CON PPC means isogenic control-derived PPCs. PAT PPC means patient-derived PPCs. In the case off-target #12, a SNP heterozygosity is emphasized in all samples in red (C>T).

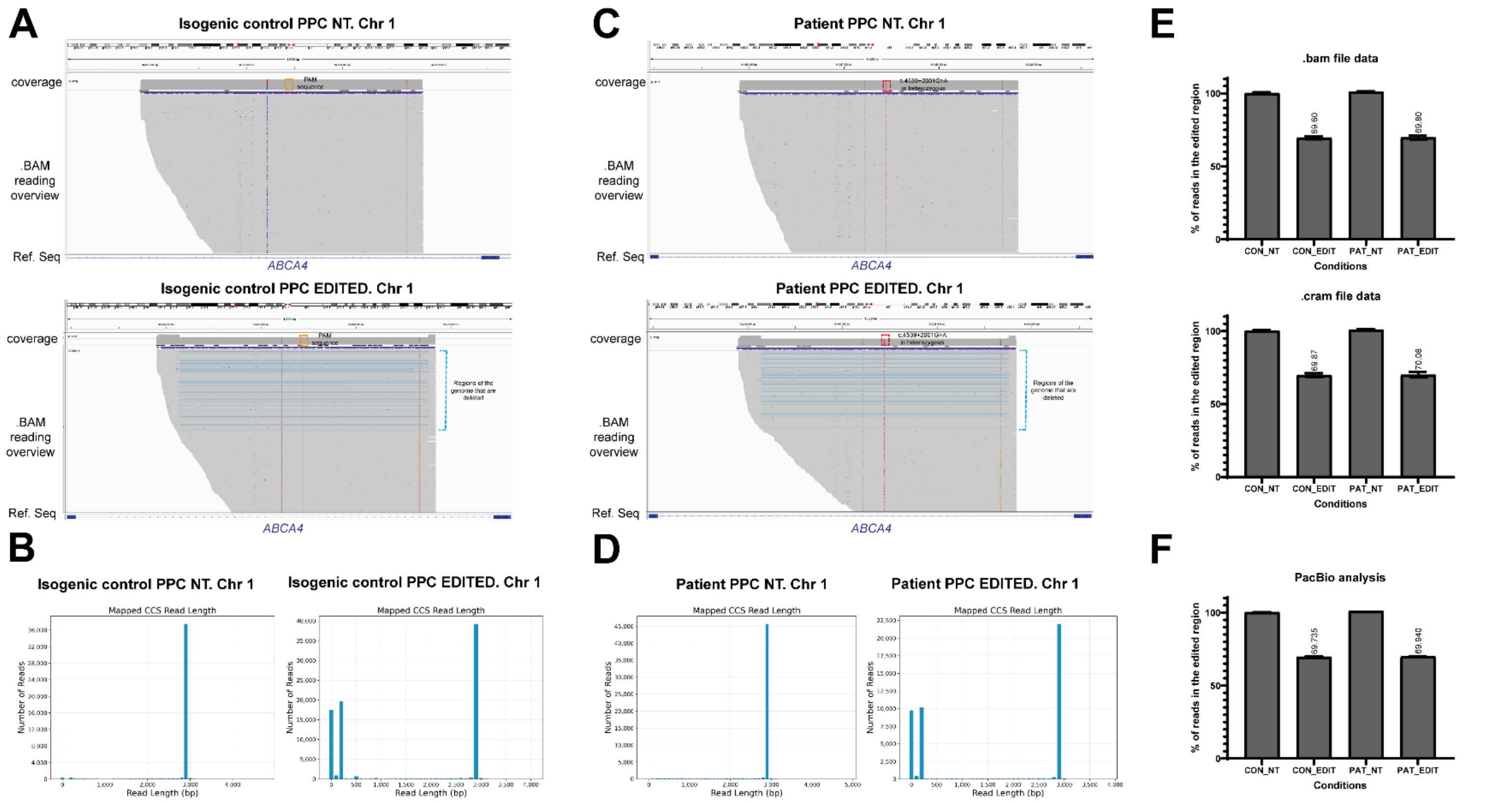

Figure S14. Overview of PacBio data on intron 30 of *ABCA4* (genome editing target) using control and patient-derived PPCs transfected with RPNCs with gRNAs 30-1 and 30-2 A) Overview of the analysis in the isogenic control PPCs using the BAM files. On top the scheme of the non-treated cells (NT) and below for the edited control PPCs. An orange square highlights the PAM sequence resulted after generating the isogenic control are shown. The blue lines represent deleted regions as a consequence of genome editing. **B)** Overview of the number of reads/fragment size of the NT (left) and EDITED (right) isogenic control PPCs **C)** Visual representation of the annotated amplicons in the patient-derived PPCs using the view of the BAM files in IGV. On top the scheme of the non-treated cells (NT) and below for the edited patient-derived PPCs are shown. A red square highlights the c.4539+2001G>A variant in heterozygosis responsible for the splicing defect. The blue lines represent deleted regions as a consequence of genome editing. **D)** Overview of the number of reads/fragment size of the NT (left) and EDITED (right) patient PPCs **E)** Graph bars of the percentage of reads in the predicted edited region by employing the ".BAM" (left) or ".CRAM" (right) files. In all cases the edited samples present a 30% reduction of the reads, corresponding to deleted reads after Cas9-RNP editing. **F**) Graph bar recapitulating the average editing taking into account the results of the ".BAM" and ".CRAM" analysis. The final editing efficiency is around 30% in both isogenic control and patient-derived PPC.

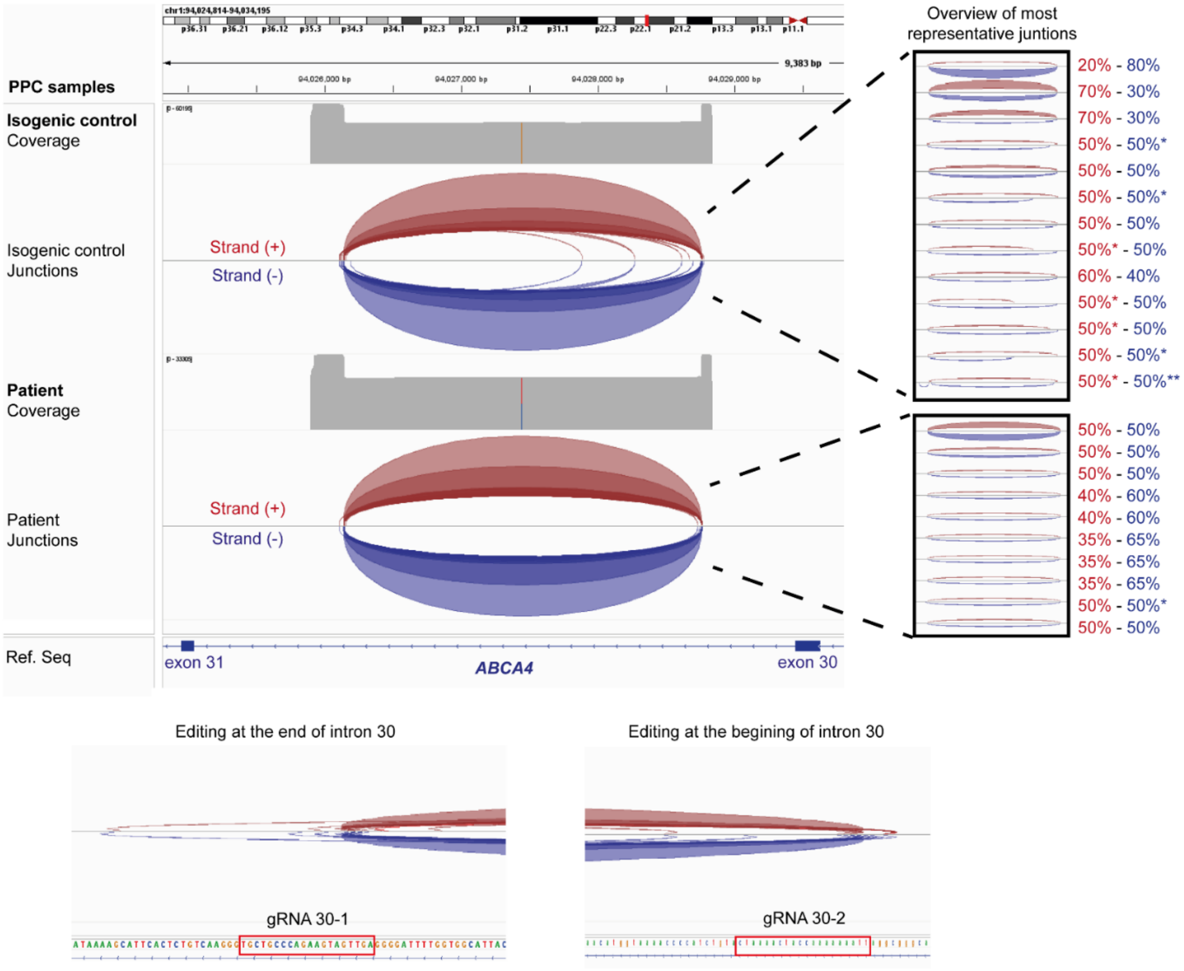

Figure S15. Overview of junctions in the isogenic control and patient PPCs. A summary of the most prevalent juctions is represented in this figure. On the right, there is a representation of different junction pattern detected in each dataset indicating the percentage of editing in the + strand (in red) and the - strand (in blue). * refers to the fact that the ending is upstream or downstream than the most prevalent cut. ** refers to double cut in that region. Ref. Seq shows the reference sequence of the reads showed on top. At the bottom of the figures is represented the end sites of the editing area highlighting the position of the gRNAs used for the PPC.

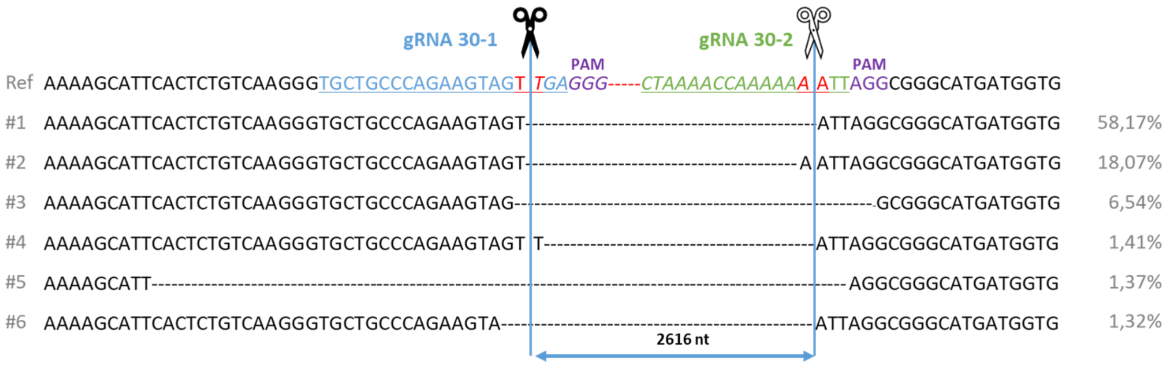

Figure S16. Overview of the repair profile after RPNC-mediated genome editing in intron 30 of *ABCA4*. This figure illustrates the six most frequent double-edited amplicons detected in both isogenic control and patient PPC lines following editing with RPNC using two specific gRNAs: gRNA 30-1 (highlighted in blue with its cutting site marked by black scissors) and gRNA 30-2 (highlighted in green with its cutting site marked by white scissors). The percentage of reads for each sequence from the pool of double-edited sequences combining both cell lines is depicted in grey on the right. The cutting positions of each gRNA on the reference sequence are highlighted in red at the top. PAM sites are highlighted in purple in the reference sequence. The top three sequences collectively accounted for approximately 82% of all reads and were consistently present in both control and patient-edited cells, showing nearly identical ratios. All detected events and number of reads for each line separately, are provided in Supplementary Table S5.

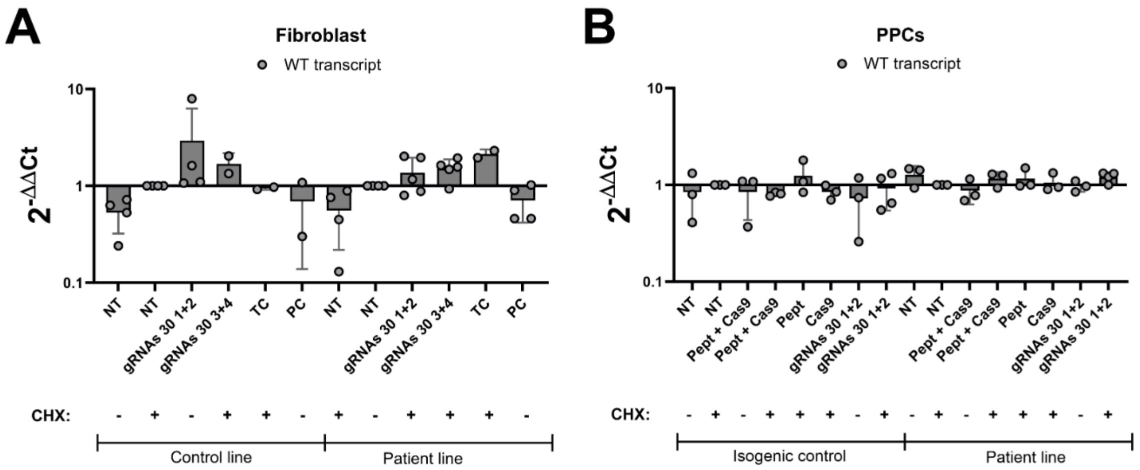

Figure S17. qPCR of the wild-type transcript after delivering the RPNC complex for intron 30 editing to fibroblast (A) or PPCs (B). Data are shown as mean ± SD of n=4 or n=3 replicates for fibroblast (A) and PPCs (B), respectively. Single data points are shown. In none of the cases, a statistical significance is detected between any of the conditions. The list of primers is indicated in Table S6.

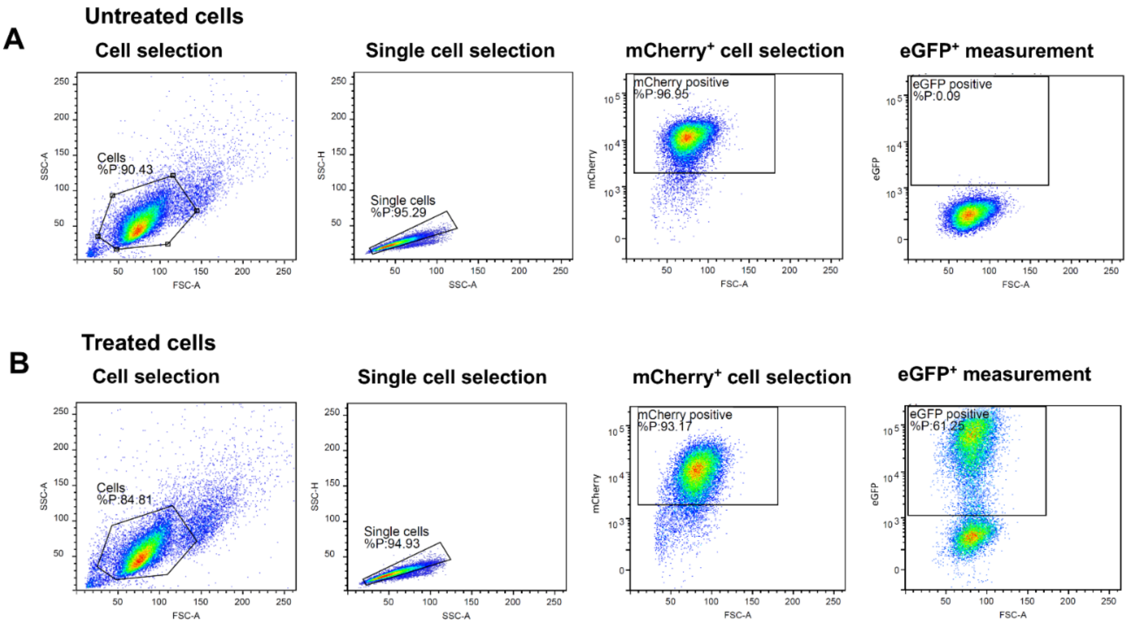

Figure S18 Flow cytometry gating strategy utilized for the assessment of gene editing in HEK293T Stoplight cells. Initially, cells were gated based on forward scatter area (FSC-A) and side scatter area (SSC-A). Subsequently, the isolation of individual cells was achieved by applying selection criteria using SSC-A and side scatter height (SSC-H) signals. Following this, the identification and measurement of Stoplight+ reporter cells was conducted using mCherry signals. Lastly, within the subset of mCherry+ cells, the percentage of eGFP+ cells were assessed. Representative flow cytometry gating strategy is shown for (A) untreated HEK293T Stoplight reporter cells and (B) or HEK293T Stoplight reporter cells treated with RPNCs for gene editing.

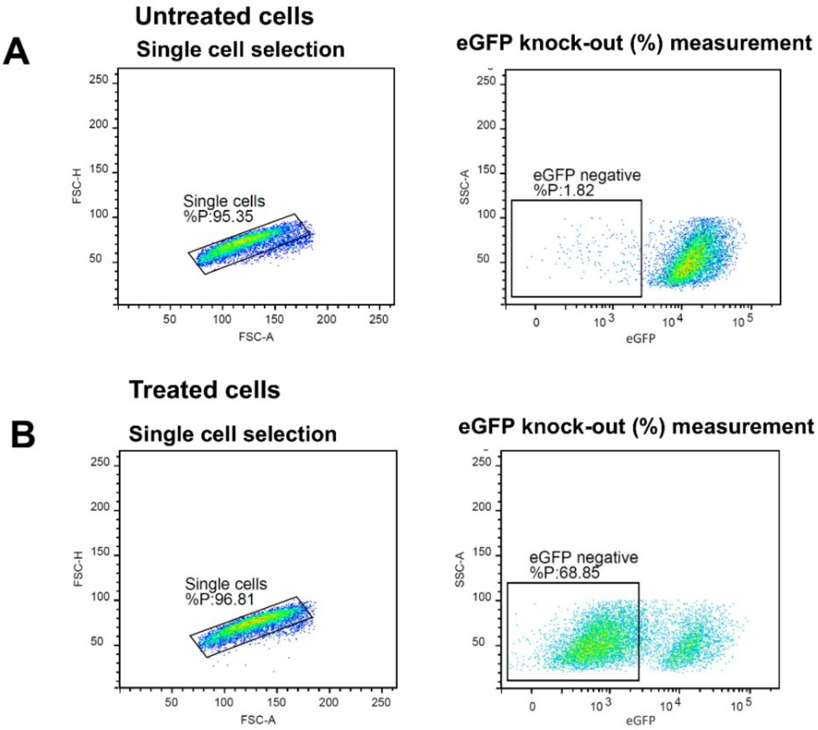

Figure S19. The gating strategy for the analysis of eGFP HEPA 1-6 cells to assess non-edited (eGFP positive), and edited (eGFP negative) cell populations. Percentage of gene editing was calculated by identified eGFP negative cells population. Representative flow cytometry gating strategy is shown for A) non-treated and B) treated with RPNCs for gene editing.

Supplemental tables.

Table S1. Summary of deep-intronic (DI) variants in intron 30 and 36 of *ABCA4*.

Chromosomal position (hg19)	DNA variant	Intron	Severity*	Number of alleles	RNA variant	Predicted protein variant	Reference
1:g.94493901T>C	c.4539+1100A>G	30	Moderate	5	r.[4539_4540ins4539+1033_4539+1100,4539_4540ins4539+989_4539+1100,=]	p.[Arg1514Valfs*31,Arg1514Glyfs*3,=]	Sangermano et al. 2019[6]
1:g.94493895G>A	c.4539+1106C>T	30	Severe	5	r.[4539_4540ins4539+1033_4539+1100,4539_4540ins4539+989_4539+1100]	p.[Arg1514Glyfs*3,Arg1514Valfs*31]	Sangermano et al. 2019[6]<br>Bauwens et al. 2019[7]
1:g.94493037C>A	c.4539+1964G>T	30	Severe	2	r.[4539_4540ins4539+1891_4539+1962]	p.(Gln1513_Arg1514ins(24))	Corradi et al. 2023[8]
1:g.94493000C>T	c.4539+2001G>A	30	Severe	84(┼)	r.[=,4539_4540ins4539+1891_4540-2162]	p.[=,Arg1514Leufs*36]	Braun et al. 2013[9]<br>Bax et al. 2015[10]<br>Albert et al. 2018[11]
1:g.94492973G>A	c.4539+2028C>T	30	Moderate	38	r.[=,4539_4540ins4539+1891_4540-2162]	p.[=,Arg1514Leufs*36]	Braun et al. 2013[9]<br>Bax et al. 2015[10]<br>Albert et al. 2018[11]
1:g.94492937G>A	c.4539+2064C>T	30	Mild	50	r.[=,4539_4540ins4539+1891_4540-2162]	p.[=,Arg1514Leufs*36]	Bauwens et al. 2019[7]
1:g.94492936G>C	c.4539+2065C>G	30	Mild	3	r.[4539_4540ins4539+1891_4539+2060,=]	p.[Arg1514Lysfs*35,=]	Khan et al. 2019[12]
1:g.94492935G>C	c.4539+2066C>G	30	Mild	1	r.[=,4539_4540ins4539+1891_4539+2065,[4254_4352del,4539_4540ins4539+1891_4539+2065],4254_4352del]	p.[=,Gln1513_Arg1514ins*6,[Ser1418_Pro1451delinsArg,Arg1514Ser*5],Ser1418_Pro1451delinsArg]	Jespersgaard et al. 2019[13]
1:g.94490612A>T	c.4540-8T>A	30	Severe	1	r.4539_4540ins4540-6_4540-1	p.Gln1513insProGln	Khan et al. 2020[14]
1:g.94485132_94485135del	c.5196+3_5196+6del	36	Severe	6	r.4849_5196del	p.Val1617_Ile1732del	Sangermano et al. 2018[15]

1:g.94484125T>C	c.5196+1013A>G	36	Severe	1	r.5196_5197ins5196+880_5196+1008	p.Met1733Valfs*2	Khan et al. 2020[4]
1:g.94484082T>C	c.5196+1056A>G	36	Severe	34	r.5196_5197ins5196+880_5196+1056	p.Met1733Valfs*2	Braun et al. 2013[9] Khan et al. 2020[4]
1:g.94484004G>C	c.5196+1134C>G	36	Moderate	1	r.[=,5196_5197ins5196+1135_5196+1212]	p.[=,Met1733Tyrfs*7]	De Angeli et al. 2023
1:g.94484001C>T	c.5196+1137G>A	36	Severe	136	r.[=,5196_5197ins5196+1140_5196+1212]	p.[=,Met1733Glufs*78]	Braun et al. 2013[9] Khan et al. 2020[4]
1:g.94483922G>T	c.5196+1216C>A	36	Moderate	4	r.[=,5196_5197ins5196+1140_5196+1212]	p.[=,Met1733Glufs*78]	Braun et al. 2013[9] Khan et al. 2020[4]
1:g.94481967C>A	c.5197-557G>T	36	Severe	1	r.5196_5197ins5197-563_5197-750	p.Met1733*	Bauwens et al. 2019[7]

Listed are variants that have been reported as pathogenic and for which induced missplicing has been confirmed either by using midigene assays or iPSC-derived models. Number of alleles reported were obtained from LOVD database for unique variants in ABCA4 (last access on May 6th, 2024) and only variants showing pseudoexon inclusion were included in the list; url: https://databases.lovd.nl/shared/variants/ABCA4/unique. ┼ means that this variants has been reported as founder mutation in the paper of Cremers, Collin and Allikments, 2020.[16] * Classification according to Khan et al. 2020[4] based on WT transcript present in either a midigene system or patient-derived retinal cells.

Table S2. Summary of the gRNAs designed and the result of the initial pair screening in HEK293T.

	Intron 30 upstream of hot-spot region				
**#**	**gRNA**	**Targeting sequence**	**PAM**	**Comments**	**Result of initial screening in HEK293T (Figure S4A)**
1	30-3	GACTTTAGAACTCCCAGGAC	AGG	Does not cover c.4539+859C>T	Selected for further experiments in fibroblasts and PPCs
2	30-6	TTCTGGACTTTAGAACTCCC	AGG	Does not cover c.4539+859C>T	Not selected
3	11	GCACAAGAATTGAACCTGGG	AGG	Discarded too many predicted off-targets	Not tested
**4**	**30-2**	**CTAAAACTACCAAAAAAATT**	**AGG**		Selected for further experiments in fibroblasts and PPCs
5	13	CATCTAAAAGTCTACGTGGG	TGG	Not covering more than 3 variants described in the intron	Not tested
6	14	GCCTCATCAGCCAATAAGGC	AGG	Not covering more than 3 variants described in the intron	Not tested
	Intron 30 downstream of hot-spot region				
**#**	**gRNA**	**Targeting sequence**	**PAM**	**Comments**	**Result of initial screening in HEK293T (Figure S4A)**
7	30-4	TCTAAGCTGGACAGACCGCG	TGG	Not covering more than 3 variants described in the intron	Selected for further experiments in fibroblasts
8	30-5	ACAGCTCCTGTGCATATGCG	TGG	Does not cover c.4540-2036C>A	Not selected for further experiments
9	E10	AGAATGGTGCGCAATAACGG	CGG	gRNA discarded. TIDE test did not detect the gRNA sequence	Not tested
**10**	**30-1**	**TGCTGCCCAGAAGTAGTTGA**	**GGG**	Selected for further experiments	Selected for further experiments in fibroblasts and PPCs
11	2	GGTGCATGGGTTTGTCCACC	AGG	Cloning on pX458 failed.	Not tested
12	4	GTTAGCTGACTTGCACTGAC	TGG	Cloning on pX458 failed.	Not tested
	Intron 36 uptream of hot-spot region				
**#**	**gRNA**	**Targeting sequence**	**PAM**	**Comments**	**Result of initial screening in HEK293T (Figure S4B)**
**13**	**36-1**	**TCAAATAATCACTGCACCGC**	**AGG**		Selected for further experiments in fibroblasts and PPCs
14	36-5	TTAGATTAACCACCGCTACA	GGG		Not selected for further experiments
15	15	ATTAGATTAACCACCGCTAC	AGG	(overlap with sequence 36-5). Discarded.	Not tested
	Intron 36 downstream of hot-spot region				
**#**	**gRNA**	**Targeting sequence**	**PAM**	**Comments**	**Result of initial screening in HEK293T (Figure S4B)**
16	36-3	TCCAAAATGATGGATCGCGG	AGG		Selected for further experiments in fibroblast
17	36-4	GTATACATCGGACGTGCTGA	GGG		Not selected for further experiments
**18**	**36-2**	**TATACATCGGACGTGCTGAG**	**GGG**		Selected for further experiments in fibroblasts and PPCs

tracrRNA sequence is property of IDT and it cannot be disclosured. The one employed in this work was purchased by the reference 1072532 (Alt-R® CRISPR-Cas9 tracrRNA; IDT).

Table S3. MFI values of RPNCs used for Figure S5D.

Sample	eGFP MFI (AU)				647 MFI (AU)			
	N1	N2	Mean	SD	N1	N2	Mean	SD
GFP Cas9/ATTO647 gRNA/C18:1-LAH5 replicate 1	6503,3	6838,5	6670,9	167,6	20713,5	23101,6	21907,6	1194,0
GFP Cas9/ATTO647 gRNA/C18:1-LAH5 replicate 2	8570,3	11314,6	9942,4	1372,1	24585,6	32518,2	28551,9	3966,3
Cas9/ ATTO488 gRNA1/ATTO647 gRNA2/C18:1-LAH5 replicate 1	3908,1	2921,0	3414,6	493,6	16828,6	12789,2	14808,9	2019,7
Cas9/ ATTO488 gRNA1/ATTO647 gRNA2/C18:1-LAH5 replicate 2	4700,3	4622,2	4661,3	39,0	22847,9	23344,7	23096,3	248,4

Table S4. Off-target list. Sheet 1 includes the complete list of predicted off-target regions associated with the gRNA gRNA 30-1. Those candidates with more than 3 mismatches are indicated in brown. Sheet 2 includes the complete list of predicted off-target regions associated with the gRNA 30-2. Those candidates with more than 3 mismatches are indicated in brown. Sheet 3 included the final selected list of off-targets for both gRNA.

Table S5. Overview of the 44 repaired sequences identified in PPCs after editing intron 30 with RPNC. All repair events identified in the double edited control and patient samples using PacBio long-read sequencing. The table includes sequences upstream and downstream of the cutting site, along with any insertions present. Columns detail the number of reads containing each mutation across both PPC lines (COMBINED) and their respective percentages (COMBINED %), as well as the number of reads and corresponding frequency in percentage in each line: isogenic control (CONTROL) and patient-derived PPCs (PATIENT). In red the nucleotides in between which the editing is expected. In italics the intronic regions of the reference. In blue substitutions found that could or could not be directly related to the editing. The PAM is depicted in purple.

Table S6. Disclosure of the sequences employed in the C18:1-LAH5 lipopeptide characterization.

**gRNA sequences employed in reporter and characterization experiments**	
**Target**	**gRNA targeting sequence**
Stoplight construct	GGACAGTACTCCGCTCGAGT
eGFP construct	GCTGAAGCACTGCACGCCGT
CCR5	TGACATCAATTATTATACAT
Non-target gRNA	GTTAATGTGGCTCTGGTTCT
ATTO550-gRNA	CCTGACAATCGATAGGTACC
ATTO647-gRNA	CTGTCCATAATTAGTCCATG
ATTO488-gRNA	TCAGTCTATACCCGATCCAC
**PCR primers for the amplification of the *CCR5* locus in the T7E1 assay**	
Primer name	Sequence 5'- 3'
CCR5 Forward	CAACAGAGCCAAGCTCTCCAT
CCR5 Reverse	CCTGGGAGAGACGCAAACAC

Table S7. **List of primers used in the genome editing of *ABCA4* intron 30 and 36.** Underlined sequence on the Site-directed primers represents the binding region complementary to the DNA strand while the non-underlined sequence is a tail complementary to the binding region of the other primer.

**Primers for site-directed mutagenesis on the in-house midigene**					
ABCA4 region	Sequence (5'-3')	Length (bp)	Tm (°C)	%GC	Amplicon size
Intron 30_SDM_1	CATCCATGTCCGATGAACTAGCTTCCAGTCCT	32	64.4	50.0	1680
Intron 30_SDM1	TAGTTCATCGGACATGGATGCATGTTCCTAG	32	61.7	45.0	

**Primers used to amplify the gRNA sequences for cloning in the pX548 plasmid**						
gRNA	Direction	Sequence (5'-3')	Length (bp)	Tm (°C)	%GC	Amplicon (bp)
gRNA 30-1	Forward	GAAAGTCAAATGGGAAAACAGG	22	51.1	41.0	253
	Reverse	AGGACTGGAAGCTAGTTCATCG	22	54.8	50.0	
gRNA 30-2	Forward	GAGTCTCTCTCTGTCACCCAGG	22	58.6	59.0	237
	Reverse	TGGTAGGCAGAAGTGGGTAGAT	22	54.8	50.0	
gRNA 30-3	Forward	CTCTCCATGTGCTGTGTCTC	20	53.8	55.0	210
	Reverse	GAGGCCCATTATCAACCACG	20	53.8	55.0	
gRNA 30-4	Forward	GGACGTCAAGGCTGTGATAC	20	53.8	55.0	533
	Reverse	GTGTTTGAGTGGCTAGGGTG	20	53.8	55.0	
gRNA 30-5	Forward	GGACGTCAAGGCTGTGATAC	20	53.8	55.0	533
	Reverse	GTGTTTGAGTGGCTAGGGTG	20	53.8	55.0	
gRNA 30-6	Forward	CTCTCCATGTGCTGTGTCTC	20	53.8	55.0	210
	Reverse	GAGGCCCATTATCAACCACG	20	53.8	55.0	
gRNA 36-1	Forward	ATCTGCTGTCCCTTGACATCTT	22	53.0	45.0	268
	Reverse	ACCTTTACCCTTTTGAAGGTGG	22	53.0	45.0	
gRNA 36-2	Forward	GTAACTTCTCAGCAAGATGCCC	22	54.8	50.0	182
	Reverse	GGTGTGAGGATTAGGTGGTGAT	22	54.8	50.0	
gRNA 36-3	Forward	AATTTACACACTTGGGGCTG	20	49.7	45.0	652
	Reverse	GGTGTGAGGATTAGGTGGTGAT	22	54.8	50.0	
gRNA 36-4	Forward	AATTTACACACTTGGGGCTG	20	49.7	45.0	652
	Reverse	GGTGTGAGGATTAGGTGGTGAT	22	54.8	50.0	
gRNA 36-5	Forward	GGCATCCTAGATTTTTATTTGCC	23	51.7	39.0	225
	Reverse	CTACCAATTCCTTTGACCCTG	21	52.4	48.0	

**Primers used for Sanger sequencing the pDONR_pX458 plasmids**				
Name	Sequence (5'-3')	Length (bp)	Tm (°C)	%GC

	SEQ_pX458_U6-Cas9_Fwd1	TCGCGTTAACGCTAGCATGGATCTC	25	59.3	52.0	
	SEQ_pX458_U6-Cas9_Fwd2	CTCTGACTGACCGCGTTACT	20	53.8	55.0	
	SEQ_pX458_U6-Cas9_Fwd3	GAGTCCTTCCTGGTGGAAGA	20	53.8	55.0	
	SEQ_pX458_U6-Cas9_Fwd4	GACATCCTGAGAGTGAACAC	20	51.8	50.0	
	SEQ_pX458_U6-Cas9_Fwd5	GAGAAGGTGCTGCCCAAGCA	20	60.0	55.9	
	SEQ_pX458_U6-Cas9_Fwd6	GACATCCAGAAAGCCCAGGT	20	53.8	55.0	
	SEQ_pX458_U6-Cas9_Fwd7	GGCAGATCACAAAGCACGTG	20	53.8	55.0	
	SEQ_pX458_U6-Cas9_Fwd8	GACAGCCCCACCGTGGCCTA	20	60.0	70.0	
	SEQ_pX458_U6-Cas9_Rev1	ACTGCCAAGTAGGAAAGTCCC	21	54.0	52.0	
	SEQ_pX458_U6-Cas9_Rev2	GTAACATCAGAGATTTTGAGACAC	24			
**DNA analysis**						
Region and gene	Oligo name	Sequence (5'-3')	Length (bp)	Tm (°C)	%GC	Amplicon (bp)
Intron 30 *ABCA4*	Intron 30_Fwd	CTCCCAGGTTCAATTCTTGTG	21	52.4	48.0	2936
	Intron 30_Rev	GGCATACAGATGACACAGTTG	21	52.4	48.0	
Intron 36 *ABCA4*	Intron 36_Fwd	ATCTGCTGTCCCTTGACATCTT	22	53.0	45.0	1840
	Intron 36_Rev	GGTGTGAGGATTAGGTGGTGAT	22	54.8	50.0	
5'UTR *RPE65*	5'UTR_Fwd	CTGAACACTCTTCTACCTGC	20	51.8	50.0	485
	Intron1_Rev	TCCAAATCTTCTTAAACCAGC	21	48.5	38.0	
**RNA analysis**						
Region and gene	Oligo name	Sequence (5'-3')	Length (bp)	Tm (°C)	%GC	Amplicon (bp)
ABCA4 PE 30	Intron 30_Fwd	CTCCACTCAAAACCCTCCAG	20	53.8	55	271
	Exon 31_Rev	ATGTTCCTGTCCGTCAGGTC	20	53.8	55	
ACTB	Exon 3_Fwd	ACTGGGACGACATGGAGAAG	20	60.5	55.0	398
	Exon 4_rev	TCTCAGCTGTGGTGGTGAAG	20	60.5	55.0	
ABCA4 exon 30-31	Exon 30_Fwd	AAACATCACCCAGCTGTTCC	20	51.8	50.0	173
	Exon 31_Rev	GAAGTCGGAGATGTTCCTGTC	21	54.4	52.0	
ABCA4 exon 27-35	Exon 27_Fwd	GCTGCTGGTCAAGAGATTCC	20	60.5	55.0	807
	Exon 35_Rev	GCATGCCAGCCTTTGTTATT	20	56.4	45.0	
ABCA4 exon 34-38	Exon 34_Fwd	ACCTGATTTCCTTAAACATCTAGAAA	26	60.1	31.0	608
	Exon 38_Rev	TTGATGCCGATGAACAGATT	20	54.3	40.0	
RHO-exon 5 (midigenes)	Exon 5_Fwd	ATCTGCTGCGGCAAGAAC	18	64.7	55.6	140
	Exon 5_Rev	AGGTGTAGGGGATGGGAGAC	20	64.5	60.0	

Table S8. qPCR primers list. All qPCR reactions were conducted using an annealing temperature of 60°C

Gene	Sequence (5'-3')	Length	GC%	Amplicon size (bp)
ABCA4_wt transcript_F	AGCACCAGGGAGAAGCTC	18	58.0	81
ABCA4_wt transcript_R	GCGCTGTGTTCTCTGGGG	18	61.0	
ABCA4 intron 30 PE_F (fibroblast)	CTCACCATGCTGCCAGAGT	19	58.0	97
ABCA4 intron 30 PE_R (fibroblast)	CCTTGGGTCCTTCTTTTGGG	20	55.0	
ABCA4 intron 30 PE_F (PPCs)	TAGTGTTGGTCCTTGGTCCC	20	59.0	85
ABCA4 intron 30 PE_R (PPCs)	CTTGTAGAATTTCCGTGCTGC	21	58.0	
*PAX6*_qPCR_F	GCTGCAAAGAAATAGAACATCC	22	41.0	111
*PAX6*_qPCR_R	TTGGCTGCTAGTCTTTCTCG	20	50.0	
*CRX*_qPCR_F	CCCCAGTGTGGATCTGATG	19	58.0	116
*CRX*_qPCR_R	CAAACAGTGCCTCCAGCTC	19	58.0	
*RECOVERIN*_qPCR_F	ACACCAAGTTCTCGGAGGAG	20	55.0	108
*RECOVERIN*_qPCR_R	ACTTGGCGTAGATGCTCTGG	20	55.0	
*OPN1SW*_qPCR_F	TTCTTCTCCAAGAGTGCTTGC	21	48.0	97
*OPN1SW*_qPCR_R	CCTTCCCACACACCATCTTC	20	55.0	
*OTX2*_qPCR_F	TATCTTAAGCAACCGCCTTACG	22	45.0	75
*OTX2*_qPCR_R	GGAGGGGTGCAGCAAGTC	18	67.0	
*RPE65*_qPCR_F	TTACTACGCTTGCACAGAGACC	22	50.0	105
*RPE65*_qPCR_R	GCCCCATTGACAGAGACATAG	21	52.0	
*OCT3/4*_qPCR_F	GTTCTTCATTCACTAAGGAAGG	22	41.0	101
*OCT3/4*_qPCR_R	CAAGAGCATCATTGAACTTCAC	22	41.0	
*GUSB*_qPCR_F	AGAGTGGTGCTGAGGATTGG	20	55.0	80
*GUSB*_QPCR_R	CCCTCATGCTCTAGCGTGTC	20	60.0	
*ABCA4*_qPCR_F	CATCCTGTTCCACCACCTCA	20	55.0	113
*ABCA4*_qPCR_R	CTGTGTCCTCCAACATGGCT	20	55.0	

Table S9. List of antibodies used for Western Blot analysis study.

**Primary Antibodies**	**Target protein**	**Produced by**	**Antibody Catalog #**	**Host**	**Diluent**	**WB dilution**
	ABCA4	Abcam	ab72955	Rabbit	2.5% Not-fally blotto Milk	1:1000
	β-TUBULIN	Abcam	ab15568	Rabbit		1:1000
**Secondary antibodies**	**Target host**	**Produced by**	**Antibody Catalog #**	**Host/ Fluorophore**	**Diluent**	**WB dilution**
	Rabbit	Molecular Probes	A21076	Goat/Alex Flulor 680	2.5% Not-fally blotto Milk	1:10000
	Rabbit	Li-COR biosciences	LI 926-32211	Goat/IRDye800		1:10000

References:

1. de Jong, OG, Murphy, DE, Mager, I, Willms, E, Garcia-Guerra, A, Gitz-Francois, JJ, Lefferts, J, Gupta, D, Steenbeek, SC, van Rheenen, J*, et al.* (2020). A CRISPR-Cas9-based reporter system for single-cell detection of extracellular vesicle-mediated functional transfer of RNA. *Nat Commun* **11**: 1113.
2. Haer-Wigman, L, den Ouden, A, van Genderen, MM, Kroes, HY, Verheij, J, Smailhodzic, D, Hoekstra, AS, Vijzelaar, R, Blom, J, Derks, R*, et al.* (2022). Diagnostic analysis of the highly complex OPN1LW/OPN1MW gene cluster using long-read sequencing and MLPA. *NPJ Genom Med* **7**: 65.
3. Robinson, JT, Thorvaldsdottir, H, Winckler, W, Guttman, M, Lander, ES, Getz, G, and Mesirov, JP (2011). Integrative genomics viewer. *Nat Biotechnol* **29**: 24-26.
4. Khan, M, Arno, G, Fakin, A, Parfitt, DA, Dhooge, PPA, Albert, S, Bax, NM, Duijkers, L, Niblock, M, Hau, KL*, et al.* (2020). Detailed Phenotyping and Therapeutic Strategies for Intronic ABCA4 Variants in Stargardt Disease. *Mol Ther Nucleic Acids* **21**: 412-427.
5. Suarez-Herrera, N, Li, CHZ, Leijsten, N, Karjosukarso, DW, Corradi, Z, Bukkems, F, Duijkers, L, Cremers, FPM, Hoyng, CB, Garanto, A, and Collin, RWJ (2024). Preclinical Development of Antisense Oligonucleotides to Rescue Aberrant Splicing Caused by an Ultrarare ABCA4 Variant in a Child with Early-Onset Stargardt Disease. *Cells* **13**.
6. Sangermano, R, Garanto, A, Khan, M, Runhart, EH, Bauwens, M, Bax, NM, van den Born, LI, Khan, MI, Cornelis, SS, Verheij, J*, et al.* (2019). Deep-intronic ABCA4 variants explain missing heritability in Stargardt disease and allow correction of splice defects by antisense oligonucleotides. *Genet Med*.
7. Bauwens, M, Garanto, A, Sangermano, R, Naessens, S, Weisschuh, N, De Zaeytijd, J, Khan, M, Sadler, F, Balikova, I, Van Cauwenbergh, C*, et al.* (2019). ABCA4-associated disease as a model for missing heritability in autosomal recessive disorders: novel noncoding splice, cis-regulatory, structural, and recurrent hypomorphic variants. *Genet Med*.
8. Corradi, Z, Khan, M, Hitti-Malin, R, Mishra, K, Whelan, L, Cornelis, SS, Group, AB-S, Hoyng, CB, Kampjarvi, K, Klaver, CCW*, et al.* (2023). Targeted sequencing and in vitro splice assays shed light on ABCA4-associated retinopathies missing heritability. *HGG Adv* **4**: 100237.

9. Braun, TA, Mullins, RF, Wagner, AH, Andorf, JL, Johnston, RM, Bakall, BB, Deluca, AP, Fishman, GA, Lam, BL, Weleber, RG*, et al.* (2013). Non-exomic and synonymous variants in ABCA4 are an important cause of Stargardt disease. *Hum Mol Genet* **22**: 5136-5145.

10. Bax, NM, Sangermano, R, Roosing, S, Thiadens, AA, Hoefsloot, LH, van den Born, LI, Phan, M, Klevering, BJ, Westeneng-van Haaften, C, Braun, TA*, et al.* (2015). Heterozygous deep-intronic variants and deletions in ABCA4 in persons with retinal dystrophies and one exonic ABCA4 variant. *Hum Mutat* **36**: 43-47.

11. Albert, S, Garanto, A, Sangermano, R, Khan, M, Bax, NM, Hoyng, CB, Zernant, J, Lee, W, Allikmets, R, Collin, RWJ, and Cremers, FPM (2018). Identification and Rescue of Splice Defects Caused by Two Neighboring Deep-Intronic ABCA4 Mutations Underlying Stargardt Disease. *Am J Hum Genet* **102**: 517-527.

12. Khan, M, Cornelis, SS, Khan, MI, Elmelik, D, Manders, E, Bakker, S, Derks, R, Neveling, K, van de Vorst, M, Gilissen, C*, et al.* (2019). Cost-effective molecular inversion probe-based ABCA4 sequencing reveals deep-intronic variants in Stargardt disease. *Hum Mutat* **40**: 1749-1759.

13. Jespersgaard, C, Fang, M, Bertelsen, M, Dang, X, Jensen, H, Chen, Y, Bech, N, Dai, L, Rosenberg, T, Zhang, J*, et al.* (2019). Molecular genetic analysis using targeted NGS analysis of 677 individuals with retinal dystrophy. *Sci Rep* **9**: 1219.

14. Khan, M, Cornelis, SS, Pozo-Valero, MD, Whelan, L, Runhart, EH, Mishra, K, Bults, F, AlSwaiti, Y, AlTalbishi, A, De Baere, E*, et al.* (2020). Resolving the dark matter of ABCA4 for 1054 Stargardt disease probands through integrated genomics and transcriptomics. *Genet Med* **22**: 1235-1246.

15. Sangermano, R, Khan, M, Cornelis, SS, Richelle, V, Albert, S, Garanto, A, Elmelik, D, Qamar, R, Lugtenberg, D, van den Born, LI*, et al.* (2018). ABCA4 midigenes reveal the full splice spectrum of all reported noncanonical splice site variants in Stargardt disease. *Genome Res* **28**: 100-110.

16. Cremers, FPM, Lee, W, Collin, RWJ, and Allikmets, R (2020). Clinical spectrum, genetic complexity and therapeutic approaches for retinal disease caused by ABCA4 mutations. *Prog Retin Eye Res* **79**: 100861.
